# *Aurora A* Functional Single Nucleotide Polymorphism (SNP) Correlates With Clinical Outcome in Patients With Advanced Solid Tumors Treated With Alisertib, an Investigational Aurora A Kinase Inhibitor

**DOI:** 10.1016/j.ebiom.2017.10.015

**Published:** 2017-10-16

**Authors:** Huifeng Niu, Hyunjin Shin, Feng Gao, Jacob Zhang, Brittany Bahamon, Hadi Danaee, Bohuslav Melichar, Russell J. Schilder, Robert L. Coleman, Gerald Falchook, Antoine Adenis, Kian Behbakht, Angela DeMichele, Elizabeth Claire Dees, Kimberly Perez, Ursula Matulonis, Piotr Sawrycki, Dirk Huebner, Jeffrey Ecsedy

**Affiliations:** a Millennium Pharmaceuticals, Inc., Cambridge, MA, USA; b Department of Oncology, Palacky University Medical School and Teaching Hospital, Olomouc, Czech Republic; c Department of Medical Oncology, Thomas Jefferson University Hospital, Philadelphia, PA, USA; d The University of Texas MD Anderson Cancer Center, Houston, TX, USA; e Sarah Cannon Research Institute at HealthONE, Denver, CO, USA; f Centre Oscar Lambret, Lille, France; g Department of Gynecologic Oncology, University of Colorado School of Medicine, Aurora, CO, USA; h Abramson Cancer Center of the University of Pennsylvania, Philadelphia, PA, USA; i UNC Lineberger Comprehensive Cancer Center, Chapel Hill, NC, USA; j Department of Medical Oncology, Dana-Farber Cancer Institute, Boston, MA, USA; k Gynecologic Oncology Program, Dana-Farber Cancer Institute, Boston, MA, USA; l Department of Oncology and Chemotherapy, L. Rydygiera District Hospital, Torun, Poland

**Keywords:** Aurora A kinase inhibitor, Alisertib, SNP, Prognosis, Predictive biomarker, Correlative analysis

## Abstract

**Background:**

Alisertib (MLN8237) is an investigational, oral, selective Aurora A kinase inhibitor. *Aurora A* contains two functional single nucleotide polymorphisms (SNPs; codon 31 [F/I] and codon 57 [V/I]) that lead to functional changes. This study investigated the prognostic and predictive significance of these SNPs.

**Methods:**

This study evaluated associations between *Aurora A* SNPs and overall survival (OS) in The Cancer Genome Atlas (TCGA) database. The *Aurora A* SNPs were also evaluated as predictive biomarkers for clinical outcomes to alisertib in two phase 2 studies (NCT01045421 and NCT01091428). *Aurora A* SNP genotyping was obtained from 85 patients with advanced solid tumors receiving single-agent alisertib and 122 patients with advanced recurrent ovarian cancer treated with alisertib plus weekly paclitaxel (n = 62) or paclitaxel alone (n = 60). Whole blood was collected prior to treatment and genotypes were analyzed by PCR.

**Findings:**

TCGA data suggested prognostic significance for codon 57 SNP; solid tumor patients with VV and VI alleles had significantly reduced OS versus those with II alleles (HR 1.9 [VI] and 1.8 [VV]; p < 0.0001). In NCT01045421, patients carrying the VV alleles at codon 57 (n = 53, 62%) had significantly longer progression-free survival (PFS) than patients carrying IV or II alleles (n = 32, 38%; HR 0.5; p = 0.0195). In NCT01091428, patients with the VV alleles at codon 57 who received alisertib plus paclitaxel (n = 47, 39%) had a trend towards improved PFS (7.5 months) vs paclitaxel alone (n = 32, 26%; 3.8 months; HR 0.618; p = 0.0593). In the paclitaxel alone arm, patients with the VV alleles had reduced PFS vs modified intent-to-treat (mITT) patients (3.8 vs 5.1 months), consistent with the TCGA study identifying the VV alleles as a poor prognostic biomarker. No significant associations were identified for codon 31 SNP from the same data set.

**Interpretation:**

These findings suggest that *Aurora A* SNP at codon 57 may predict disease outcome and response to alisertib in patients with solid tumors. Further investigation is warranted.

## Highlights

• *Aurora A* contains two single nucleotide polymorphisms (SNPs) at codons 31 and 57 that lead to functional amino acid changes
• We evaluated the potential prognostic and predictive value of these SNPs and revealed the SNP at codon 57 may predict disease outcome and response to Alisertib in patients with solid tumors

Alisertib, an investigational Aurora A kinase inhibitor, was evaluated in clinical trials and showed clinically meaningful benefit in patients with solid tumors. Two coding region single nucleotide polymorphisms (SNPs) in the *Aurora A* gene have been reported to be associated with functional changes of the protien. Here we assessed the prognostic and predictive value of *Aurora A* SNPs in a range of solid tumors. The results suggest that codon 57 SNP may predict disease outcome and response to alisertib in patients. These findings warrant further investigation and may ultimately provide a patient selection strategy for alisertib in certain cancers.

## 1 Introduction

Aurora A kinase (AAK), a member of the conserved serine/threonine protein kinase family, is a key mitotic regulator with a critical role in centrosome maturation and separation, spindle assembly, chromosome alignment, and cytokinesis (Barr and Gergely, 2007, Marumoto et al., 2003). Overexpression and/or amplification of AAK has been observed in a variety of cancers (Dar et al., 2008, Hoque et al., 2003, Mazumdar et al., 2009) and tends to be associated with a poor patient outcome (Nikonova et al., 2013). Furthermore, AAK inhibition results in mitotic progression abnormalities leading to cell death (Marumoto et al., 2003, Gorgun et al., 2010, Zhou et al., 2013). As such, AAK represents an attractive target for anti-cancer therapy.

*Aurora A* is an oncogene located on chromosome 20q13.2, a locus frequently amplified in solid tumors (Bischoff et al., 1998). Two functional single nucleotide polymorphisms (SNPs) (rs2273535 and rs1047972) in *Aurora A* gene, located at codon 31 and codon 57 in the NH_2_-terminal region of the Aurora A protein, have been reported to be associated with functional consequences and increased cancer risk (Chen et al., 2007, Chen et al., 2015, Ewart-Toland et al., 2005, Kimura et al., 2005, Miao et al., 2004). The T91A SNP at codon 31 results in a phenylalanine to isoleucine (F31I) change; homozygous T/T at this locus codes for FF, T/A leads to FI, and A/A codes for II. This SNP is associated with increased frequency of aneuploidy in human colon tumors (Ewart-Toland et al., 2005), increased breast cancer risk, especially in Asian patients (Dai et al., 2004), and low penetrance cancer susceptibility for other cancer types including lung and oesophageal cancer (Ewart-Toland et al., 2005). The G169A SNP at codon 57 results in a valine to isoleucine amino acid substitution (V57I); homozygous A/A genotype at this locus codes for II, A/G leads to IV, and G/G codes for VV. Studies indicate the II variant reduces AAK activity (Kimura et al., 2005), and may be a protective factor for risk of developing cancer, especially in Caucasian patients and those with breast cancer (Dai et al., 2004). Studies investigating the association between *Aurora A* SNPs and clinical outcomes are limited. The heterozygous A/T at codon 31 (FI) has been shown to be associated with a significantly higher risk of tumor relapse, shorter disease-free survival, and shorter median survival time in oesophageal cancer patients treated with preoperative chemoradiation (Pan et al., 2012). In particular, in patients treated with cisplatin-based chemoradiation, this genotype at codon 31 was associated with poor response and shorter survival (Pan et al., 2012). The variant I31/V57 haplotype also carried a significant risk for a low rate of complete response and higher recurrence rate (Pan et al., 2012).

The investigational oral agent alisertib (MLN8237) is a selective, small-molecule AAK inhibitor that has demonstrated preclinical activity against a broad range of tumor types (Manfredi et al., 2011, Sehdev et al., 2013). In pilot studies, alisertib demonstrated antitumor activity with manageable toxicity in patients with solid tumors (Cervantes et al., 2012, Melichar et al., 2015) and haematological malignancies (Goldberg et al., 2014, Kelly et al., 2014). Two phase 2 clinical trials investigating single-agent alisertib (NCT01045421) in patients with advanced solid tumors, or alisertib in combination with weekly paclitaxel (NCT01091428) in patients with recurrent ovarian cancer, have recently been completed (Coleman et al. 2014 ESMO Congress) (Melichar et al., 2015). In NCT01045421, an overall response rate (ORR) of 13% was reported in the response-evaluable population, with patients in the breast cancer and small-cell lung cancer (SCLC) cohorts showing response rates of 18% and 21%, respectively (Melichar et al., 2015). In NCT01091428, alisertib plus weekly paclitaxel significantly increased median progression free survival (PFS) compared with paclitaxel alone (hazard ratio [HR] 0.740 [80% CI 0.563, 0.971]) (Falchook et al. manuscript submitted in parallel).

Although alisertib has demonstrated promising clinical activity, its efficacy has been variable (Friedberg et al., 2014, Goldberg et al., 2014, Matulonis et al., 2012, Melichar et al., 2015). Therefore, it is important to identify potential predictive marker(s) that could be used to select patients likely to benefit from alisertib treatment (Hadley and Hendricks, 2014, Kap et al., 2016). This study aimed to assess the potential association between two *Aurora A* SNP genotypes (rs2273535 and rs1047972) at codons 31 and 57, and clinical endpoints in patients from the NCT01045421 and NCT01091428 trials.

## 2 Methods

### 2.1 Study Design and Participants

The Cancer Genome Atlas (TCGA) database was searched for 10,403 patients with solid tumors who had recorded information on the SNPs of interest. RNA sequencing (RNA-seq) and overall survival (OS) data from 10,034 patient samples were available to assess the association between *Aurora A* SNP genotypes and OS. Since the SNPs are not somatic mutations and there are no reported cases with somatic mutations at those sites, variants found from RNA-Seq can be thought of as germline SNPs. The genotypes of a particular SNP were called based on the allele fraction of its SNP allele.

Details of both phase 2 alisertib studies have been published previously (Melichar et al., 2015); additional study information (patient consent, treatment regimen, and study endpoints) can be found in the appendix. In brief, NCT01045421 was an open-label, multicentre study of single-agent alisertib in 249 adult patients with advanced, relapsed solid tumors including breast cancer, small cell lung cancer (SCLC), non-SCLC (NSCLC), head and neck squamous-cell carcinoma (HNSCC), and gastro-oesophageal adenocarcinoma (GE). NCT01091428 was a randomised, open-label study of alisertib plus weekly paclitaxel compared with paclitaxel alone in 142 adult patients with previously treated recurrent epithelial ovarian, fallopian tube, or primary peritoneal cancer. Correlative analyses of Aurora A SNPs and clinical efficacy outcomes were conducted in the subgroup of patients (85 of 249) from the study NCT01045421 and the subgroup of patients (122 of 142) from the study NCT01091428.

### 2.2 Procedures

To analyze *Aurora A* SNP data in TCGA, SNPs were genotyped based on RNA-seq results from patients (see appendix for details). Once genotypes at the SNP sites were assigned to each sample, the potential association between genotype and OS was investigated. At each SNP site (i.e. codon 31 and codon 57), survival curves for the three strata (homozygous reference, heterozygous, and homozygous SNP) were plotted and HR were calculated using the Cox proportional hazard model with respect to homozygous reference (FF at codon 31 and II at codon 57), with no clinical covariates considered; the p-value was computed using χ2 (Marumoto et al., 2003) test.

Whole blood samples were collected prior to administration of alisertib or paclitaxel, and genomic DNA was isolated from peripheral blood mononuclear cells. *Aurora A* SNP genotypes were analyzed by real-time polymerase chain reaction (PCR; see appendix for details). Whole exome next-generation sequencing (NGS) was also carried out on a subset of tumor samples (from 47 patients) from NCT01045421.

Details of the statistical methodology utilized in both phase 2 studies have been previously described (Melichar et al., 2015). In this analysis, a correlative study was performed to assess the relationship of *Aurora A* SNP genotypes with alisertib treatment outcomes (PFS, best tumor size change, and best response). For NCT01045421, a Cox regression model was used to analyze possible associations between *Aurora A* SNPs and PFS, stratified by tumor indication. The p-value was adjusted for tumor indication. Analysis of variance (ANOVA) was used to analyze best tumor size change, adjusting for tumor indication and baseline tumor size. A simple Χ2 (Marumoto et al., 2003) test on the contingency table was used to test the independence between genotype and patient response status. For NCT01091428, PFS was analyzed using log-rank test to compare treatment arms in different populations.

## 3 Results

### 3.1 Prognostic Implication of *Aurora A* SNP at Codon 57

We analyzed *Aurora A* SNP data derived from 10,403 cancer patients with 33 different cancer types in the TCGA database; VV were the most frequent alleles at codon 57 (5649). The distribution of *Aurora A* SNPs in patients with different tumor types was similar to population data in the dbSNP database (data not shown). A similar pattern of *Aurora A* SNP distribution at codons 57 and 31 was seen in the NCT01045421 and NCT01091428 populations (Supplementary Fig. 1).

**Fig. 1:**
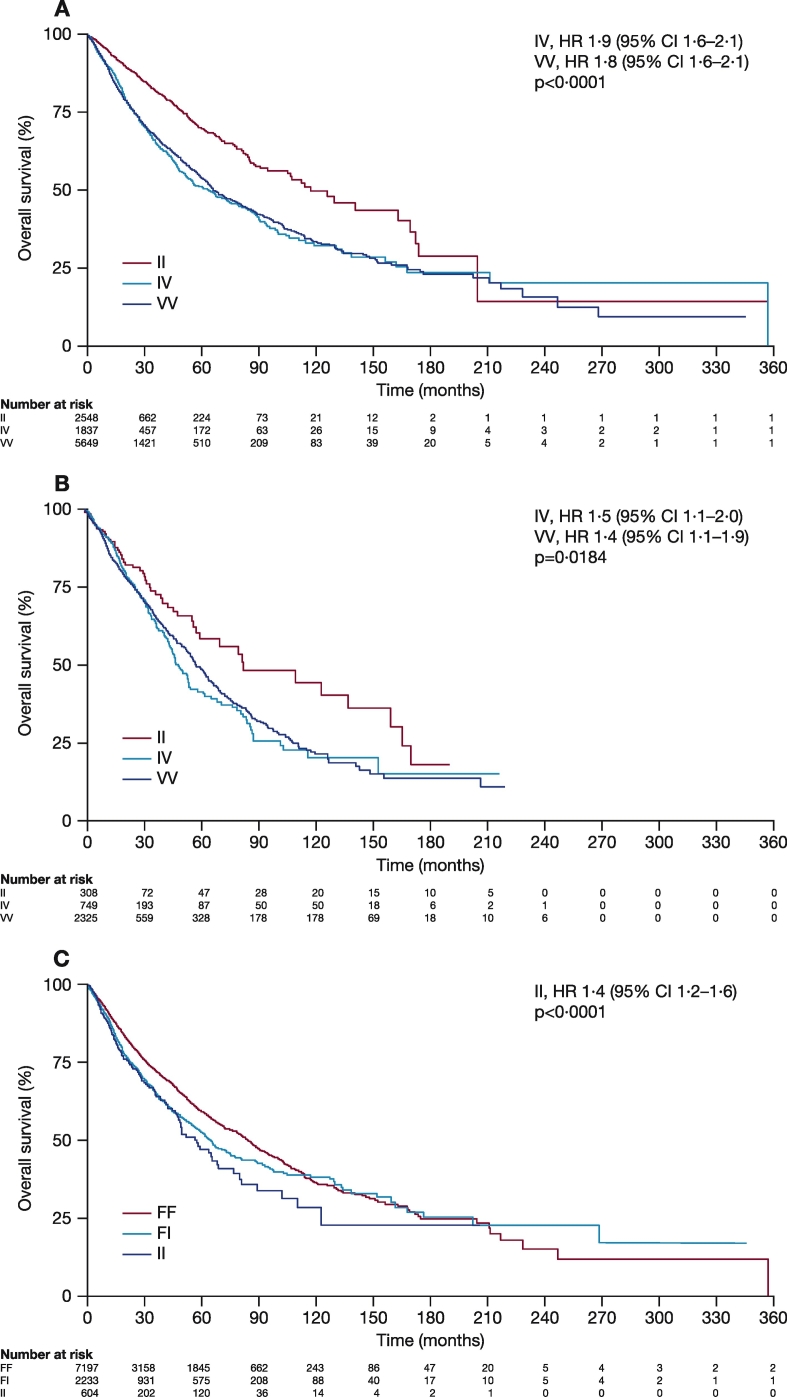
OS in TCGA patients with solid tumors according to *Aurora A* SNPs at A) codon 57 (all solid tumors), B) codon 57 (breast, HNSCC, NSCLC, GE, and ovarian cancers), and C) codon 31 (all solid tumors). CI, confidence interval; GE, gastro-oesophageal adenocarcinoma; HNSCC, head and neck squamous-cell carcinoma; HR, hazard ratio; NSCLC, non-small cell lung cancer; OS, overall survival; SNP, single nucleotide polymorphism; TCGA, The Cancer Genome Atlas.

Using the data in the TCGA database, we carried out a correlative study to assess the potential association between *Aurora A* SNP genotypes and OS from 10,034 patients whose survival data were available for the analysis. The prognostic significance of codon 57 SNP was evident across tumor types and stages. In patients with solid tumors, II alleles at codon 57 (2548) were associated with improved OS compared with IV (1837) or VV (5649) alleles (IV: HR 1.9 [95% CI 1.6, 2.1]; VV: HR 1.8 [95% CI 1.6, 2.1]; p < 0.0001); OS was similar in patients with IV or VV alleles (Fig. 1A). In patients with tumors relevant to the phase 2 studies (breast, HNSCC, NSCLC, GE, and ovarian), II at codon 57 (308) was again associated with improved OS compared with IV (749) or VV (2325) (IV: HR 1.5 [95% CI 1.1, 2.0]; VV: HR 1.4 [95% CI 1.1, 1.9]; p = 0.0184; Fig. 1B). Patients with solid tumors carrying II at codon 31 (604) demonstrated a decrease in OS compared with patients with FF alleles (7197) (HR 1.4 [95% CI 1.2, 1.6]; p < 0.0001; Fig. 1C), although the number of patients with II alleles was limited (604).

### 3.2 Predictive Potential of *Aurora A* SNPs for Alisertib Treatment Outcomes

We assessed potential associations between *Aurora A* SNPs and alisertib clinical efficacy (PFS, best tumor size change, and best response). In total, 85 patients from the NCT01045421 study and 122 patients from the NCT01091428 study (62 in the alisertib plus paclitaxel arm and 60 in the paclitaxel alone arm) with evaluable clinical data were genotyped for *Aurora A* SNPs. No significant associations were identified for codon 31 SNP (Supplementary Fig. 2); therefore, this SNP was not described further in this study.

Demographics and baseline characteristics for response-evaluable patients with genotyping data available for codon 57 are shown in Table 1. Overall, characteristics were comparable with the overall populations for each study. Although numbers were small, a greater proportion of patients in NCT01091428 with II alleles (n = 4) rather than VV or IV at codon 57 (n = 118) had a long period to relapse since receiving prior platinum therapy, consistent with the TCGA data indicating that II alleles at codon 57 are a positive prognostic marker.

**Table 1:** Demographics and baseline characteristics for response-evaluable patients with genotyping data in phase 2 alisertib studies.

NCT01045421	Overall mITT population (*N* = 249)	Codon 57 SNP genotype			
		VV (n = 53)	IV (*n* = 27)	II (n = 5)	Total (*N* = 85)
Median age, years (range)	61 (30–88)	57 (30–88)	62 (33–76)	63 (36–68)	57 (30–88)
Male/female, n (%)	141 (57)/108 (43)	33 (62)/20 (38)	18 (67)/9 (33)	1 (20)/4 (80)	52 (61)/33 (39)
Race, n (%)					
White	231 (93)	45 (85)	25 (93)	5 (100)	75 (88)
Black/African American	12 (5)	5 (9)	1 (4)	0	6 (7)
Other/not reported	6 (2)	3 (6)	1 (4)	0	4 (5)

NCT01091428	Overall mITT population (*N* = 142)	Codon 57 SNP genotype			
		VV (*n* = 94)	IV (n = 24)	II (n = 4)	Total (*N* = 122)
Median age, years(range)	62 (30–81)	62 (30–81)	65 (48–81)	62 (52–74)	62 (30–81)
Male/female, n (%)	0/142 (100)	0/94 (100)	0/24 (100)	0/4 (100)	0/122 (100)
Race, n (%)					
White	121 (85)	77 (82)	22 (92)	4 (100)	103 (84)
Black/African American	8 (6)	7 (7)	1 (4)	0	8 (7)
Other/not reported	13 (9)	10 (11)	1 (4)	0	11 (9)
Months of relapse since prior platinum therapy, IVR n (%)					
Refractory	18 (13)	9 (10)	5 (21)	0	14 (11)
0–≤6 months	71 (50)	54 (57)	11 (46)	1 (25)	66 (54)
> 6–12 months	53 (37)	31 (33)	8 (33)	3 (75)	42 (34)

IVR, interactive voice response; mITT, modified intent-to-treat; SNP, single nucleotide polymorphism.

**Fig. 2:**
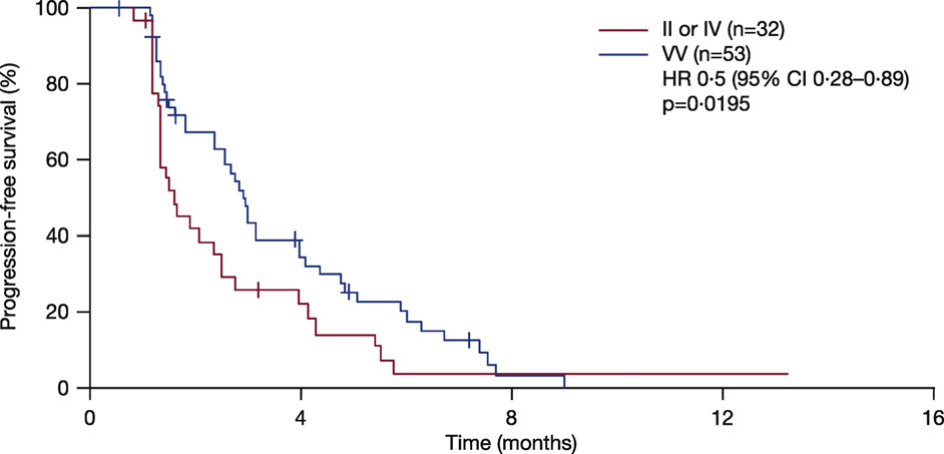
PFS according to *Aurora A* SNP at codon 57 in patients treated with alisertib in the NCT01045421 study. p-Value calculated using proportional hazard model and stratified by tumor indication. CI, confidence interval; HR, hazard ratio; PFS, progression-free survival; SNP, single nucleotide polymorphism.

**Fig. 3:**
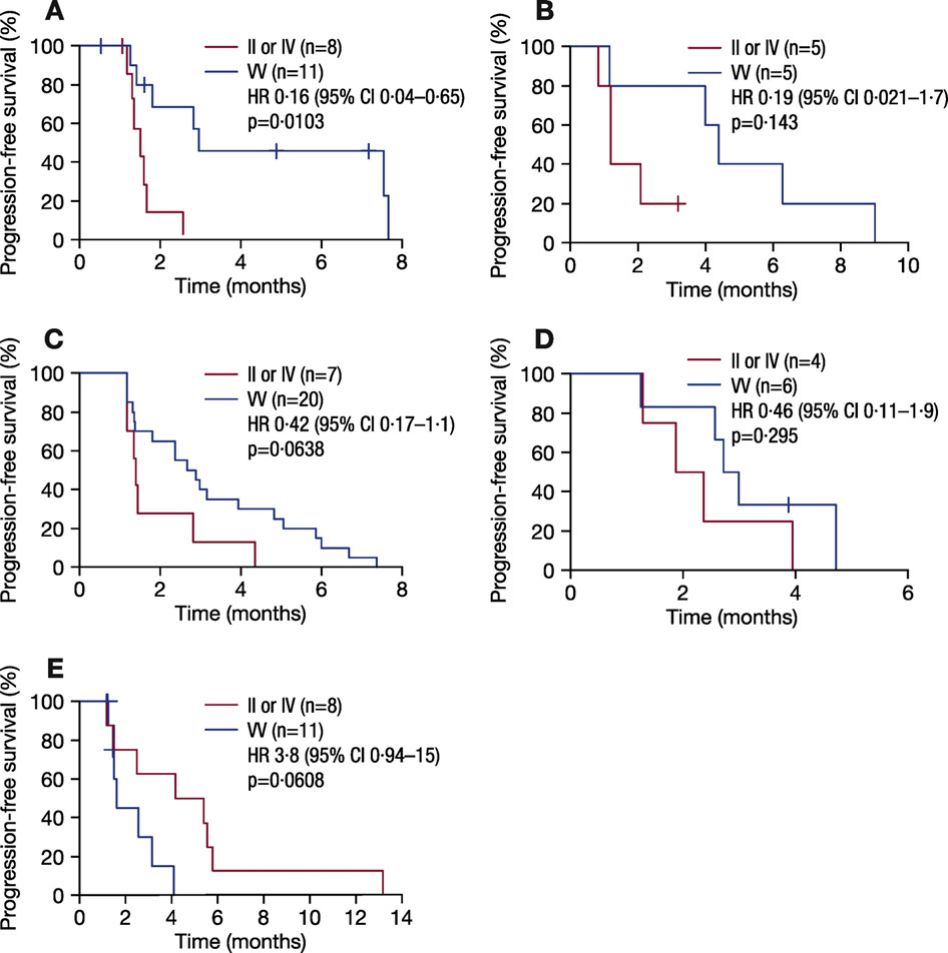
PFS according to *Aurora A* SNP at codon 57 in patients treated with alisertib in the NCT01045421 study with A) GE, B) SCLC, C) HNSCC, D) NSCLC, or E) breast cancer. CI, confidence interval; GE, gastro-oesophageal adenocarcinoma; HNSCC, head and neck squamous-cell carcinoma; HR, hazard ratio; NSCLC, non-small cell lung cancer; SCLC, small cell lung cancer; SNP, single nucleotide polymorphism.

**Fig. 4:**
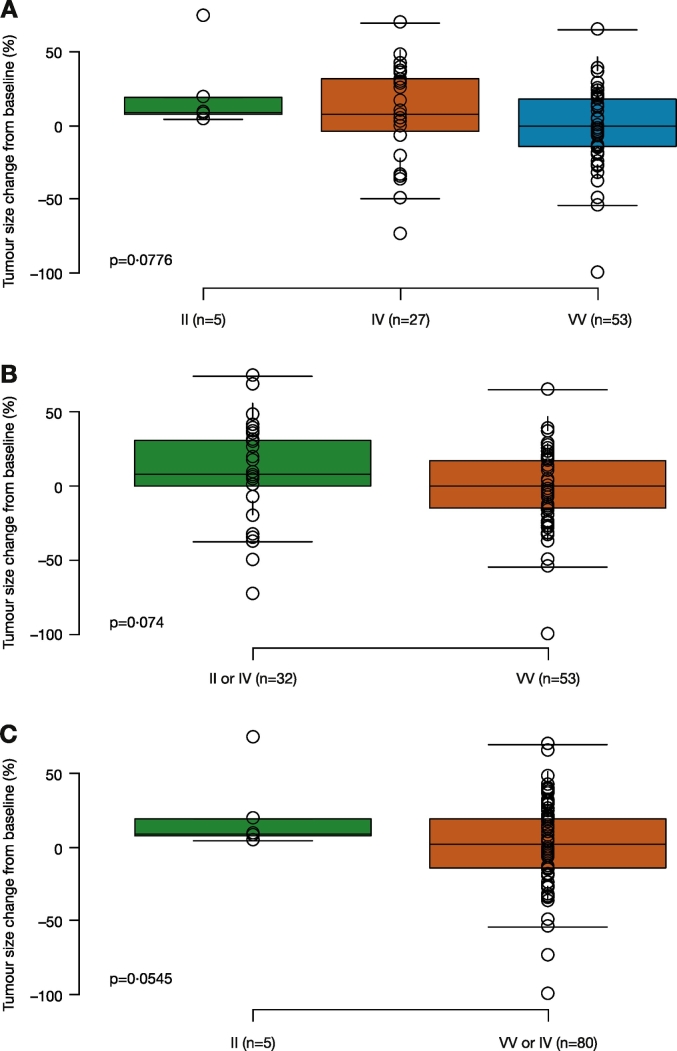
Percentage tumor size change from baseline in patients treated with alisertib in the NCT01045421 study according to *Aurora A* SNP at codon 57: A) II vs IV vs VV, B) II or IV vs VV, and C) II vs IV or VV. p-Value calculated by ANOVA, adjusted for tumor indication, SNP, single nucleotide polymorphism.

**Fig. 5:**
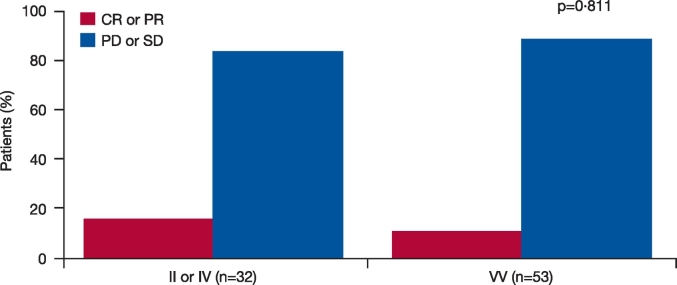
Best response (CR + PR; RECIST v1.1) in patients treated with alisertib in the NCT01045421 study according to *Aurora A* SNP at codon 57. CR, complete response; PD, progressive disease; PR, partial response; RECIST, Response Evaluation Criteria In Solid Tumors; SD, stable disease; SNP, single nucleotide polymorphism.

As no somatic mutations have been found in the *Aurora A* gene thus far, in TCGA or the phase 2 clinical trials, germline SNPs are identical to tumor DNA. In this analysis, it was assumed that SNPs detected in blood samples will be the same as those in the patients' tumors, and may lead to differential Aurora A kinase activity. Correlative analysis showed that patients treated with alisertib in the NCT01045421 study who carried VV alleles at codon 57 had significantly increased PFS compared with patients with the IV or II alleles (HR 0.5 [95% CI 0.28, 0.89]; p = 0.0195; Fig. 2). Similar PFS improvements were seen among different tumor types in a subgroup analysis; GE patients who had VV alleles had a significant improvement in PFS compared with patients with the II or IV alleles (HR 0.16 [95% CI 0.04, 0.65]; p = 0.0103; Fig. 3A), although total patient numbers were small. Similarly, patients with SCLC, HNSCC, or NSCLC who had VV alleles also had improved PFS compared with patients with the II or IV alleles (Figs. 3B–D). In contrast, patients treated with alisertib who had breast cancer and were carrying VV alleles were associated with a trend towards reduced PFS compared with patients with II or IV alleles (HR 3.8 [95% CI 0.94, 15]; p = 0.0608; Fig. 3E). It is worth noting that among 11 VV breast cancer patients analyzeanalyzed, five had triple-negative breast cancers, a subtype previously associated with a minimal response to alisertib (Melichar et al., 2015). Overall, there was a trend towards improved tumor size reduction from baseline in patients with VV vs IV vs II alleles at codon 57 (p = 0.0776; p-value adjusted by tumor type; Fig. 4A); also when VV was compared with IV or II alleles (p = 0.074; p-value adjusted by tumor type; Fig. 4B). The same was true when IV was grouped with VV and compared with II (p = 0.0545; p-value adjusted by tumor type; Fig. 4C). Best response (CR + PR) assessed by Response Evaluation Criteria In Solid Tumors (RECIST) was comparable between groups (5 [16%] vs 6 [11%] patients; p = 0.811; Fig. 5).

**Fig. 6:**
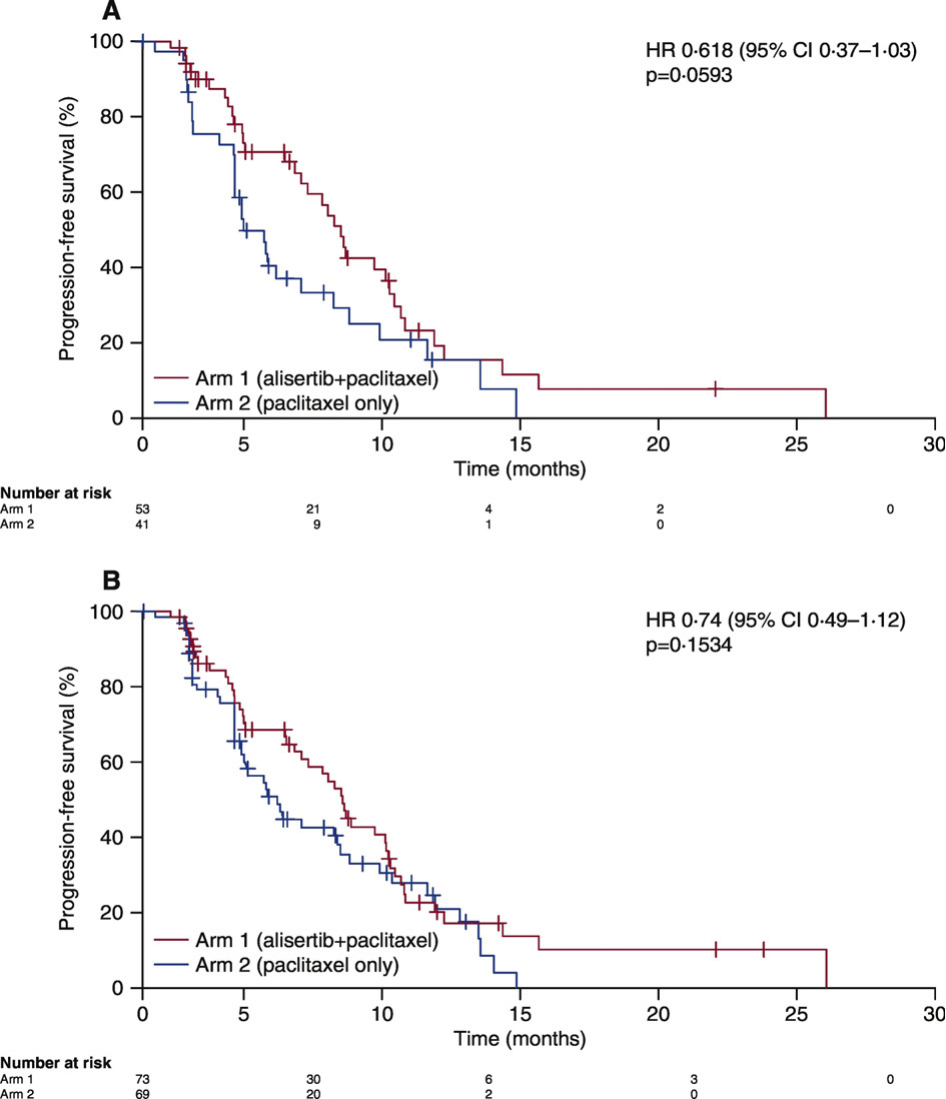
PFS in patients receiving alisertib plus paclitaxel vs paclitaxel alone in the NCT01091428 study: A) patients with VV alleles at *Aurora A* codon 57, and B) overall mITT study population. CI, confidence interval; HR, hazard ratio; mITT, modified intent-to-treat; PFS, progression-free survival.

In NCT01091428, the 47 patients with the VV alleles at codon 57 who received alisertib plus paclitaxel demonstrated an improved PFS compared with the 32 patients who received paclitaxel alone (7.5 vs 3.8 months; HR 0.618 [95% CI 0.37, 1.03]; p = 0.0593; Fig. 6A). This PFS benefit was greater than in the overall modified intent-to-treat (mITT) population (7.6 vs 5.1 months; HR 0.74 [95% CI 0.49, 1.12]; p = 0.1534; Fig. 6B). Paclitaxel-treated VV patients had reduced PFS compared with the overall mITT population (3.8 vs 5.1 months), which further implicates VV as a poor prognostic biomarker. Similarly, although the number of patients was small (n = 3; n = 1), those with II alleles showed improved PFS in both arms (alisertib plus paclitaxel 9.2 months; paclitaxel alone 13.3 months).

Intra-arm comparisons showed that patients treated with alisertib plus paclitaxel who had VV alleles had a similar outcome to patients with IV and II alleles (median PFS 7.5 vs 7.6 months; p = 0.651; Supplementary Fig. 3A). Patients treated with paclitaxel alone who had VV alleles had 3.8 months median PFS compared with 5.1 months in patients with IV and II genotypes (p = 0.400; Supplementary Fig. 3B). There was no significant association between codon 57 *Aurora A* SNP and objective response to alisertib (data not shown).

## 4 Discussion

The results from this retrospective correlative study suggest that the *Aurora A* SNP at codon 57 may have prognostic and predictive value for cancer patients, and may provide a possible patient selection strategy for treatment with alisertib in some cancers.

In all tumor types analyzed in the TCGA database, regardless of stage and treatment history, patients carrying the II alleles at codon 57 had a significantly improved OS compared with patients with the VV and VI alleles (p < 0.0001). Therefore, the II alleles at codon 57 could be considered a favorable prognostic biomarker in cancer patients.

The predictive potential of this *Aurora A* SNP for alisertib response was supported by correlatives studies in two independent trials investigating alisertib as a single agent or in combination with paclitaxel in solid tumors. In the NCT01045421 study, which enrolled patients with breast cancer, SCLC, NSCLC, HNSCC, and GE, patients with VV alleles at codon 57 had significantly improved PFS compared with patients with the IV or II alleles. Although patient numbers were small, PFS improvements were demonstrated across different tumor types, with the exception of breast tumors. This could be due to the fact that among the 11 VV breast cancer patients analyzed, five had triple-negative breast cancers, while only two out of eight II/IV patients had triple negative breast cancers. As previously reported in heavily-pretreated breast cancer patients, hormone receptor-positive and human epidermal growth factor receptor 2 (HER2)-positive subgroups responded to alisertib treatment, but minimal activity was seen in the triple-negative subtype (Melichar et al., 2015). Patient subgrouping based on HER2 and estrogen receptor (ER) status may override the ability of the *Aurora A* SNP to predict sensitivity to alisertib.

In the NCT01091428 study, ovarian cancer patients treated with alisertib plus paclitaxel did not demonstrate a difference in PFS within the arm of patients with VV versus IV or II, which is likely due to the fact that IV and II patients have a better prognosis in responding to paclitaxel. However, patients with VV demonstrated a trend towards improved PFS with alisertib plus paclitaxel versus paclitaxel alone, with a slightly greater apparent treatment effect than in the mITT population. The apparent greater PFS benefit with alisertib plus paclitaxel versus paclitaxel alone in VV compared with IV or II patients is mainly due to poorer outcomes with paclitaxel in VV-carrying patients, although this may be a multifactorial phenomenon. These results suggest that alisertib may provide greater benefit to patients with the VV alleles, which is a biomarker predictive of poor response to standard-of-care agents. Interestingly, in both studies, treatment of patients carrying the *Aurora A* VV SNP at codon 57 with alisertib was not associated with an increase in response rate. Additional studies would be useful in order to understand the underlying mechanism for the effect of alisertib on PFS, but not objective response. A preliminary analysis to assess associations between *Aurora A* SNP status and safety outcomes found no significant correlation (data not shown).

It has been reported that Aurora A V57V has higher kinase activity than Aurora A I57I (Kimura et al., 2005); overexpression and amplification of Aurora A are frequently found in solid tumors and are associated with poor disease outcomes (Chen et al., 2015, Chen et al., 2007, Ewart-Toland et al., 2005, Hoque et al., 2003, Kimura et al., 2005, Pan et al., 2012). In multiple preclinical models, *Aurora A* has been identified as an oncogene that drives tumor growth and confers chemoresistance (Sun et al., 2015, Zhang et al., 2014). Consistent with these findings, the presence of VV alleles at codon 57 (conferring higher kinase activity) correlated with poor OS in our analysis of TCGA data. Alisertib, as an Aurora A protein kinase inhibitor, may deliver greater benefit in patients with VV alleles and therefore high Aurora A kinase activity. In contrast, patients with II alleles with a good prognosis may have low Aurora A kinase activity in tumors and for this reason they derive less benefit from Aurora A inhibition by alisertib. In the NCT01091428 study, II patients treated with alisertib plus paclitaxel had reduced PFS compared with paclitaxel treatment alone (9.2 vs. 13.3 months).

This study has a number of limitations. Firstly, these correlative studies were carried out retrospectively with a limited sample size. The patient population with blood samples available for genotyping in NCT01045421 was relatively small (approximately one quarter of the entire study population). Response measures were not confirmed by independent review in either study and a limited number of solid tumor types were assessed. Prospective validation of these observations in an independent clinical study will be needed to establish the predictive value of the *Aurora A* codon 57 SNP. Moreover, the predictive value of this SNP for alisertib activity may be dependent on additional factors, including cancer subtype, whether alisertib is administered as single or combination therapy, risk stratification, and other predictive biomarkers. It remains to be seen whether these findings can be applied to other tumor types including haematological malignancies.

In conclusion, *Aurora A* SNP genotype was associated with differential outcomes to alisertib treatment and may have potential as a biomarker in patients with solid tumors; further investigation is warranted. These findings may ultimately provide a patient selection strategy for alisertib treatment.

## Funding

Millennium Pharmaceuticals, Inc., Cambridge, MA, USA, a wholly owned subsidiary of Takeda Pharmaceutical Company Limited.

## Role of the funding source

The study was funded by Millennium Pharmaceuticals, Inc., Cambridge, MA, USA, a wholly owned subsidiary of Takeda Pharmaceutical Company Limited. Employees participated in trial design, study monitoring, data collection, analysis, and interpretation, and manuscript writing. The corresponding author had full access to all the study data and had final responsibility for the decision to submit for publication.

## Prior presentation of this study

Hiufeng Niu, Hyunjin Shin et al. An *Aurora A* single nucleotide polymorphism (SNP) correlates with clinical responses to alisertib in patients (pts) with advanced solid tumors. Presented as a poster at the 2016 American Society of Clinical Oncology annual meeting, 2–6 June, Chicago, IL, USA (J Clin Oncol 2016;34:abstr 2583).

## Contributors

**Study conception and design** – HN, HS, BB, RJS, RLC, KB, AD, UM, DH, JE.
**Collection and assembly of data** – HN, HS, BB, HD, BM, RLC, GF, KB, AD, UM.
**Data analysis and interpretation** – HN, HS, JZ, HD, BM, RJS, RLC, GF, AA, KB, AD, UM, DH, JE.
**Provision of study materials or patients** – RJS, RLC, AA, UM.
**Drafting or revising the manuscript** – all authors.
**Review and approval of the final version of the manuscript** – all authors.

## Declaration of interests

RC.

**Employment**: BB, HD, DH are employees of Millennium Pharmaceuticals, Inc., a wholly owned subsidiary of Takeda Pharmaceutical Company Limited.
**Financial activity during the submitted work**: AD reports a grant for institutional support for clinical trial from Millennium;
**Financial activity outside the submitted work**: BM reports personal fees and honoraria for lectures and advisory boards for Roche, AstraZeneca, Novartis, Pfizer, Bristol Myers Squibb, Astellas, Merck, MSD, Janssen, Bayer and Amgen and reports travel support from Roche, Novartis, Bristol Myers Squibb, and Merck; RJS reports personal fees and ad hoc advisory boards from Millennium, Clovis, Vertex, Merrimack and Merck Serono and chair and IDSM for Merck Serono; GF reports personal fees and travel reimbursement from Millennium; AD reports grants for institutional support for clinical trial from Novartis, Pfizer, Calithera and Genentech, scientific advisory board from Pfizer, Novartis, Calithera and educational program participation from Pfizer; ECD reports personal fees, consulting and endpoint adjudication committee activity from Novartis and contract for trial from Merck, Cerulean, Bayer, and Pfizer.
**Intellectual property**: HN, HS, and JZ have a provisional patent application for ‘Method for identification, evaluation and treatment of patients having cancers’.
**No conflicts of interest to disclose**: AD, KB, KP, UM, JE having nothing to disclose.

## Appendix A Supplementary data

Supplementary material 1 Image 1

Supplementary material 2 Image 2
