# Supplementary material for: Aurora A Functional Single Nucleotide Polymorphism (SNP) Correlates With Clinical Outcome in Patients With Advanced Solid Tumors Treated With Alisertib, an Investigational Aurora A Kinase Inhibitor
Source: eBioMedicine. 2017 Oct 16;25:50–7. doi: 10.1016/j.ebiom.2017.10.015 (PMC5704062; doi:10.1016/j.ebiom.2017.10.015)
Supplement: Supplementary file 1 — Supplementary material 1 [file mmc1.docx]

***Aurora A* functional single nucleotide polymorphism (SNP) correlates with clinical outcome in patients with advanced solid tumours treated with alisertib, an investigational Aurora A kinase inhibitor**

**WEB APPENDIX (supplementary materials – available online only)**

| **Table of Contents** |  |
| --- | --- |
| **Part I – Additional methods** | **p. 2–3** |
| **Part II – Supplementary Figures** | **p. 4–6** |
| Figure 1. Distribution of *Aurora A* genotypes at codons 57 and 31 in A) NCT01045421 and B) NCT01091428. | **p. 4** |
| Figure 2. PFS in alisertib-treated patients in the NCT01045421 study according to *Aurora A* SNP at codon 31. | **p. 5** |
| Figure 3. PFS according to codon 57 SNP in patients receiving A) alisertib plus paclitaxel and B) paclitaxel alone in the NCT01091428 study. | **p. 6** |

**Part I – Additional methods**

**Analysis of *Aurora A* SNP data in The Cancer Genome Atlas (TCGA) database.**

| SNPs were genotyped based on RNA sequencing (RNA-Seq); lower and upper cut-offs for allele fraction were selected in a data-driven manner, which resulted in cut-offs of 15% and 85%, respectively. That is, if an allele fraction was 0–15%, the genotype was called as ‘homozygous reference’ (FF at codon 31 and II at codon 57), if between 15–85%, ‘heterozygous’ (FI at codon 31 and IV at codon 57), and if between 85–100%, ‘homozygous SNP’ (II at codon 31 and VV at codon 57). |
| --- |

**Analysis of *Aurora A* SNP genotypes by real-time polymerase chain reaction (PCR).**

| Whole blood samples were collected and genomic DNA was isolated. *Aurora A* SNP genotypes were analysed by real-time PCR at Labcorp/Covance (Seattle, WA), a Clinical Laboratory Improvement Amendments-certified laboratory. Briefly, two *Aurora A*-coding SNPs (rs2273535 and rs1047972) were tested using a catalogue probe set and a custom ordered assay, respectively, from Applied Biosystems (ABI, Foster City, CA). All clinical DNA samples were run at 50ng/reaction in duplicate on an ABI 7900HT Fast Real-time PCR System (ABI) at 35 PCR cycles. Additionally, the following samples were included in every run at the appropriate concentration: a synthetic positive control template (SPCT) for each SNP, a no template control (NTC), as well as HapMap DNA samples (Coriell Insititute, Camden, NJ). Genotyping calls were made by importing the SDS files into Genotyper 1.0.1 (ABI) and the autocalling feature of Genotyper was utilized on all analysed samples. If the SNP call was ‘undetermined’ by the autocalling feature, they were confirmed by manual inspection of the plots. |
| --- |

**Additional details of NCT01045421 and NCT01091428 phase 2 studies.**

| **Patient consent**  Both studies were conducted in accordance with the Declaration of Helsinki and Good Clinical Practice guidelines, and the protocols were approved by local ethics committees at each participating centre. All patients provided written informed consent.  **Treatment regimen**  In NCT01045421, patients received alisertib 50 mg twice-daily for 7 days, followed by a 14-day treatment-free interval, in 21-day cycles. In NCT01091428, patients received either alisertib 40 mg twice-daily on days 1–3, 8–10, and 15–17 plus 60 mg/m^2^ paclitaxel via a 1-hour intravenous infusion on days 1, 8, 15, or 80 mg/m^2^ paclitaxel intravenously on days 1, 8, and 15, in 28-day cycles. In both studies, response was assessed by investigators after every 2 cycles (approximately 6 or 8 weeks) according to Response Evaluation Criteria In Solid Tumors (RECIST) v1.1.28. Computed Tomography/Magnetic Resonance Imaging scans were used to assess tumour size.  **Study endpoints**  The primary endpoint for the NCT01045421 trial was overall response rate (ORR); secondary efficacy endpoints included duration of response (DOR), time to progression (TTP), and progression-free survival (PFS). The primary endpoint for NCT01091428 was PFS; secondary efficacy endpoints included ORR, DOR, TTP, and overall survival (OS). An exploratory endpoint in both studies was the genotyping of peripheral blood samples for two *Aurora A* SNPs located at codons 31 and 57 (rs2273535 and rs1047972, respectively) and assessment of their potential association with clinical efficacy (PFS, best tumour size change, and best response). |
| --- |

**Part II – Supplementary figures**

**Supplementary Figure 1. Distribution of *Aurora A* genotypes at codons 57 and 31 in A) NCT01045421 and B) NCT01091428.**


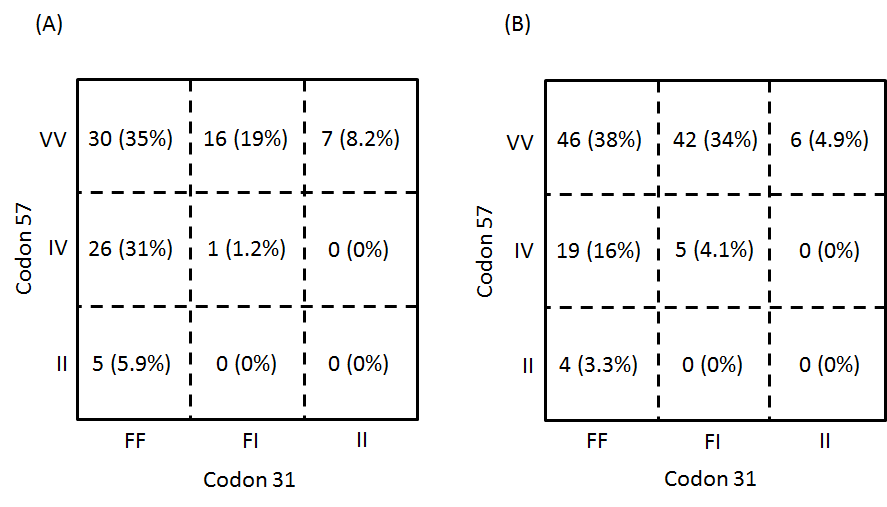


**Supplementary Figure 2: PFS in alisertib-treated patients in the NCT01045421 study according to *Aurora A* SNP at codon 31.**


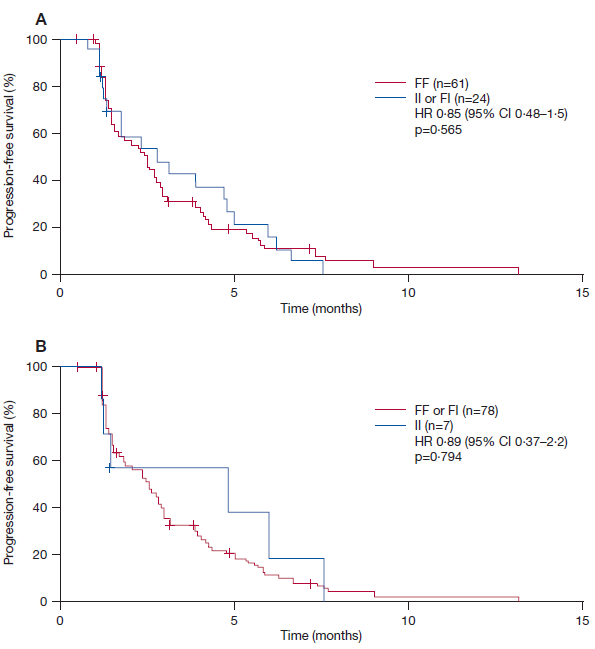


CI, confidence interval; HR, hazard ratio; PFS, progression-free survival; SNP, single nucleotide polymorphism.

**Supplementary Figure 3: PFS according to codon 57 SNP in patients receiving A) alisertib plus paclitaxel and B) paclitaxel alone in the NCT01091428 study.**


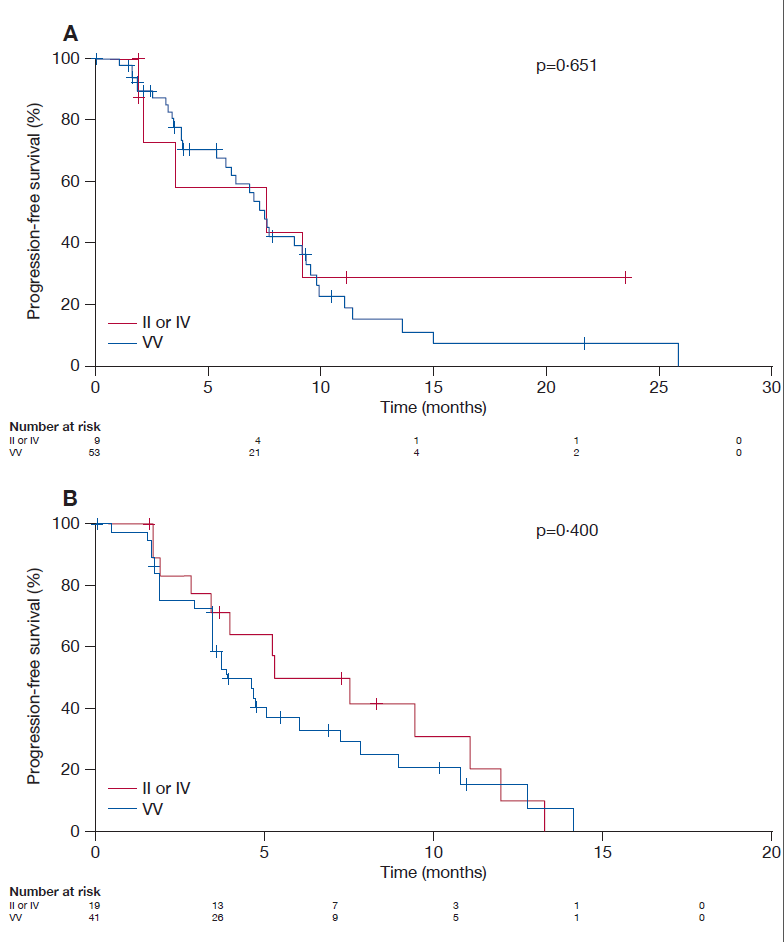


PFS, progression-free survival; SNP, single nucleotide polymorphism.
