# Supplementary material for: Aurora A Functional Single Nucleotide Polymorphism (SNP) Correlates With Clinical Outcome in Patients With Advanced Solid Tumors Treated With Alisertib, an Investigational Aurora A Kinase Inhibitor
Source: eBioMedicine. 2017 Oct 16;25:50–7. doi: 10.1016/j.ebiom.2017.10.015 (PMC5704062; doi:10.1016/j.ebiom.2017.10.015)
Supplement: Supplementary file 2 — Supplementary material 2 [file mmc2.pdf]

CLINICAL STUDY PROTOCOL C14008 AMENDMENT 3

MLN8237

Randomized Phase 2 Study of MLN8237, an Aurora A Kinase Inhibitor, Plus Weekly Paclitaxel or Weekly Paclitaxel Alone in Patients With Recurrent Epithelial Ovarian, Fallopian Tube, or Primary Peritoneal Cancer, Preceded by a Phase 1 Portion in Patients With Ovarian or Breast Cancer

**Protocol Number:**	C14008
**Indication:**	Recurrent epithelial adenocarcinoma of ovarian, tubal, or peritoneal origin; adenocarcinoma of the breast (allowed only in the Phase 1 portion)
**Phase:**	1/2
**Sponsor:**	Millennium Pharmaceuticals, Inc.
**EudraCT Number:**	2009-011428-79
**Therapeutic Area:**	Oncology

Protocol History

Original	Version 1	30 June 2009
Original	Version 2	13 July 2009
Amendment 1		15 December 2009
Amendment 2		15 November 2010
Amendment 3		30 November 2010

Millennium Pharmaceuticals, Inc.
40 Landsdowne Street
Cambridge, MA USA 02139
Telephone: +1 (617) 679-7000

Approved by:

[redacted] [redacted] 01 Dec 2010

Chair, Clinical Review Boa[redacted] *Date DD Month YYYY*

[redacted] 01 DEC 2010

Vice President, Clinical Research
Oncology Clinical Research

Signature

Date DD Month YYYY

Confidentiality Statement
The information contained herein is confidential and the proprietary property of Millennium Pharmaceuticals and any unauthorized use or disclosure of such information without the prior written authorization of Millennium Pharmaceuticals is expressly prohibited.

Rationale for Amendment 3

The primary purpose of this amendment is to make required updates to the criteria for removing patients from the study. This amendment allows a patient who experiences progressive disease (PD), defined by CA-125 criteria or imaging results described in the protocol (Section 15.4), to continue protocol treatment if tolerated and if the investigator determines it is in the patient's best interest upon review and agreement by the medical monitor. Based on review and comments by the US Food and Drug Administration (FDA) related to this option to selectively maintain patients on study therapy after evidence of PD is demonstrated, this amendment incorporates specific criteria described below (see Section 7.5). Criteria for removing patients from the study include the following:

1) Patients with unequivocal evidence of any new metastasis including development of peritoneal studding or malignant ascites must be removed from study.
2) Patients whose disease progression is such that they are at risk for catastrophic complications from vital organ compression (eg, spinal cord compression, small bowel obstruction, encroachment of major blood vessels, etc) must be removed from study immediately and treated appropriately.
3) Patients with evidence of progression based on RECIST must be removed from study if the subsequent assessment (8 weeks later) demonstrates evidence of progressive disease.
4) Patients must be informed of the evidence of progression and the availability of alternative treatments.

In addition, patients who have disease progression that is symptomatic or leads to altered organ function, such as increases in bilirubin by 1 CTC severity grade that could represent altered drug clearance, should be removed from the study.

If a patient is maintained on study treatment after evidence for PD, serial objective monitoring will continue according to the Schedules of Events. Statistical analysis of clinical outcomes (eg, time to progression) will continue to employ the predefined definitions for PD (RECIST or GCIG CA-125, Section 15.4) even if the patient continues protocol treatment after PD. In this setting, the date and criteria for the original determination of PD per protocol will be recorded in the CRF. The date and criteria for subsequent treatment discontinuation will be recorded in the CRF in addition to dosing history and serial evaluations of disease, including CA-125 and imaging results.

Purposes for Amendment 3

- To clarify that patients may be maintained on study treatment after PD in selected circumstances, and to define the criteria for withdrawing patients from study treatment.
- To correct typographical errors, punctuation, grammar, and formatting.

For specific changes included in Amendment 2 which are maintained in this Amendment 3, see Section 15.8. For specific examples of changes in text and where the changes are located, see Section 15.9.

PROTOCOL SUMMARY

Study Title: Randomized Phase 2 Study of MLN8237, an Aurora A Kinase Inhibitor, Plus Weekly Paclitaxel or Weekly Paclitaxel Alone in Patients with Recurrent Epithelial Ovarian, Fallopian Tube, or Primary Peritoneal Cancer, Preceded by a Phase 1 Portion in Patients with Ovarian or Breast Cancer

Principal Investigator: [redacted]

Study Phase: 1/2

Number of Patients: Approximately 172 total patients are planned for enrollment, including approximately 36 patients in the Phase 1 portion and 136 patients in the randomized, Phase 2 portion

Study Objectives:

Phase 1 Objectives

The primary objectives of Phase 1 are as follows:

- To assess the safety and tolerability of MLN8237 plus weekly paclitaxel
- To determine the recommended dose and schedule of MLN8237 and dose of paclitaxel to be used as the combination treatment arm in Phase 2

The secondary objectives of Phase 1 are as follows:

- To characterize the effect of concomitant administration of MLN8237 on the pharmacokinetics (PK) of paclitaxel
- To characterize the PK of MLN8237 administered concomitantly with weekly paclitaxel
- To assess the best overall combined response in patients with recurrent ovarian cancer (OC) or breast cancer

The exploratory objective of Phase 1 is as follows:

- [redacted]
- [redacted]

Phase 2 Objectives

The primary objective of Phase 2 is as follows:

- To assess progression-free survival (PFS) after treatment with the combination of MLN8237 and weekly paclitaxel or weekly paclitaxel alone in patients with recurrent OC, and to compare PFS of the populations enrolled to these 2 treatment arms

The secondary objectives of Phase 2 are as follows:

- To estimate the overall response rate (ORR), duration of response (DOR), time to disease progression (TTP), and overall survival (OS) associated with MLN8237 plus weekly paclitaxel and weekly paclitaxel alone in patients with recurrent OC
- To assess the safety and tolerability of MLN8237 plus weekly paclitaxel

The exploratory objectives of Phase 2 are as follows:

- [redacted]

[redacted]

- [redacted]
- [redacted]

Overview of Study Design: This is an open-label, multicenter study with a nonrandomized Phase 1 portion and an open-label, randomized, Phase 2 portion evaluating MLN8237 in combination with weekly paclitaxel in adult female patients with advanced breast cancer (Phase 1 portion only) and recurrent OC (both Phase 1 and Phase 2 portions). During the lead-in Phase 1 portion of the study, the MTD of MLN8237 combined with paclitaxel will be determined and the PK characterized in eligible patients. The Phase 1 portion will include female patients with adenocarcinoma of the breast or patients with recurrent OC. Recurrent disease must be documented within 12 months after discontinuation of platinum therapy. Patients with platinum-refractory disease, defined by progression during primary or subsequent platinum-based therapy or persistent radiographic disease after primary or subsequent platinum-based therapy, will be included. When a recommended Phase 2 dose (RP2D) is determined in the Phase 1 portion of the study, patients with recurrent OC who have a treatment-free interval ≤ 12 months after platinum therapy or patients who experience persistent or recurrent disease during platinum therapy and who are candidates for treatment with weekly paclitaxel will be enrolled in the Phase 2 portion. These latter patients will be randomized to study treatment with either the combination of MLN8237 plus weekly paclitaxel or single-agent weekly paclitaxel in the Phase 2 portion.

The RP2D of MLN8237 combined with weekly paclitaxel will be determined in Phase 1 for further evaluation in the Phase 2 portion of the study based on composite clinical data, including PK, safety, and tolerability over multiple treatment cycles, described in Section 4.1. The RP2D will be determined upon review of available data by the sponsor, a Safety Monitoring Committee (SMC), and consultation with the Phase 1 investigators. This review to determine the RP2D will be summarized, along with available supporting data from the Phase 1 part of the study, and provided to the Phase 2 investigators prior to initiation of enrollment to that part of the study.

An interim analysis will be performed after the first 72 evaluable patients in the Phase 2 portion have either completed a minimum of 2 cycles of therapy or have discontinued study treatment. The interim analysis will be based on the clinical benefit rate (CBR), which includes the best overall combined response and stable disease (SD), and a patient must have at least 2 continuous assessments of SD to be counted. The study will be terminated if the CBR of the MLN8237 plus weekly paclitaxel arm is at least 10% lower than that of the weekly paclitaxel-alone arm.

The final analysis will be performed after a total of 110 progession-free survival (PFS) events (progressive disease [PD] or death) have occurred in Phase 2, and PFS between the 2 treatment arms will be compared.

Patients cannot have received antineoplastic therapy or radiotherapy within the 3 weeks before enrollment (2 weeks for regimens with recovery expected within 7 to 14 days).

Patients with recurrent OC must have PD per RECIST, version 1.1, or modified Gynecologic Cancer Intergroup (GCIG) CA-125 criteria and have experienced recurrence ≤ 12 months after discontinuation of a platinum-based chemotherapy regimen. Patients with CA-125 marker-only evidence for PD are included only if there is also radiological evidence of disease. In the Phase 1 portion, patients with breast cancer must have measurable disease.

During the Phase 1 portion of the study, the starting dosage of MLN8237 will be 10 mg orally (PO) twice daily (BID) for 3 days followed by a 4-day rest period (3 days on/4 days off) repeated weekly for 3 weeks in a 28-day cycle. In new cohorts, dose escalation for the Phase 1 study may proceed until the maximum tolerated dose (MTD) is determined. The dose of MLN8237 will be doubled in the next 2 successive cohorts if first-cycle dose-limiting toxicity (DLT) is not observed; after reaching 40 mg BID or if 1 patient experiences DLT in earlier cohorts, subsequent increases in MLN8237 dose will be approximately 25%. Doses at each level will be adjusted to accommodate the available tablet strengths. Doses can be reduced in subsequent treatment cycles as described in the dose modification section of the protocol.

One of the objectives of the Phase 1 portion of this study is to evaluate the effect of concomitant administration of MLN8237 on paclitaxel PK. Therefore, MLN8237 will be administered beginning on Day 8 in Cycle 2 in order that paclitaxel PK following the Day 1 dose can be evaluated without concomitantly administered MLN8237 to serve as reference for comparison to paclitaxel PK, evaluated at Cycle 1, Day 1 in the presence of MLN8237. For Cycle 2 only, during the Phase 1 portion, patients will receive 12 doses of MLN8237, administered BID on Days 8, 9, 10, 15, 16, and 17.

During the Phase 2 portion, the MLN8237 plus weekly paclitaxel regimen will be determined by the Phase 1 portion; patients will be assigned randomly in a 1:1 ratio to treatment with MLN8237 plus weekly paclitaxel or weekly paclitaxel alone. Paclitaxel will be administered as an intravenous (IV) infusion on Days 1, 8, and 15 of each 28-day cycle. Patients randomized to treatment with single-agent paclitaxel will receive a planned starting dose of 80 mg/m^2, which may be reduced or delayed if required to manage toxicities. The planned combination therapy schedule includes 9 days (18 doses) of MLN8237 drug administration overlapping weekly paclitaxel administration (Days 1, 8, and 15) in each 28-day cycle.

Individual dose reductions will be made on the basis of the adverse events (AEs) observed as defined by protocol. Patients may continue to receive repeated cycles of treatment as part of this protocol for up to 24 months, until there is evidence of PD or unacceptable treatment-related toxicity, or until another antineoplastic therapy is started. Treatment with MLN8237 and paclitaxel may be continued beyond 24 months if after discussion between the investigator and the sponsor it is determined that a patient would clearly derive benefit from continued therapy. Patients who discontinue their participation in the study for any reason may continue paclitaxel or other taxane therapy at the discretion of the treating physician. In Phase 1, the strategy for de-escalation or evaluation of a reduced MLN8237 dosing schedule, if required, will be determined by the sponsor and investigators upon review of PK and clinical data from the initial cohorts.

The Phase 1 dose escalation cohorts include the option to reduce the dose of paclitaxel (eg, from a starting dose of 80 mg/m^2 reduced to 70 or 60 mg/m^2) if elevated paclitaxel exposures or excess toxicities are observed when administered in combination with MLN8237. If a combination regimen is investigated in the Phase 1 portion with a starting dose of paclitaxel below 80 mg/m^2 and the individual patient tolerates the starting doses without severe (CTC Grade 3 or higher) toxicity, the paclitaxel dose can be escalated after completion of Cycle 2 up to a maximum of 80 mg/m^2 and safety monitoring will continue as required by the Schedule of Events. Upon determination of the recommended Phase 2 dose (RP2D) regimen to be used in the Phase 2 portion, the sponsor and Phase 1 investigators will review safety and tolerability of patients who received paclitaxel escalated from a reduced starting dose up to 80 mg/m^2; if the Phase 1 data indicate that such paclitaxel escalation was generally tolerable, this option will be continued in Phase 2 for individual patients enrolled to the combination arm. After a paclitaxel dose escalation, management of toxicities by dose reduction, delays, or supportive care continue to follow protocol guidelines.

Extent of disease evaluations, based on radiographic procedures, such as computed tomography (CT) with IV contrast or magnetic resonance imaging (MRI) scans for sites of disease not adequately imaged by CT, nuclear medicine scans as appropriate (eg, known or suspected bone metastases), and findings on physical examination, will be obtained at the end of every second treatment cycle (ie, approximately every 8 weeks, within the week prior to the start of a new cycle). Scans are required at the End of Treatment (EOT)/End of Study (EOS) visit only if PD has not been documented previously or it has been 8 weeks or longer since the previous evaluation. Patients (enrolled in both Phase 1 and Phase 2) followed off treatment will be evaluated every 8 weeks until the occurrence of 110 PFS events (PD or death) in Phase 2. CA-125 levels will be obtained according to standard of care within 9 days of screening and sufficient time after prior therapy, at the end of every treatment cycle, within the week prior to the start of a new cycle, at the EOT visit, and during follow-up visits for patients who are followed off treatment. Confirmation of response by CA-125 levels must be obtained 28 days after the previous sample.<br><br>Response will be based on either Response Evaluation Criteria in Solid Tumors (RECIST), version 1.1, for measurable neoplastic disease or on changes in CA-125 based on the CA-125 response definition for patients with CA-125 levels in excess of 70 units/mL according to modified GCIG response criteria. PD will be based on RECIST, version 1.1, for measurable neoplastic disease or on CA-125 criteria with elevated (> 70 units/mL) levels on 2 occasions and other criteria as described in the modified GCIG criteria.<br><br>Toxicity will be evaluated according to National Cancer Institute Common Terminology Criteria for Adverse Events (NCI CTCAE), version 4.02, effective as of 01 October 2009.<br><br>AEs will be assessed, and laboratory values, vital signs, and electrocardiograms will be obtained to evaluate the safety and tolerability of the combination of MLN8237 plus weekly paclitaxel.
**Study Population:** Patients must be female and 18 years of age or older. The Phase 1 portion will include female patients with adenocarcinoma of the breast or patients with recurrent OC who have a treatment-free interval ≤ 12 months after platinum therapy or patients who experience persistent or recurrent disease during platinum therapy. Patients with persistent OC may enroll under conditions described in the Eligibility Criteria. Patients with breast cancer must have measurable disease. The Phase 2 portion will include only patients with OC who have a treatment-free interval ≤ 12 months after platinum therapy or patients who experience persistent or recurrent disease during platinum therapy and who are candidates for treatment with weekly paclitaxel. Prior treatments must have included a platinum-based chemotherapy and a taxane. In patients previously treated with weekly taxane, recurrent disease must not have occurred during or within 3 months of this treatment. Patients cannot have received antineoplastic therapy or radiotherapy within the 3 weeks before enrollment (2 weeks for regimens with recovery expected within 7 to 14 days). Patients with recurrent OC must have PD per RECIST, version 1.1, or modified GCIG CA-125 criteria and have experienced recurrence ≤ 12 months after discontinuation of a platinum-based chemotherapy regimen. Patients with CA-125 marker evidence for PD are included only if there is also clinical evidence of PD. Excluded are patients who received previous treatment with an Aurora A-targeted agent (including MLN8237); those who were treated with any investigational products within 21 days before the first dose of study drug; and those who received treatment with more than 4 cytotoxic treatment regimens in the metastatic setting.
**Number of Study Center(s):** Approximately 3 study centers in the Phase 1 portion and approximately 40 study centers in the Phase 2 portion
**Duration of Study:** The estimated duration of Phase 1 portion is approximately 34 months, and

the estimated duration of Phase 2 portion is approximately 36 months.

Study Flow Diagrams

Phase 1 Dose Escalation

Eligible patients with:

a) epithelial ovarian, fallopian tube, or primary peritoneal adenocarcinoma and recurrence 0 to 12 months after prior platinum therapy; or

b) adenocarcinoma of the breast

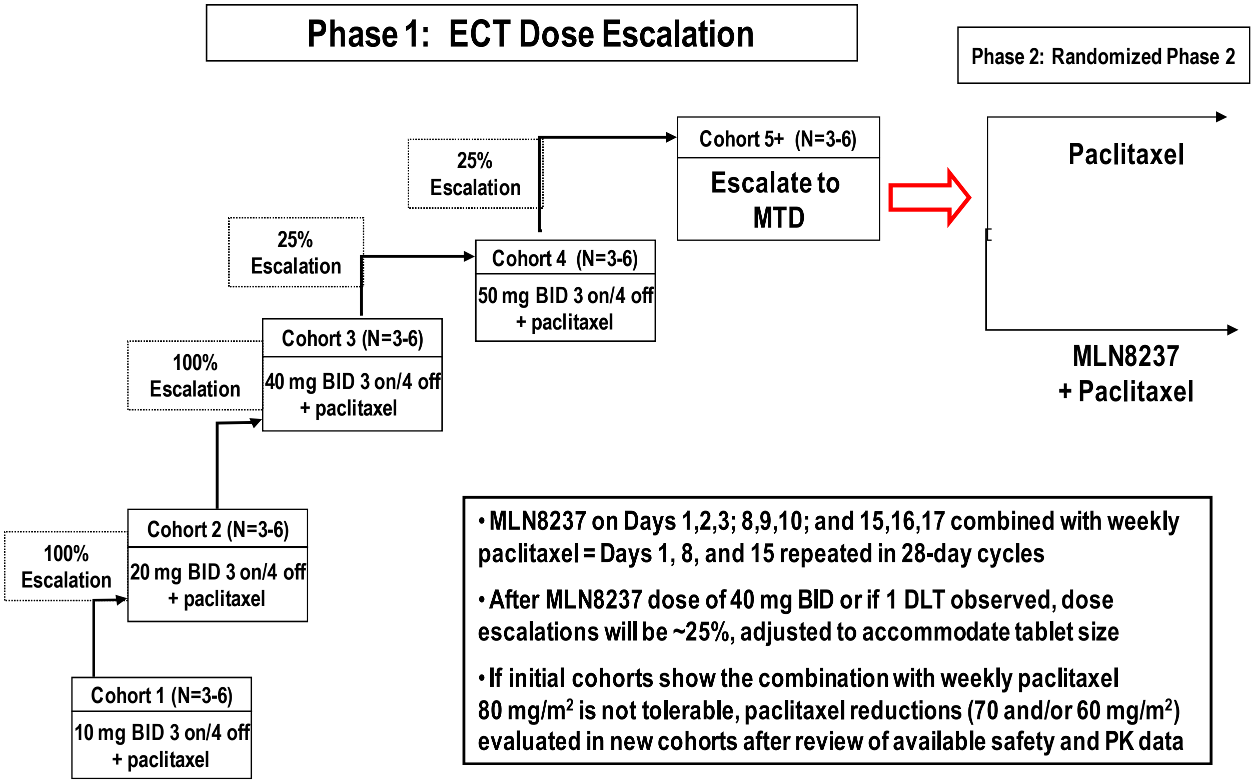

Abbreviations: BID = twice a day; DLT = dose-limiting toxicity; ECT = enteric-coated tablet; MTD = maximum tolerated dose; PK = pharmacokinetic

Notes: Enrollment will expand to at least 12 patients at the recommended Phase 2 dose (RP2D), to be determined for further evaluation in the Phase 2 portion of the study based on composite clinical data, including PK, safety, and tolerability over multiple treatment cycles. The RP2D will be determined upon review of the available clinical data by the sponsor, a Safety Monitoring Committee, and consultation with the Phase 1 investigators.

An intermediate dose reduction in paclitaxel dose to 70 mg/m^2 or 60 mg/m^2 may be considered upon agreement by the sponsor and review of pharmacokinetic results, including the observed increases in paclitaxel exposure when combined with MLN8237. If 60-mg/m^2 or 70-mg/m^2 paclitaxel are well tolerated when given in combination with MLN8237, re-escalation to 80-mg/m^2 paclitaxel may be considered.

Phase 2, Two-Stage Design Schema

Eligible patients with epithelial ovarian, fallopian tube, or primary peritoneal adenocarcinoma, recurrent disease 0 to 12 months after platinum therapy

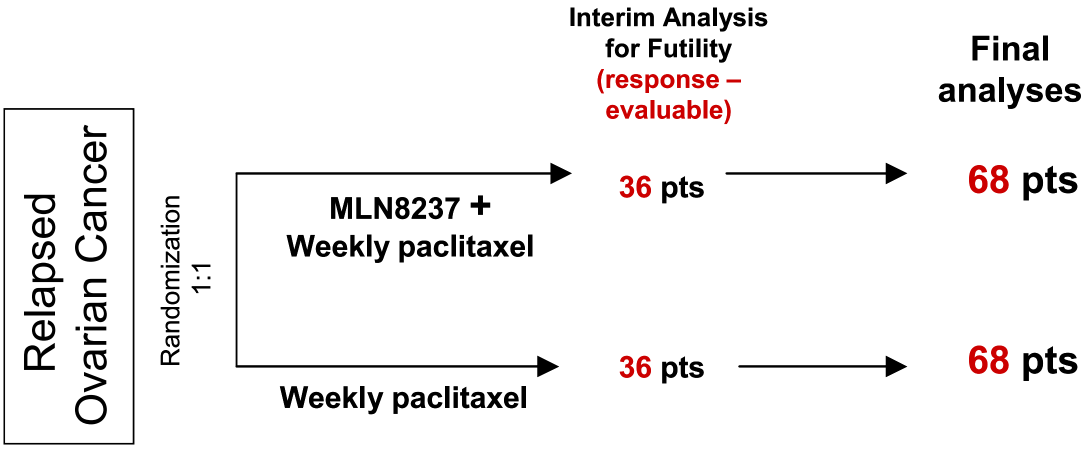

Abbreviations: pts = patients.

Recurrent Ovarian Cancer = treatment-free interval ≤ 12 months after platinum therapy or relapse during platinum therapy.

The Recommended Phase 2 Dose of MLN8237 plus weekly paclitaxel will be identified during Phase 1.

Schedules of Events

Phase 1 – Patients With Epithelial Ovarian, Fallopian Tube, or Primary Peritoneal Cancer; or Breast Cancer																	
		**Cycle 1 (28-Day Cycle)**								**Subsequent 28-Day Cycles**						**EOT[d]**	**EOS[d]/FU[e]**
		**Day**								**Day**							
**Procedure**	**Screen[a]**	**1[b]**	**2**	**3**	**8**	**9**	**10**	**15**	**21**	**1**	**2**	**3**	**8**	**15**	**21[c]**		
Informed Consent[f]	X																
Inclusion/Exclusion[a,g]	X																
Demographics	X																
Complete Medical History[h]	X																
Complete Physical Examination, with Neurological Examination	X																
Symptom-Directed Physical Examination[i]		X[b]			X			X	X	X			X	X	X[c]	X	X[i]
Vital Signs[j]	X	X[b]			X			X	X	X			X	X	X[c]	X	X[j]
Height	X																
Weight[k]	X	X[b]								X						X	X[k]
ECOG PS[l]	X	X[b]								X						X	X[l]
Electrocardiogram[m]	X	X[b]															
Hematology[n]	X	X[b]			X			X	X	X			X	X	X[c]	X	
Serum Chemistry and TSH[o]	X	X[b]								X						X	
Urinalysis[p]	X	X[b]														X	
Serum Pregnancy Test[q]	X	X[q]															
CA-125[r]	X	X[r]							X[r]						X[r]	X	X
Extent of Disease Evaluation by RECIST, version 1.1[s]	X														X[s]	X	X[s]
Banked Tumor Tissue[t]	X																
Blood for Genotyping[u]		X															

**Phase 1 – Patients With Epithelial Ovarian, Fallopian Tube, or Primary Peritoneal Cancer; or Breast Cancer**																	
		**Cycle 1 (28-Day Cycle)**								**Subsequent 28-Day Cycles**						**EOT[d]**	**EOS[d]/FU[e]**
		**Day**								**Day**							
**Procedure**	**Screen[a]**	**1[b]**	**2**	**3**	**8**	**9**	**10**	**15**	**21**	**1**	**2**	**3**	**8**	**15**	**21[c]**		
Concomitant Medications and Procedures	Recorded from 28 days prior to first dose of study drug through 30 days after the last dose of study drug or until the start of subsequent antineoplastic therapy, whichever occurs first																
Adverse Event Reporting		Recorded from first dose of study drug through 30 days after the last dose of study drug or until the start of subsequent antineoplastic therapy, whichever occurs first															
	**Serious adverse events** will be reported from signing of the informed consent form through 30 days after the last dose of study drug (see Section 7.4.18).[v]																
Paclitaxel PK Samples[w]		X	X	X						In Cycle 2 only							
MLN8237 PK Samples[x]		X		X													
Administration of Weekly Paclitaxel[y]		X			X			X		X			X	X			
Administration of MLN8237[z]		C1D1–C1D3; C1D8–C1D10; C1D15–C1D17											C2D8–C2D10; C2D15-C2D17<br>Cycle 3 and beyond: same as Cycle 1 dosing				
Patient Diary Review[aa]					X			X	X	X[aa]			X	X	X[aa]	X	

Abbreviations: β-hCG = beta-human chrionic gonadotropin; CXDY = Cycle X, Day Y; D = day; ECOG PS = Eastern Cooperative Oncology Group Performance Status; EOS = End of Study visit; EOT = End of Treatment visit; FU = Follow-up; h = hour(s); min = minute(s); PD = progressive disease; PFS = progression-free survival; PK = pharmacokinetics; RECIST = Response Evaluation Criteria in Solid Tumors; TSH = thyroid-stimulating hormone

Note: Each treatment cycle is 28 days in length. Tests and procedures should be done on schedule, but visit windows of ± 2 days are allowed (except as otherwise specified) occasionally for holidays, vacations, and other administrative reasons. **If extenuating circumstances prevent a patient from beginning treatment or completing a scheduled assessment within this time frame, the patient may continue in the study only with written permission of the medical monitor.**

a Within 28 days before the Cycle 1, Day 1 dose of study drug, unless otherwise specified

b Cycle 1, Day 1 is baseline. Cycle 1, Day 1 evaluations and procedures are to be performed within the 4 days prior to the first dose of any study drug, unless otherwise specified. A repeat of the procedure is not required on Cycle 1, Day 1 if screening procedures were performed within 4 days of the Cycle 1, Day 1 dose of study drug. The exception is the CA-125, as 2 CA-125 values are required to be collected prior to dosing at Cycle 1, Day 1

c In patients who tolerate study treatment through multiple cycles, the Day 21 evaluation can be done by telephone contact, with or without laboratory testing, at the discretion of the investigator, if in the prior 2 cycles the patient tolerated study treatment (ie, without the requirement for dose reduction or without ≥ Grade 3 treatment-related toxicity evaluated according to National Cancer Institute Common Terminology Criteria for Adverse Events [NCI CTCAE],

Phase 1 – Patients With Epithelial Ovarian, Fallopian Tube, or Primary Peritoneal Cancer; or Breast Cancer																
		Cycle 1 (28-Day Cycle)								Subsequent 28-Day Cycles							
		Day								Day						EOT[d]	EOS[d]/FU[e]
Procedure	Screen[a]	1[b]	2	3	8	9	10	15	21	1	2	3	8	15	21[c]		

version 4.02).

d For all patients, the EOT visit will be conducted 30 (+ 10) days after the last dose of study drug for all patients. Patients who enter the Follow-Up period (eg, patients who discontinue study drug treatment before the occurrence of progressive disease [PD]/death) will be followed off treatment and will complete an EOT visit and later complete an EOS visit. The EOS visit will be completed at the time the patient completes or withdraws from the Follow-Up period. Note: Patients who do not enter the Follow-Up period (eg, experience PD, death during study drug treatment) will complete the EOT visit only.

e Patients who do not experience PD will be followed off treatment once every 8 weeks until the occurrence of 110 progression-free survival (PFS) events (PD or death, whichever occurs first) in Phase 2 are documented for this study. Patients may withdraw from the Follow-Up period before the occurrence of 110 PFS events in Phase 2 for any of the following reasons: PD, withdrawal of informed consent, starting a different anticancer therapy, or lost to follow-up. The EOS visit will be completed at the time the patient completes or withdraws from the Follow-Up period. Patients who do not enter the Follow-Up period (eg, experience PD or die during active study treatment) will complete the EOT visit only.

f Informed consent may be obtained earlier and must be obtained before performance of any study-specific procedures.

g The cohort that defines the RP2D will include a minimum of 6 patients, at least 4 with recurrent OC and at least 3 who tolerate the first 2 cycles without a requirement for myeloid growth factor support. (Six additional patients will be enrolled in the expansion cohort to refine understanding of safety and PK, for a total of at least 12 patients.)

h Including history of prior treatment for ovarian cancer and breast cancer and recurrence dates of ovarian cancer.

i The Cycle 1, Day 1 (baseline) symptom-directed physical examination is not required if the screening physical examination was conducted within the 4 days prior to administration of the Cycle 1, Day 1 dose of study drug. A symptom-directed physical examination will be repeated within 3 days before the beginning (Day 1) of each new treatment cycle; on Day 8 (± 2 days), Day 15 (± 2 days), and Day 21 (± 2 days) of each treatment cycle; at the EOT visit; and at the EOS visit (for patients who are followed off treatment and complete or withdraw from the Follow-Up period).

j Vital signs (blood pressure, heart rate, and oral temperature) measurements will be obtained during screening; at Cycle 1, Day 1 (baseline); within 3 days before the beginning (Day 1) of each treatment cycle; on Day 8 (± 2 days), Day 15 (± 2 days), and Day 21 (± 2 days); at the EOT visit; and at the EOS visit (for patients who are followed off treatment and complete or withdraw from the Follow-Up period). Blood pressure should be determined with the patient in a seated position after the patient has been sitting quietly for 5 minutes. If the screening assessment was done within 4 days prior to Cycle 1, Day 1, an assessment at Cycle 1, Day 1 is not necessary.

k Weight will be measured at screening; at Cycle 1, Day 1 (baseline); within 3 days before the beginning (Day 1) of each treatment cycle; at the EOT visit; and at the EOS visit (for patients who are followed off treatment and complete or withdraw from the Follow-Up period). If the screening assessment was done within 4 days prior to Cycle 1, Day 1, an assessment at Cycle 1, Day 1 is not necessary.

l ECOG performance status will be assessed during screening; at Cycle 1, Day 1 (baseline); within 3 days before the beginning (Day 1) of each treatment cycle; at the EOT visit; and at the EOS visit (for patients who are followed off treatment and complete or withdraw from the Follow-Up period). Refer to Section 15.1. If the screening assessment was done within 4 days prior to Cycle 1, Day 1, an assessment at Cycle 1, Day 1 is not necessary.

m A 12-lead electrocardiogram (ECG) will be obtained at screening and at Cycle 1, Day 1 (baseline). If the screening ECG was obtained within the 4 days prior to the Cycle 1, Day 1 dose of study drug, a repeat ECG at Cycle 1, Day 1 is not necessary. Additional ECGs may be obtained if clinically indicated. ECG

Phase 1 – Patients With Epithelial Ovarian, Fallopian Tube, or Primary Peritoneal Cancer; or Breast Cancer																
		Cycle 1 (28-Day Cycle)								Subsequent 28-Day Cycles						EOT[d]	EOS[d]/FU[e]
		Day								Day							
Procedure	Screen[a]	1[b]	2	3	8	9	10	15	21	1	2	3	8	15	21[c]		

assessments are to be performed with the patient supine and rested for 5 minutes and before any closely timed blood collection.

n A blood sample for hematology will be obtained during screening; at Cycle 1, Day 1 (baseline); within 3 days before the beginning (Day 1) of each treatment cycle; on Day 8 (± 2 days), Day 15 (± 2 days), and Day 21 (± 2 days); and at the EOT visit. In patients who tolerate study treatment through multiple cycles, the Day 21 clinical laboratory assessment can become optional, at the discretion of the investigator, if in the prior 2 cycles the patient tolerated study treatment (ie, without the requirement for dose reduction or without ≥ Grade 3 treatment-related toxicity evaluated according to NCI CTCAE, version 4.02). Hematology includes hemoglobin, hematocrit, white blood cell (WBC) count with differential count (machine results acceptable), and platelet count. If screening values were obtained within the 4 days prior to Cycle 1, Day 1, repeat hematology testing at Cycle 1, Day 1 is not necessary. If a patient has an absolute neutrophil count (ANC) less than 500/mm^3 or a platelet count less than 25,000/mm^3, or both, the complete blood count (CBC) with differential should be repeated at least every 2 to 3 days until the ANC or platelet count (or both, if both were decreased) have exceeded these values on at least 2 occasions.

o A blood sample for serum chemistries will be obtained during screening; at Cycle 1, Day 1 (baseline); within 3 days before the beginning (Day 1) of each treatment cycle; and at the EOT visit. Serum chemistries include sodium, potassium, blood urea nitrogen (BUN), creatinine, alkaline phosphatase, AST (SGOT), ALT (SGPT), total bilirubin, carbon dioxide, albumin, calcium, chloride, phosphate, magnesium, and glucose. Thyroid stimulating hormone (TSH) level will be measured at Cycle 1, Day 1 (baseline) and at the EOT visit; this test should be repeated during the treatment period as clinically indicated. If screening values were obtained and acceptable within the 4 days prior to Cycle 1, Day 1, repeat clinical chemistry testing at Cycle 1, Day 1 is not necessary.

p A urine sample for urinalysis will be obtained during screening, at Cycle 1, Day 1 (baseline), and at the EOT visit. Urinalysis includes testing for protein and blood. If urine protein by dipstick changes to 3+ (or 2+ that is reconfirmed at least 1 day later), then a 24-hour urine collection should be done for protein and creatinine clearance. The screening or Cycle 1, Day 1 (baseline) urinalysis should include microscopic examination of the sediment. If screening values were obtained and acceptable within the 4 days prior to the Cycle 1, Day 1 dose of study drug, a repeat urinalysis at Cycle 1, Day 1 is not necessary.

q A serum β-hCG pregnancy test will be performed only for patients of childbearing potential during screening and again at Cycle 1, Day 1 (baseline) if the screening test was performed more than 4 days before the first dose of any study drug. The results must be negative within 4 days before the first dose of any study drug (MLN8237 or paclitaxel) is administered (ie, within the 4 days prior to Cycle 1, Day 1), or as otherwise required by local regulations. Additional pregnancy testing may be performed during the study at the discretion of the investigator, as per request of IEC/IRB, or if required by local regulations.

r For patients with ovarian, fallopian tube, or peritoneal cancer, CA-125 levels will be obtained according to standard of care within 9 days of screening and sufficient time after prior therapy; within 4 days prior to the dose at Cycle 1, Day 1; at the end of every treatment cycle, within the week prior to the start of a new cycle; at the EOT visit; and at the EOS visit (for patients who are followed off treatment and complete or withdraw from the Follow-Up period) (see Section 15.4). The investigator's determination of response by CA-125 is required at the end of every 2 cycles, designed to coincide with imaging studies for evaluation of response by RECIST criteria, if applicable.Two CA-125 levels must be obtained prior to dosing on Cycle 1, Day 1. CA-125 should be evaluated to determine if PD is substantiated prior to the start of each new cycle. Patients who discontinue study drug treatment before the occurrence of PD will be assessed at the EOT visit and at follow-up visits once every 8 weeks until the occurrence of 110 PFS events (PD or death, whichever occurs first) in Phase 2. It is noted that confirmation of response by CA-125 levels must be obtained at least 28 days after the previous sample. Response evaluation employs modified Gynecologic Cancer Intergroup (GCIG) criteria for changes in CA-125.

s Extent of disease evaluation by the Response Evaluation Criteria in Solid Tumors (RECIST), version 1.1, includes computed tomography (CT) with IV

Phase 1 – Patients With Epithelial Ovarian, Fallopian Tube, or Primary Peritoneal Cancer; or Breast Cancer																	
		Cycle 1 (28-Day Cycle)								Subsequent 28-Day Cycles						EOT[d]	EOS[d]/FU[e]
		Day								Day							
Procedure	Screen[a]	1[b]	2	3	8	9	10	15	21	1	2	3	8	15	21[c]		

contrast of the abdomen and pelvis, as well as other anatomical areas as appropriate based on the known prior extent of the patient's disease and clinical symptoms (see Section 15.3). Magnetic resonance imaging (MRI) scans may be used to assess sites of disease not adequately imaged by CT. If the patient has had appropriate scans performed within 28 days before the Cycle 1, Day 1 dose of MLN8237, then those scans may be used for tumor lesion measurement during screening. Repeat CT (with IV contrast) or MRI scans, or both, are to be performed at the completion of Cycle 2 and at the completion of every 2 cycles (approximately every 8 weeks, between Days 21 and 28 of every even cycle) thereafter for up to 24 months or until PD is documented (except as permitted per agreement with the Investigator and Medical Monitor-Section 7.5). The same imaging modality should be used throughout the study for each site of disease. For responders, radiographic images will be read locally but will be collected and provided to the sponsor for subsequent review. Scans are required at the EOT visit only if PD has not been documented previously and it has been 8 weeks or more since the previous evaluation. Other procedures, such as physical examinations and other scintigraphic examinations (eg, bone scans for patients with known or suspected bone metastases), also should be taken into consideration when evaluating the extent of malignant disease. Patients who discontinue study drug treatment before occurrence of PD will be assessed at EOT and follow-up visits once every 8 weeks until the occurrence of 110 PFS events (PD or death, whichever occurs first) in Phase 2.

t Banked tumor tissue (submitted paraffin-embedded block if available, otherwise slides) will be obtained from a previous resection or biopsy that was done as part of the patient's standard care. [redacted].

u One blood sample will be obtained at Cycle 1, Day 1 before administration of the first dose of study drug (may be obtained within the 4 days prior to Cycle 1, Day 1) [redacted]

v Only those serious events that occur after the first dose of any study drug will be **entered** in the eCRF, **although all serious adverse events and serious pretreatment events must be reported to Millennium Pharmacovigilance or designee**.

w Blood samples to measure plasma concentrations of paclitaxel will be collected at the following time points on Day 1 of both Cycle 1 and Cycle 2: within 1 h before the start of the paclitaxel infusion; at the end of the paclitaxel infusion (immediately before switching off the infusion pump); at 5 (± 1) min, 15 (± 3) min, 0.5 h (± 5 min), 1 h (± 10 min), 2 h (± 20 min), 3 h (± 20 min), 7 h (± 30 min), 10 h (± 30 min), 23 (± 2) h, and 47 (± 4) h after completion of the paclitaxel infusion. The 23- and 47-h blood samples will be collected prior to MLN8237 dosing. (See Section 7.4.19 and Section 15.6)

x Blood samples to measure plasma concentrations of MLN8237 will be collected at the following time points on Day 1 and Day 3 in Cycle 1: immediately before the morning dose of MLN8237, and at 1 h (± 10 min), 2 h (± 20 min), 3 h (± 20 min), 4 h (± 20 min), 5 h (± 20 min), 9 h (± 30 min), and 12 (± 1) h after the MLN8237 morning dose on Days 1 and 3. (See Section 7.4.19 and Section 15.6)

y Paclitaxel will be administered as an intravenous (IV) infusion on Days 1, 8, and 15 of each 28-day treatment cycle. Premedication may be administered in accordance with the recommendations described in the product label. Modifications to paclitaxel infusion are allowed upon agreement by the medical monitor and will be documented in the electronic case report form (eCRF). If the paclitaxel starting dose is less than 80 mg/m^2 during the Phase 1 study, then dose escalation is allowed after Cycle 2 up to a maximum of 80 mg/m^2 given lack of significant toxicites in early cycles using a reduced dose of paclitaxel. Upon review of dose escalation experience from Phase 1 by the sponsor and Phase 1 investigators, the option for paclitaxel escalation will be permitted in Phase 2 for individual patients who do not experience significant (eg, CTC Grade 3) toxicities in early cycle(s).

Phase 1 – Patients With Epithelial Ovarian, Fallopian Tube, or Primary Peritoneal Cancer; or Breast Cancer																
		Cycle 1 (28-Day Cycle)								Subsequent 28-Day Cycles						
		Day								Day						EOT[d]	EOS[d]/FU[e]
Procedure	Screen[a]	1[b]	2	3	8	9	10	15	21	1	2	3	8	15	21[c]		

z During Cycle 1, and Cycle 3 and beyond, MLN8237 dosing will begin on Day 1; subsequent dosing will occur on Days 2, 3, 8, 9, 10, 15. 16, and 17. During **Cycle 2 only**, MLN8237 dosing will begin on Day 8 and will continue on Days 9 and 10, and on Days 15, 16, and 17. Refer to Table 6-1 for MLN8237 dose administration and titration schema. On days when both paclitaxel and MLN8237 are administered, MLN8237 should be administered 1 hour before the start of the paclitaxel infusion. MLN8237 will be dispensed to the patient on Day 1.

aa The study center staff will check the patient diary versus the patient's supply of MLN8237 tablets on Day 8 (for dosing on Days 1 to 3), on Day 15 (for dosing on Days 8 to 10), and on Day 21 (for dosing on Days 15 to 17), as applicable, of each treatment cycle and at the EOT visit to ensure compliance. For patients who do not require a clinic visit on Day 21, the medication count may be confirmed verbally with the patient by telephone at this visit; however, a check of the patient diary versus the patient's supply of MLN8237 tablets should be performed at the next visit (eg, on Day 1 of the subsequent cycle or at the EOT visit, if applicable).

Phase 2 – Patients With Recurrent Ovarian, Fallopian Tube, or Primary Peritoneal Cancer Randomized to MLN8237 Plus Weekly Paclitaxel or Weekly Paclitaxel Alone

		Cycle 1 (28-Day Cycle)				Subsequent 28-Day Cycles					
		Day				Day				EOT[d]	EOS[d]/FU[e]
**Procedure**	**Screen[a]**	**1[b]**	**8**	**15**	**21**	**1**	**8**	**15**	**21[c]**		
Informed Consent[f]	X										
Inclusion/Exclusion[a]	X										
Demographics	X										
Complete Medical History[g]	X										
Complete Physical Examination, with Neurological Examination	X										
Symptom-Directed Physical Examination[h]		X[b]	X	X	X	X	X	X	X[c]	X	X[h]
Vital Signs[i]	X	X[b]	X	X	X	X	X	X	X[c]	X	X[i]
Height	X										
Weight[j]	X	X[b]				X				X	X[j]
ECOG PS[k]	X	X[b]				X				X	X[k]
Electrocardiogram[l]	X	X[b]									
Hematology[m]	X	X[b]	X	X	X	X	X	X	X[c]	X	
Serum Chemistry and TSH[n]	X	X[b]				X				X	
Urinalysis[o]	X	X[b]								X	
Serum Pregnancy Test[p]	X	X[p]									
CA-125[q]	X	X[q]			X[q]				X[q]	X	X
Extent of Disease Evaluation by RECIST, version 1.1[r]	X								X[r]	X	X[r]
Banked Tumor Tissue[s]	X										
Blood for Genotyping[t]		X									
Blood for Serum Markers of Response[u]		X		X			X[u]				

Phase 2 – Patients With Recurrent Ovarian, Fallopian Tube, or Primary Peritoneal Cancer Randomized to MLN8237 Plus Weekly Paclitaxel or Weekly Paclitaxel Alone

		Cycle 1 (28-Day Cycle)				Subsequent 28-Day Cycles					
		Day				Day				EOT[d]	EOS[d]/FU[e]
**Procedure**	**Screen[a]**	**1[b]**	**8**	**15**	**21**	**1**	**8**	**15**	**21[c]**		
Concomitant Medications and Procedures	Recorded from 28 days prior to first dose of study drug through 30 days after the last dose of study drug or until the start of subsequent antineoplastic therapy, whichever occurs first										
Adverse Event Reporting		Recorded from first dose of study drug through 30 days after the last dose of study drug or until the start of subsequent antineoplastic therapy, whichever occurs first									
	**Serious adverse events** will be reported from signing of the informed consent form through 30 days after the last dose of study drug (see Section 7.4.18).[v]										
MLN8237 PK Samples[w]		X	X	X							
Administration of Weekly Paclitaxel[x]		X	X	X		X	X	X			
Administration of MLN8237[y]		C1D1–C1D3; C1D8–C1D10; C1D15–C1D17				D1–D3;D8–D10; D15–D17of each treatment cycle					
Patient Diary Review[z]			X	X	X	X[z]	X	X	X[z]	X	

Abbreviations: ANC = absolute neutrophil count; CR = complete response; CT = computed tomography; ECOG PS = Eastern Cooperative Oncology Group Performance Status; eCRF = electronic case report form; EOS = End of Study visit; EOT = End of Treatment visit; FU = Follow-up; h = hour(s); MRI = magnetic resonance imaging; PD = progressive disease; PFS = progression-free survival; PK = pharmacokinetics; RECIST = Response Evaluation Criteria in Solid Tumors; TSH = thyroid-stimulating hormone

Note: Each treatment cycle is 28 days in length. Tests and procedures should be done on schedule, but visit windows of ± 2 days are allowed (except as otherwise specified) occasionally for holidays, vacations, and other administrative reasons. **If extenuating circumstances prevent a patient from beginning treatment or completing a scheduled assessment within this time frame, the patient may continue in the study only with written permission of the medical monitor.**

a Within 28 days before the Cycle 1, Day 1 dose of study drug, unless otherwise specified

b Cycle 1, Day 1 is baseline. Cycle 1, Day 1 evaluations and procedures are to be performed within the 4 days prior to the first dose of any study drug, unless otherwise specified. A repeat of the procedure is not required on Cycle 1, Day 1 if screening procedures were performed within 4 days of the Cycle 1, Day 1 dose of study drug. The exception is the CA-125, as 2 CA-125 values are required to be collected prior to dosing at Cycle 1, Day 1

c In patients who tolerate study treatment through multiple cycles, the Day 21 evaluation can be done by telephone contact, with or without laboratory testing, at the discretion of the investigator, if in the prior 2 cycles the patient tolerated study treatment (ie, without the requirement for dose reduction or without ≥ Grade 3 treatment-related toxicity evaluated according to National Cancer Institute Common Terminology Criteria for Adverse Events [NCI CTCAE], version 4.02).

**Phase 2 – Patients With Recurrent Ovarian, Fallopian Tube, or Primary Peritoneal Cancer Randomized to MLN8237 Plus Weekly Paclitaxel or Weekly Paclitaxel Alone**											
		**Cycle 1 (28-Day Cycle)**				**Subsequent 28-Day Cycles**				**EOT[d]**	**EOS[d]/FU[e]**
		**Day**				**Day**					
**Procedure**	**Screen[a]**	**1[b]**	**8**	**15**	**21**	**1**	**8**	**15**	**21[c]**		

d For all patients, the EOT visit will be conducted 30 (+ 10) days after the last dose of study drug for all patients. Patients who enter the Follow-Up period (eg, patients who discontinue study drug treatment before the occurrence of progressive disease [PD]/death) will be followed off treatment and will complete an EOT visit and later complete an EOS visit. The EOS visit will be completed at the time the patient completes or withdraws from the Follow-Up period. Note: Patients who do not enter the Follow-Up period (eg, experience PD, death during study drug treatment) will complete the EOT visit only.

e Patients who do not experience PD will be followed off treatment once every 8 weeks until the occurrence of 110 progression-free survival (PFS) events (PD or death, whichever occurs first) are documented for this study. Patients may withdraw from the Follow-Up period before the occurrence of 110 PFS events for any of the following reasons: PD, withdrawal of informed consent, starting a different anticancer therapy, or lost to follow-up. The EOS visit will be completed at the time the patient completes or withdraws from the Follow-Up period. Patients who do not enter the Follow-Up period (eg, experience PD or die during active study treatment) will complete the EOT visit only.

f Informed consent may be obtained earlier and must be obtained before performance of any study-specific procedures.

g Including history of prior treatment and recurrence dates of ovarian cancer.

h The Cycle 1, Day 1 (baseline) symptom-directed physical examination is not required if the screening physical examination was conducted within the 4 days prior to administration of the Cycle 1, Day 1 dose of study drug. The symptom-directed physical examination will be performed within the 3 days prior to the beginning (Day 1) and on Day 8 (± 2 days), Day 15 (± 2 days), and Day 21 (± 2 days) of each treatment cycle. In patients who tolerate study treatment through multiple cycles, the Day 21 symptom-directed physical examination can become optional, at the discretion of the investigator, if in the prior 2 cycles the patient tolerated study treatment (ie, without the requirement for dose reduction or without ≥ Grade 3 treatment-related toxicity evaluated according to NCI CTCAE, version 4.02) (see also footnote c). This physical examination will be repeated at the EOT visit and at the EOS visit (for patients who are followed off treatment and complete or withdraw from the Follow-Up period).

i Vital signs (blood pressure, heart rate, and oral temperature) measurements will be obtained during screening; at Cycle 1, Day 1 (baseline); within 3 days before the beginning (Day 1) of each treatment cycle; on Day 8 (± 2 days), Day 15 (± 2 days), and Day 21 (± 2 days); at the EOT visit; and at the EOS visit (for patients who are followed off treatment and complete or withdraw from the Follow-Up period). Blood pressure should be determined with the patient in a seated position after the patient has been sitting quietly for 5 minutes. If the screening assessment was done within 4 days prior to Cycle 1, Day 1, an assessment at Cycle 1, Day 1 is not necessary.

j Weight will be measured during screening, at Cycle 1, Day 1 (baseline), within 3 days before the beginning (Day 1) of each treatment cycle, at the EOT visit, and at the EOS visit (for patients who are followed off treatment and complete or withdraw from the Follow-Up period). If the screening assessment was done within 4 days prior to Cycle 1, Day 1, an assessment at Cycle 1, Day 1 is not necessary.

k ECOG performance status will be assessed during screening; at Cycle 1, Day 1 (baseline); within 3 days before the beginning (Day 1) of each treatment cycle; at the EOT visit; and at the EOS visit (for patients who are followed off treatment and complete or withdraw from the Follow-Up period). Refer to Section 15.1. If the screening assessment was done within 4 days prior to Cycle 1, Day 1, an assessment at Cycle 1, Day 1 is not necessary.

l A 12-lead electrocardiogram (ECG) will be obtained at screening and at Cycle 1, Day 1. If the screening ECG was obtained within the 4 days prior to the Cycle 1, Day 1 dose of study drug, a repeat ECG at Cycle 1, Day 1 is not necessary. Additional ECGs may be obtained if clinically indicated. ECG

Phase 2 – Patients With Recurrent Ovarian, Fallopian Tube, or Primary Peritoneal Cancer Randomized to MLN8237 Plus Weekly Paclitaxel or Weekly Paclitaxel Alone											
		Cycle 1 (28-Day Cycle)				Subsequent 28-Day Cycles				EOT[d]	EOS[d]/FU[e]
		Day				Day					
**Procedure**	**Screen[a]**	1[b]	8	15	21	1	8	15	21[c]		

assessments are to be performed with patient supine and rested for 5 minutes and before any closely timed blood collection.

m A blood sample for hematology will be obtained during screening; at Cycle 1, Day 1 (baseline); within the 3 days before the beginning (Day 1) of each treatment cycle; on Day 8 (± 2 days), Day 15 (± 2 days), and Day 21 (± 2 days); and at the EOT visit. In patients who tolerate study treatment through multiple cycles, the Day 21 clinical laboratory assessment can become optional, at the discretion of the investigator, if in the prior 2 cycles the patient tolerated study treatment (ie, without the requirement for dose reduction or without ≥ Grade 3 treatment-related toxicity evaluated according to NCI CTCAE, version 4.02). Hematology includes hemoglobin, hematocrit, white blood cell (WBC) count with differential count (machine results acceptable), and platelet count. If screening values were obtained within the 4 days prior to Cycle 1, Day 1, repeat hematology testing at Cycle 1, Day 1 is not necessary. If a patient has an ANC less than 500/mm^3 or a platelet count less than 25,000/mm^3, or both, the CBC with differential should be repeated at least every 2 to 3 days until the ANC or platelet count (or both, if both were decreased) have exceeded these values on at least 2 occasions.

n A blood sample for serum chemistries will be obtained during screening; at Cycle 1, Day 1 (baseline); within 3 days before the beginning (Day 1) of each treatment cycle; and at the EOT visit. Serum chemistries include sodium, potassium, blood urea nitrogen (BUN), creatinine, alkaline phosphatase, AST (SGOT), ALT (SGPT), total bilirubin, carbon dioxide, albumin, calcium, chloride, phosphate, magnesium, and glucose. Thyroid stimulating hormone (TSH) level will be measured at Cycle 1, Day 1 (baseline) and at the EOT visit; this test should be repeated during the treatment period as clinically indicated. If screening values were obtained and acceptable within the 4 days prior to Cycle 1, Day 1, repeat clinical chemistry testing at Cycle 1, Day 1 is not necessary.

o A urine sample for urinalysis will be obtained during screening; at Cycle 1, Day 1 (baseline); and at the EOT visit. Urinalysis includes testing for protein and blood. If urine protein by dipstick changes to 3+ (or 2+ that is reconfirmed at least 1 day later), then a 24-hour urine collection should be done for protein and creatinine clearance. The screening or baseline urinalysis should include microscopic examination of the sediment. If screening values were obtained and acceptable within the 4 days prior to the Cycle 1, Day 1 dose of study drug, a repeat urinalysis at Cycle 1, Day 1 is not necessary.

p A serum β-hCG pregnancy test will be performed only for patients of childbearing potential during screening and again at Cycle 1, Day 1 if the screening test was performed more than 4 days before the first dose of any study drug. The results must be negative within 4 days before the first dose of paclitaxel or MLN8237 is administered (ie, within the 4 days prior to Cycle 1, Day 1), or as otherwise required by local regulations. Additional pregnancy testing may be performed during the study at the discretion of the investigator, as per request of IEC/IRB, or if required by local regulations.

q CA-125 levels will be obtained according to standard of care within 9 days of screening and sufficient time after prior therapy; within 4 days prior to the dose at Cycle 1, Day 1; at the end of every treatment cycle, within the week prior to the start of a new cycle; at the EOT visit; and at the EOS visit (for patients who are followed off treatment and complete or withdraw from the Follow-Up period) (see Section 15.4). The investigator's determination of response by CA-125 is required at the end of every 2 cycles, designed to coincide with imaging studies for evaluation of response by RECIST criteria, if applicable.Two CA-125 levels must be obtained prior to dosing on Cycle 1, Day 1. CA-125 should be evaluated to determine if PD is substantiated prior to the start of each new cycle. Patients who discontinue study drug treatment before the occurrence of PD will be assessed at the EOT visit and at follow-up visits once every 8 weeks until the occurrence of 110 PFS events (PD or death, whichever occurs first). It is noted that confirmation of response by CA-125 levels must be obtained at least 28 days after the previous sample. Response evaluation employs modified Gynecologic Cancer Intergroup (GCIG) criteria for changes in CA-125.

r Extent of disease evaluation by the Response Evaluation Criteria in Solid Tumors (RECIST), version 1.1, includes computed tomography (CT) with IV

Phase 2 – Patients With Recurrent Ovarian, Fallopian Tube, or Primary Peritoneal Cancer Randomized to MLN8237 Plus Weekly Paclitaxel or Weekly Paclitaxel Alone											
		Cycle 1 (28-Day Cycle)				Subsequent 28-Day Cycles					
		Day				Day				EOT[d]	EOS[d]/FU[e]
Procedure	Screen[a]	1[b]	8	15	21	1	8	15	21[c]		

contrast of the abdomen and pelvis, as well as other anatomical areas as appropriate based on the known prior extent of the patient's disease and clinical symptoms (see Section 15.3). Magnetic resonance imaging (MRI) scans may be used to assess sites of disease not adequately imaged by CT. If the patient has had appropriate scans performed within 28 days before the Cycle 1, Day 1 dose of MLN8237, then those scans may be used for tumor lesion measurement during screening. Repeat CT (with IV contrast) or MRI scans, or both, are to be performed at the completion of Cycle 2 and at the completion of every 2 cycles (approximately every 8 weeks, between Days 21 and 28 of every even cycle) thereafter for up to 24 months or until PD is documented (except as permitted per agreement with the Investigator and Medical Monitor-Section 7.5). The same imaging modality should be used throughout the study for each site of disease. For responders, radiographic images will be read locally but will be collected and provided to the sponsor for subsequent review. Scans are required at the EOT visit only if PD has not been documented previously and it has been 8 weeks or more since the previous evaluation. Other procedures, such as physical examinations and other scintigraphic examinations (eg, bone scans for patients with known or suspected bone metastases), also should be taken into consideration when evaluating the extent of malignant disease. Patients who discontinue study drug treatment before occurrence of PD will be assessed at EOT and follow-up visits once every 8 weeks until the occurrence of 110 PFS events (PD or death, whichever occurs first).

s Banked tumor tissue (submitted paraffin-embedded block, if available, otherwise slides) will be obtained from a previous resection or biopsy that was done as part of the patient's standard care. [redacted]

t One blood sample will be obtained at Cycle 1, Day 1 before administration of the first dose of study drug (may be obtained within the 4 days prior to Cycle 1, Day 1) [redacted]

u Blood samples will be obtained at Cycle 1, Day 1 before the first dose of MLN8237 and at the scheduled visit on Day 15 of Cycle 1. In subsequent cycles, a blood sample will be obtained on Day 8 of **Cycle 2 only**. [redacted] in serum isolated from these blood samples. Details regarding the preparation, handling, and shipping of samples are provided in the Study Manual.

v Only those serious events that occur after the first dose of any study drug will be **entered** in the eCRF, **although all serious adverse events and serious pretreatment events must be reported to Millennium Pharmacovigilance or designee**.

w For the treatment arm in which MLN8237 is administered with paclitaxel, MLN8237 PK measurements will be performed during Cycle 1 at the following time points: On Day 1 before administration of the MLN8237 dose, before initiation of the paclitaxel infusion, and following completion of the paclitaxel infusion; and on Day 8 before administration of the MLN8237 dose and at the end of the paclitaxel infusion; and on Day 15 at the time of the clinic visit. The date and time of PK blood sampling and the date and time of administration of the 2 doses of MLN8237 immediately preceding each PK sample should be recorded on the eCRF based on the entries in the patient's dosing diary or site source documentation.

x Paclitaxel will be administered as an intravenous (IV) infusion on Days 1, 8, and 15 of each 28-day treatment cycle. Premedication may be administered in accordance with the recommendations described in the product label. Modifications to paclitaxel infusion are allowed upon agreement by the medical

**Phase 2 – Patients With Recurrent Ovarian, Fallopian Tube, or Primary Peritoneal Cancer Randomized to MLN8237 Plus Weekly Paclitaxel or Weekly Paclitaxel Alone**											
		**Cycle 1 (28-Day Cycle)**				**Subsequent 28-Day Cycles**					
		**Day**				**Day**				**EOT[d]**	**EOS[d]/FU[e]**
**Procedure**	**Screen[a]**	**1[b]**	**8**	**15**	**21**	**1**	**8**	**15**	**21[c]**		

monitor and will be documented in the eCRF. If the paclitaxel starting dose is less than 80 mg/m^2 during the Phase 1 study, then dose escalation is allowed after Cycle 2 up to a maximum of 80 mg/m^2 given lack of CTC Grade 3 toxicities in early cycles in early cycles using a reduced dose of paclitaxel. Upon review of dose escalation experience from Phase 1 by the sponsor and Phase 1 investigators, the option for paclitaxel escalation will be permitted in Phase 2 for individual patients who do not experience CTC Grade 3 or worse toxicities in early cycle(s).

y For the combination regimen of MLN8237 plus weekly paclitaxel, on days when both MLN8237 and paclitaxel are administered, MLN8237 should be administered 1 hour before the start of the paclitaxel infusion. MLN8237 will be dispensed to the patient on Day 1.

z The study center staff will check the patient diary versus the patient's supply of MLN8237 tablets on Day 8 (for Days 1 to 3), on Day 15 (for Days 8 to 10), and on Day 21 (for Days 15 to 17), as applicable, of each treatment cycle and at the EOT visit to ensure compliance. For patients who do not require a clinic visit on Day 21, the medication count may be confirmed verbally with the patient by telephone at this visit; however, a check of the patient diary versus the patient's supply of MLN8237 tablets should be performed at the next visit (eg, on Day 1 of the subsequent cycle or at the EOT visit, if applicable).

TABLE OF CONTENTS

LIST OF TABLES....25
LIST OF ABBREVIATIONS AND GLOSSARY OF TERMS....26
1. BACKGROUND AND STUDY RATIONALE....29
1.1 Scientific Background....29
1.1.1 Disease Under Treatment....29
1.1.2 Aurora Kinases and the Aurora A Kinase Inhibitor MLN8237....31
1.1.3 Paclitaxel....32
1.2 Preclinical Experience with MLN8237....35
1.2.1 In Vitro Studies....35
1.2.2 In Vivo Studies....35
1.2.3 Safety Pharmacology, Toxicology, and Drug Absorption and Metabolism....36
1.3 Clinical Experience With MLN8237....38
1.3.1 Pharmacokinetics....39
1.4 Study Rationale....40
1.4.1 Rationale for Assessment of Treatment With MLN8237 and Weekly Paclitaxel....40
1.4.2 Rationale for Pharmacokinetic Assessments....42
1.4.3 Rationale for Assessment of Biomarkers in Archived Tumor Tissue....42
1.4.4 Rationale for Genotyping....43
1.4.5 Rationale for Serum Assessment of Tumor Response....44
1.5 Potential Risks and Benefits....44
2. STUDY OBJECTIVES....45
2.1 Phase 1 Objectives....45
2.1.1 Phase 1: Primary Objectives....45
2.1.2 Phase 1: Secondary Objectives....45
2.1.3 Phase 1: Exploratory Objective....45
2.2 Phase 2 Objectives....46
2.2.1 Phase 2: Primary Objective....46
2.2.2 Phase 2: Secondary Objectives....46
2.2.3 Phase 2: Exploratory Objectives....46
3. STUDY ENDPOINTS....47
3.1 Phase 1 Endpoints....47
3.1.1 Phase 1: Primary Endpoints....47
3.1.2 Phase 1: Secondary Endpoints....47
3.1.3 Phase 1: Exploratory Endpoints....47
3.2 Phase 2 Endpoints....48
3.2.1 Phase 2: Primary Endpoint....48
3.2.2 Phase 2: Secondary Endpoints....48
3.2.3 Phase 2: Exploratory Endpoints....48
4. STUDY DESIGN....49
4.1 Overview of Study Design....49
4.2 Number of Patients....53
4.3 Duration of Study....53

5. STUDY POPULATION 54
5.1 Inclusion Criteria 54
5.2 Exclusion Criteria 57
6. STUDY DRUG 60
6.1 Study Drug Administration 60
6.2 Reference/Control Therapy 61
6.2.1 Paclitaxel 61
6.2.2 Premedication for Paclitaxel-Associated Hypersensitivity or Other Acute Reactions 61
6.3 Definitions of Dose-Limiting Toxicity 62
6.4 Dose Escalation Rules 63
6.5 Dose-Modification Guidelines 66
6.5.1 Criteria for Beginning a Subsequent Treatment Cycle 66
6.5.2 Criteria for Dose Interruption During a Cycle 67
6.5.3 Dose Modifications 68
6.6 Excluded Concomitant Medications and Procedures 71
6.7 Permitted Concomitant Medications and Procedures 72
6.8 Precautions and Restrictions 73
6.9 Management of Clinical Events 73
6.9.1 Nausea and Vomiting 73
6.9.2 Diarrhea 74
6.9.3 Central Nervous System Effects 74
6.9.4 Hypersensitivity Reactions Associated with Paclitaxel 74
6.10 Blinding and Unblinding 75
6.11 Description of Investigational Agents 75
6.12 Preparation, Reconstitution, and Dispensation 75
6.13 Packaging and Labeling 75
6.14 Storage, Handling, and Accountability 76
6.15 Other Protocol-Specified Materials 77
7. STUDY CONDUCT 77
7.1 Study Personnel and Organizations 77
7.2 Arrangements for Recruitment of Patients 77
7.3 Treatment Group Assignments 77
7.4 Study Procedures 78
7.4.1 Informed Consent 78
7.4.2 Inclusion and Exclusion Criteria 78
7.4.3 Patient Demographics 79
7.4.4 Medical History and Physical Examination 79
7.4.5 Vital Signs 80
7.4.6 Patient Height 80
7.4.7 Patient Weight 80
7.4.8 Eastern Cooperative Oncology Group Performance Status 81
7.4.9 Electrocardiogram 81
7.4.10 Clinical Laboratory Evaluations 81
7.4.11 Pregnancy Test 83
7.4.12 Disease Assessment by CA-125 83

7.4.13 Extent of Disease Evaluation by RECIST, Version 1.1 84
7.4.14 Response Markers in Banked Tumor Specimens 85
7.4.15 Blood Sample for Genotyping 85
7.4.16 Blood Sample for Serum Markers of Response...86
7.4.17 Concomitant Medications and Procedures 86
7.4.18 Adverse Events 86
7.4.19 Pharmacokinetic Measurements 87
7.4.20 Pharmacodynamic Measurements 88
7.4.21 Administration of Paclitaxel 88
7.4.22 Administration of MLN8237 89
7.4.23 Patient Diary 90
7.5 Completion of Treatment..91
7.6 Discontinuation of Treatment With Study Drug, and Patient Replacement................92
7.7 Withdrawal of Patients From Study Follow-Up...92
7.8 Study Compliance...93
8. STATISTICAL AND QUANTITATIVE ANALYSES...94
8.1 Statistical Methods 94
8.1.1 Determination of Sample Size 94
8.1.2 Randomization and Stratification 95
8.1.3 Populations for Analysis...95
8.1.4 Procedures for Handling Missing, Unused, and Spurious Data 96
8.1.5 Demographic and Baseline Characteristics 96
8.1.6 Efficacy Analysis..97
8.1.7 Pharmacokinetics/Pharmacodynamics/Biomarkers...98
8.1.8 Safety Analysis 99
8.1.9 Interim Analysis..101
8.2 Pharmacokinetic Modeling...101
9. STUDY COMMITTEES 102
9.1 Safety Monitoring Committee 102
10. ADVERSE EVENTS...102
10.1 Definitions 102
10.1.1 Pretreatment Event Definition 102
10.1.2 Adverse Event Definition 102
10.1.3 Serious Adverse Event Definition 103
10.2 Procedures for Recording and Reporting Adverse Events and Serious Adverse Events 104
10.3 Monitoring of Adverse Events and Period of Observation 105
10.4 Procedures for Reporting Drug Exposure During Pregnancy and Birth Events 106
11. ADMINISTRATIVE REQUIREMENTS 106
11.1 Good Clinical Practice..106
11.2 Reporting of Suspected Unexpected Serious Adverse Reactions 106
11.3 Data Quality Assurance 107
11.4 Electronic Case Report Form Completion..107
11.5 Study Monitoring..108
11.6 Ethical Considerations..108
11.7 Patient Information and Informed Consent 108

11.8 Patient Confidentiality 108
11.9 Investigator Compliance 109
11.10 On-site Audits 109
11.11 Investigator and Site Responsibility for Drug Accountability 109
11.12 Product Complaints and Medication Errors 109
11.13 Closure of the Study 110
11.14 Record Retention 111
12. USE OF INFORMATION 111
13. INVESTIGATOR AGREEMENT 112
14. REFERENCES 113
15. APPENDICES 117
15.1 Eastern Cooperative Oncology Group (ECOG) Scale for Performance Status 117
15.2 Cockcroft-Gault Equation 117
15.3 Modified Response Evaluation Criteria in Solid Tumors (RECIST, Version 1.1) 118
15.4 Gynecologic Cancer Intergroup (GCIG) Modified Response Criteria 118
15.5 Distribution of Active Bone Marrow in the Adult 118
15.6 Phase 1 – Schedule of Pharmacokinetic Sampling for Paclitaxel and MLN8237 119
15.7 Amendment 1 Rationale and Purposes 120
15.8 Amendment 2 Rationale and Purposes 124
15.9 Amendment 3 Detailed Summary of Changes 127

LIST OF TABLES

Table 6-1 MLN8237 Combined With Paclitaxel for Phase 1 Dose Escalation 65
Table 6-2 Dose Modification Rules – Hematological Toxicity Attributable to Taxane with or without MLN8237 69
Table 8-1 Best Overall Combined Response 98

LIST OF ABBREVIATIONS AND GLOSSARY OF TERMS

Abbreviation	Term
5-HT_3	5-hydroxytryptamine 3 (serotonin receptor)
AE	adverse event
ALT (SGPT)	alanine transaminase (serum glutamic pyruvic transaminase)
ANC	absolute neutrophil count
ASCO	American Society of Clinical Oncology
AST (SGOT)	aspartate transaminase (serum glutamic oxaloacetic transaminase)
ATP	adenosine triphosphate
AUC	area under the plasma concentration versus time curve
$AUC_{0-\tau}$	area under the plasma concentration versus time curve zero to the end of the dosing interval
$AUC_{0-\infty}$	area under the plasma concentration versus time curve zero to infinity
$AUC_{0-tlast}$	area under the plasma concentration versus time curve zero to the time of the last measurement
β–hCG	beta-human chorionic gonadotropin
BID	bis in die; twice a day
BUN	blood urea nitrogen
CBC	complete blood count
CBR	clinical benefit rate
C_{avg}	average plasma concentration
CD	compact disk
C_{max}	maximum plasma concentration
C_{ss}	steady-state plasma concentration
CI	confidence interval
[redacted]	
CNS	central nervous system
CO_2	carbon dioxide
CR	complete response
CRO	Contract Research Organization
CT	computed tomography
CYP	cytochrome P_{450}
DLT	dose-limiting toxicity
DNA	deoxyribonucleic acid
DLBCL	diffuse large B cell lymphoma
DOR	duration of response
EC_{90}	efficacious concentration producing 90% of the maximal possible response
ECG	electrocardiogram
ECT	enteric-coated tablet
ECOG	Eastern Cooperative Oncology Group
eCRF	electronic case report form
EDC	electronic data capture
E_{max}	maximum effect
EOS	End of Study (visit)

Abbreviation	Term
EOT	End of Treatment (visit)
FDA	Food and Drug Administration
GABAAα1	gamma aminobutryic acid alpha 1
GCIG	Gynecologic Cancer Intergroup
GCP	Good Clinical Practice
GI_{50}	50% cell-growth inhibitor concentrations
HDPE	high-density polyethylene
hERG	human ether-à-go-go related gene
HIV	human immunodeficiency virus
IB	Investigator's Brochure
IC_{50}	concentration producing 50% inhibition
ICF	informed consent form
ICH	International Conference on Harmonisation
IEC	Independent Ethics Committee
IRB	Institutional Review Board
IV	intravenous; intravenously
K_i	inhibition constant
LLN	lower limit of normal
MedDRA	Medical Dictionary for Regulatory Activities
Millennium	Millennium Pharmaceuticals, Inc., and its affiliates
mITT	modified intent-to-treat
MRI	magnetic resonance imaging
mRNA	messenger ribonucleic acid
MTD	maximum tolerated dose
NCI CTCAE	National Cancer Institute Common Terminology Criteria for Adverse Events
NPO	nothing by mouth
OC	ovarian cancer, including epithelial ovarian, fallopian tube, and primary peritoneal carcinoma
ORR	overall response rate
OS	overall survival
PD	progressive disease (disease progression)
PFS	progression-free survival
Pgp	P-glycoprotein
PIC	powder-in-capsule
PK	pharmacokinetic(s)
PO	per os; by mouth (orally)
PR	partial response
PS	Performance Status
Pt-R OC	platinum-resistant ovarian cancer
QD	quaque die; each day; once daily
RECIST	Response Evaluation Criteria in Solid Tumors
RP2D	recommended phase 2 dose
SAE	serious adverse event
SC	subcutaneous

Abbreviation	Term
SD	stable disease
SMC	Safety Monitoring Committee
[redacted]	
Study drug	MLN8237 or paclitaxel
Study drug treatment	MLN8237 plus weekly paclitaxel or weekly paclitaxel alone
SUSAR	suspected unexpected serious adverse reaction
$t_{1/2}$	half-life
TGI	tumor growth inhibition
T_{max}	time to maximum plasma concentration
TTP	time to progression
UGT	uridine diphosphate glucuronosyltransferase
ULN	upper limit of the normal range
US	United States
WBC	white blood cell
WHO	World Health Organization

1. BACKGROUND AND STUDY RATIONALE

1.1 Scientific Background

1.1.1 Disease Under Treatment

1.1.1.1 Epithelial Ovarian Cancer Epidemiology and Treatment

Ovarian cancer (OC) continues to be the leading cause of death from gynecological malignancies and ranks as the fourth most common cause of cancer mortality in women. In 2007, more than 22,000 new cases of OC were reported in the United States (US), with over 15,200 deaths estimated by the American Cancer Society.[(5)] Unfortunately, in most patients OC is diagnosed at advanced stages, indicating spread beyond the pelvis at the time of diagnosis. Disease dissemination results in significant morbidity and impaired survival in comparison with those who have disease diagnosed while it is still confined to the ovaries (stage I), in whom over 90% survival can be achieved largely with surgical intervention. Patients with advanced-stage disease have 5-year overall survival (OS) rates ranging from 20% to 30%.

Systemic treatments commonly employ a combination of platinum and taxane chemotherapy. Strategies incorporating biological and targeted agents continue to evolve. Unfortunately, most (up to 70%) patients who present with advanced-stage disease exhibit recurrent or persistent disease following primary treatment. Current management of recurrent OC generally is guided by the interval of time until recurrence of disease. Patients generally are classified into platinum-resistant and platinum-sensitive subgroups; these latter categories are defined based on the length of time from completing treatment with a prior platinum-containing therapy to the time of progression.

Platinum-resistant ovarian cancer (Pt-R OC) is commonly defined in patients who relapse within 0 to 6 months of discontinuation of prior platinum-containing therapy, although alternative (but similar) definitions have been employed.[(6)] A subgroup of patients also experience progression during primary or subsequent platinum-based therapy, or persistent radiographic disease during and after primary or subsequent platinum-based therapy, and are described as having platinum-refractory disease.

A recent analysis of patients with recurrent OC, who achieved favorable disease control after platinum retreatment, revealed that the platinum-free interval over 12 months corresponded with the best clinical outcomes from platinum retreatment.[(7)] These latter data

support new development of non-platinum treatment options for patients who develop progressive disease (PD) during the period from 0 to 12 months after completion of a prior platinum regimen, as described in the eligibility criteria for patients enrolled to this study.

Patients who experience a recurrence more than 12 months after discontinuation of platinum therapy are commonly considered to have platinum-sensitive disease. These patients are candidates for retreatment with a platinum-containing regimen. Although platinum regimens are commonly active in this setting, patients who achieve remission continue to experience additional recurrences over the next few years with median time to relapse of 9 to 10 months in some series.[8, 9] Patients with platinum-sensitive recurrence diagnosed more than 12 months after discontinuation of a platinum-containing regimen are not eligible for enrollment in this study.

For systemic treatment of patients with recurrent OC who are not candidates for retreatment with platinum, some of the most commonly used agents include liposomal doxorubicin, topotecan, gemcitabine, and weekly paclitaxel. Liposomal doxorubicin is often used in the second-line setting. The overall clinical benefit from this agent, however, has been marginal. Although liposomal doxorubicin is generally tolerable with treatments managed in the outpatient setting, studies have shown median progression-free survival (PFS) to be generally less than 4 months and response rates under 25%, and the agent has not been proven to offer reliable improvements in OS. Given the limitations of current treatment options, recurrent OC remains an unmet medical need, and new approaches are needed to improve progression-free and overall survival. For a review of recent studies using weekly paclitaxel in patients with recurrent OC, see Section 1.1.3.

1.1.1.2 Advanced Breast Cancer

In the management of advanced breast cancer, weekly paclitaxel also has been reported to have clinical utility, including experience in heavily pretreated patients with disease that is resistant to other chemotherapies.[10] Thus, enrollment of women with advanced breast cancer who are candidates for weekly paclitaxel will be allowed in the Phase 1 portion of this study. Although the number of patients with breast cancer will be limited, and no statistical comparisons are planned, the findings from this subset of patients with breast cancer enrolled in the Phase 1 portion will contribute to the understanding of the safety, tolerability, and pharmacokinetics (PK) of the combination regimen across more than 1 tumor indication. Combined with results from patients with recurrent OC enrolled in the randomized, Phase 2 portion, the safety results in Phase 1 will provide an opportunity to

support future applications of the regimen for Phase 2 development in other indications, including metastatic breast cancer.

1.1.2 Aurora Kinases and the Aurora A Kinase Inhibitor MLN8237

The Aurora kinases (A, B, and C) are a family of serine/threonine kinases that play an important role in both normal and aberrant cell division. Aurora A functions primarily in the G2/M phase of cell division and is associated with centrosome maturation, mitotic entry, spindle assembly, and microtubule organization.(11, 12) The Aurora A kinase gene is located on chromosome 20q13.2, which is often amplified in malignancies, including cancers of the ovary, breast, colon, pancreas, bladder, liver, and stomach.(11)

Specifically, Aurora A activity is overexpressed in up to 83% of human epithelial ovarian cancers.(13) Aurora A kinase overexpression has been demonstrated to be a negative prognostic marker in cancers such as breast and ovary.(14) In addition to promoting tumor growth, Aurora A overexpression is linked to resistance to common anticancer agents used to treat OC, such as paclitaxel and cisplatin.(13, 15, 16)

MLN8237 is an orally (PO) administered synthetic small molecule inhibitor of Aurora A kinase.(11, 14) The molecule binds to Aurora A kinase and inhibits its activity. In preclinical studies, the drug demonstrated broad antitumor activity in vitro and in vivo, including synergism when administered in combination with taxanes in human tumor xenografts.(11, 14) The safety and activity of MLN8327 using several dosing schedules are being investigated in ongoing, phase 1, clinical studies; the available data indicate that bioactive exposures can be achieved with generally tolerated doses administered for periods of up to 14 days of dosing followed by a treatment-free period within a 28-day treatment cycle.

As a single agent, MLN8237 has been evaluated in two phase 1 studies in patients with a diversity of solid tumors, and the agent was generally tolerated in patients with OC and other advanced solid tumors enrolled in these studies. The dose-limiting toxicities (DLTs) have been largely mechanistic (hematological toxicities, mucositis, and alopecia), monitored by routine laboratory assessments and clinical examinations, and manageable by dose interruption or reduction and supportive care.

Responses to single-agent MLN8237 have been observed in patients with advanced solid tumors, including 1 patient with chemotherapy- and radiation-resistant OC with clear cell histology who experienced a partial response (PR) by Response Evaluation Criteria in Solid Tumors (RECIST), which has been durable over 1 year with continued treatment.(17) A

phase 2 study (C14006) has been initiated in patients with platinum-resistant OC. Although the results are preliminary, modest antitumor activity of single-agent MLN8237 was reconfirmed in this disease based on objective responses or stabilization of disease. After 2 cycles of MLN8237, radiographic tumor regression was observed in a patient with a large retroperitoneal pleomorphic liposarcoma, refractory to multiple other treatments. While PR was not achieved, the patient's disease was considered stable. Although the patient's dose was reduced to manage toxicities, he remained on study without disease progression for longer than 1 year (more than 20 cycles).

Millennium Pharmaceuticals, Inc. (Millennium) has developed a new formulation of MLN8237, an enteric-coated tablet (ECT), for future development and commercialization. [redacted] Protocol C14001 was amended in 2009 to include evaluation of safety and PK (relative bioavailability) after treatment with single-agent MLN8237 as the ECT formulation. [redacted]

A common formulation process and materials were used in the manufacture of the unit-dose strengths of the tablets planned for this study (10 mg or 50 mg ECT). Both tablet strengths exhibited similar dissolution properties. These features support the expectation of consistent absorption, PK properties, and consequently tolerability after administration of equivalent total doses with the different strength tablets.

1.1.3 Paclitaxel

Paclitaxel provides biological activity in the management of patients with platinum-resistant OC, and this agent is considered to be a standard of care for routine clinical use in this setting. Although the early studies employed higher doses administered in 21-day schedules, more recent experience from a number of completed single-arm and randomized studies also supports the clinical utility of weekly paclitaxel regimens, such as a starting dose of 80 mg/m^2 administered on Days 1, 8, and 15 followed by a rest period in 28-day cycles. A prospective, multicenter, single-arm, phase 2 study was conducted by the Gynecologic Oncology Group to evaluate a starting paclitaxel dose of 80 mg/m^2 with the

option to reduce the individual weekly dose to 70 mg/m^2 or 60 mg/m^2 when required because of toxicity.[18] Forty-eight patients were evaluable, and the safety experience revealed that the most common toxicity was neuropathy (21% Grade 2, and 4% Grade 3). Hematological toxicities generally were tolerable: Grade 3 neutropenia was observed in 2 patients (4%), and Grade 2 thrombocytopenia was observed in 1 patient (2%). The overall objective response rate was 20.9%, including 2 complete and 8 partial responses. Of note, some of the patients who responded to the weekly regimen had previously experienced PD during or shortly after prior regimens that included the higher doses of paclitaxel administered in a 3-week regimen. These latter results suggested that the weekly paclitaxel regimen could provide superior disease control in some patients when compared with the older schedule using higher doses (eg, 135 to 175 mg/m^2) administered every 3 weeks.

A randomized, 2-arm, comparative study was conducted in 208 patients with recurrent OC previously treated with platinum to evaluate safety and clinical outcome after treatment with paclitaxel administered weekly or every 3 weeks. The median delivered dose intensity was 77.6 mg/m^2/week in the weekly arm and 72.7 mg/m^2/week in the 3-weekly arm. Severe (World Health Organization [WHO] Grade 3-4) hematological and nonhematological toxicities occurred more frequently in patients enrolled in the 3-weekly arm. No difference was observed between treatment arms in terms of response rate, time to progression, or survival. The authors concluded that the weekly paclitaxel regimen was associated with a better safety profile and appeared to be as effective as equivalent doses administered every 3 weeks.[19]

Supporting the clinical activity of weekly paclitaxel are the results from a randomized, phase 3, 2-arm study in the frontline setting conducted by the Japanese Gynecologic Oncology Group, which compared a combination of platinum with either weekly or 3-weekly paclitaxel.[20] A total of 637 patients with advanced OC were randomized to first-line treatment with carboplatin (area under the curve [AUC] 6) combined with either paclitaxel at 180 mg/m^2 on Day 1 (repeated every 3 weeks, control regimen) or weekly paclitaxel at 80 mg/m^2 on Days 1, 8, and 15. After a median follow-up of 29 months, median duration of PFS in the control versus the weekly group was 17.2 and 28.0 months, respectively ($p = 0.0015$ by the log-rank test), and OS at 3 years was 65.1% and 72.1%, respectively ($p = 0.03$). The authors concluded that the platinum combined with weekly paclitaxel regimen of 80 mg/m^2 on Days 1, 8, and 15 was superior to the older regimen of platinum combined with single-dose paclitaxel repeated every 3 weeks.

Another study conducted at a single European cancer center also reported the clinical activity of weekly paclitaxel in 53 patients with relapsed disease.[21] In this retrospective analysis, the response rate was 48% by radiological criteria and 69% by CA-125 assessment. No Grade 4 toxicities were reported; and Grade 3 toxicities were fatigue (13%), peripheral neuropathy (11%), and neutropenia (8%). The median PFS was 4.8 months and median OS was 13.5 months. Although retrospective comparisons may be confounded as the patients were not randomized prospectively to balance prognostic factors, the authors reported no significant difference in efficacy of the weekly paclitaxel between 24 patients previously treated with taxanes (radiological response 43%; CA-125 response 63%) and 29 patients who had not received prior taxanes (radiological response 52%; CA-125 response 76%). The authors concluded that weekly paclitaxel is a well tolerated and active regimen in patients with previously treated OC and its use in recurrent disease is likely to increase.

In patients with relapsed, platinum-sensitive disease, weekly paclitaxel combined with carboplatin has been evaluated in a prospective, phase 2 study.[22] Twenty-nine patients were treated with carboplatin at an AUC of 2 and paclitaxel 80 mg/m^2 on Days 1, 8, and 15 of a 28-day cycle. Hematological toxicities were common (32% Grade 3 neutropenia, no Grade 4 neutropenia, and 14% Grade 3 or 4 thrombocytopenia) but manageable by treatment delay, dose reduction, or discontinuation of 1 of the agents. Even with these dose modifications, the overall efficacy outcomes were favorable, and 21 platinum-sensitive patients had a 100% response rate. In a subgroup of 8 patients with platinum-refractory disease, the response rate was 37.5%. Median time to progression (TTP) was 13.7 months among platinum-sensitive patients and 3.2 months among platinum-refractory patients. Overall median TTP was 11.5 months and median duration of response (DOR) was 9.9 months. Taken together with the favorable outcomes from the phase 3 study of weekly paclitaxel combined with platinum in the frontline setting,[20] the results indicate that combinations of weekly paclitaxel and other anticancer agents are feasible, with manageable toxicities, and may improve clinical outcomes.

In view of the manageable toxicities and promising efficacy observed in several studies when weekly paclitaxel was administered as a single agent to patients with recurrent OC, the combination with MLN8237 may provide improved clinical benefit with a toxicity profile that can be managed and monitored in the outpatient setting.

1.2 Preclinical Experience with MLN8237

1.2.1 In Vitro Studies

MLN8237 is an adenosine triphosphate (ATP)-competitive and reversible inhibitor of Aurora A kinase with an inhibition constant (K_i) of 0.43 nM. In HCT-116 human colorectal tumor cells, MLN8237 produces 50% inhibition of Aurora A kinase activity at a concentration of 6.7 nM. It is approximately 200-fold more selective for Aurora A kinase than the structurally related family member, Aurora B kinase (concentration producing 50% inhibition [IC_{50}] = 1534 nM). Moreover, MLN8237 is selective for Aurora A kinase when compared to most other kinases (at a minimum 250-fold selective in vitro against those screened in concentration-response assays) and receptors. MLN8237 has affinity for the gamma-aminobutryic acid alpha 1 (GABAAα1) receptor benzodiazepine binding site (K_i = 290 nM). The consequences of GABAA binding in rat and dog safety pharmacology studies are discussed below.

The in vitro antiproliferative effect of MLN8237 was quantified in tumor cell lines derived from a variety of malignancies, including colon (3 cell lines), breast (1 cell line), lung (1), ovary (1), prostate (1), pancreas (1), and lymphoid (1). MLN8237 inhibited proliferation with lethal concentrations for 50% cell-growth inhibitor concentrations (GI_{50} values) ranging from 16 to 469 nM, demonstrating that MLN8237 is a potent inhibitor of proliferation in diverse human tumor cell lines.

1.2.2 In Vivo Studies

MLN8237 has demonstrated broad antitumor activity in a diverse array of experimental human tumor xenografts when dosed QD or BID. These include 2 colon models (HCT-116 and DLD-1), 2 lung models (H460 and Calu-6), 1 breast model (MDA-MB-231 FP4), 1 prostate model (CWR22 RV-1 Luc1.17), and 4 diffuse large B-cell lymphoma (DLBCL) models (Ly19, WSU, Ly7, PHTX-22-06). Statistically significant tumor growth inhibition (TGI) was observed with MLN8237 given at 30 mg/kg QD or less in all models except the Calu-6 model. At 20 mg/kg BID or less, statistically significant TGI also was observed in all models tested, including Calu-6. Taken together, these results demonstrated that MLN8237 has broad-based antitumor activity in many experimental human tumor models.

MLN8237 also demonstrated robust antitumor activity when combined with a taxane in preclinical models. MLN8237 dosed daily at 3, 10, or 20 mg/kg QD was tested with docetaxel dosed weekly at 5 or 10 mg/kg in mice bearing breast (MDA-MB-231), primary

breast (PHTX-02), lung (H522), or prostate (CWR22 RV-1 Luc1.17) human tumor xenografts. The various combinations of these agents at these doses were well tolerated and resulted in synergistic or additive antitumor activity relative to the single-agent activity of either MLN8237 or docetaxel alone. In several of these models, the combination of MLN8237 and docetaxel led to regression in tumor size and a dramatic prolongation in survival after termination of treatment. When the taxane was delivered in a regimen that overlapped MLN8237 exposure during the first week of dosing, major enhanced antitumor activity was observed when MLN8237 was administered over a shorter (9-day) time period, and the magnitude of response and disease control was comparable to results obtained with longer 21-day dosing schedule.

To define the plasma concentration of MLN8237 that inhibits Aurora A, the relationship between PK and pharmacodynamics was assessed at the steady-state concentration (C_{ss}). Steady-state MLN8237 concentrations were achieved using osmotic mini-pumps implanted subcutaneously (SC) in mice bearing HCT-116 xenografts. The relationship between the C_{ss} of MLN8237 and the tumor mitotic index fit the sigmoid maximum effect (E_{max}) model with an estimated efficacious concentration producing 90% of the maximal possible response (EC_{90}) of approximately 1 μM.

The HCT-116 tumor model was also used to determine the antitumor activity of MLN8237 given either QD or BID. MLN8237 demonstrated dose-dependent TGI whether administered QD or BID. TGI with BID administration of 3 mg/kg (TGI = 70%) and 10 mg/kg (TGI = 102%) was greater than with QD administration when measured on the last day of treatment (Day 21).

The relationship between efficacy and systemic exposure was investigated using osmotic mini-pumps implanted SC to deliver sustained release of MLN8237 over 12 days to HCT-116 xenografts. The relationship between the efficacy (TGI) on Day 12 and the average plasma concentration (C_{avg}) values on Days 1, 3, 6, 8, and 12 fit the sigmoid E_{max} model. At a C_{avg} of 1 μM sustained exposure in the 10 mg/mL group, the TGI was 88.3%, which was between that observed in the 10 mg/kg (TGI 82.1%) and 30 mg/kg (TGI 93.1%) PO dosed groups.

1.2.3 Safety Pharmacology, Toxicology, and Drug Absorption and Metabolism

Safety pharmacology studies evaluating the central nervous system (CNS) and cardiovascular effects of MLN8237 did not reveal significant adverse effects at the exposures anticipated to be required for human efficacy. MLN8237 has a low potential to

prolong the QT interval, based on its low in vitro activity against the potassium ion channel encoded by human ether-à-go-go related gene (hERG) and its lack of an effect on the QTcV interval in dogs. MLN8237 had minimal activity (< 40% inhibition) against all receptor-ligand interactions examined except GABAAα1 benzodiazepine. Although MLN8237 did elicit behavioral CNS effects attributed to its binding to the GABAAα1 benzodiazepine receptor, these effects occurred at dose levels which exceeded the repeat-administration daily PO maximum tolerated dose (MTD) of MLN8237.

The predominant effects of MLN8237 in Sprague-Dawley rats included peripheral blood cytopenias secondary to myelosuppression and increased mitotic figures/single cell necrosis (apoptosis) in tissues with a high basal cellular replication rate, consistent with the known mechanism of action of MLN8237. These effects were also the effects that predominated in repeat-dose studies in beagle dogs. CNS effects, consistent with the activity of MLN8237 at the GABAAα1 benzodiazepine receptor, were observed only in beagle dogs following a single, PO dose of 5 mg/kg, which exceeds the daily PO repeat-administration MTD in this species.

[redacted]

[redacted]

[redacted]

Detailed information regarding the nonclinical pharmacology and toxicology of MLN8237 may be found in the IB.

1.3 Clinical Experience With MLN8237

Clinical experience with MLN8237 includes phase 1 through 2 studies in both solid tumors and heme-lymphatic malignancies, described below. Two independent, phase 1 studies have identified the MTD to be 50 mg BID for 7 days, and this regimen was the recommended Phase 2 dose (RP2D) to be evaluated.[24, 25, 26] Single-agent, phase 2 experience has included 43 patients with aggressive lymphoma, enrolled to Study C14004, a study designed to evaluate clinical efficacy and safety of MLN8237 at 50 mg BID for 7 days in repeat, 21-day cycles (PIC formulation). Although final results are not complete, the available data from Study C14004 show promising evidence for clinical antitumor activity in this population, which includes patients with advanced DLBCL and other types of aggressive lymphoma, relapsed or refractory to the commonly available chemotherapy regimens. Among the first 9 patients with objective response reported by the investigators, 2 with DLBCL had sustained responses documented by computed tomography (CT) and positron emission tomography scans, and 1 patient with relapsed peripheral T-cell lymphoma was confirmed to have a CR. In this population, treatment over multiple cycles was generally tolerable, given dose reductions and supportive care, which sometimes included myeloid growth factors used in the treatment-free period, and some patients have remained on repeat cycles of MLN8237 treatment for periods of approximately 1 year. The manageable safety profile and tolerability, allowing repeat cycles of treatment for periods exceeding 1 to 2 years, were also reported from single-agent experience in patients with diverse forms of advanced malignancies. [24, 25, 26]

MLN8237 for clinical studies is being evaluated in 3 dosage formulations: PIC, ECT, and oral solution. The initial studies employed the PIC formulation, and more recent studies have evaluated safety, PK, relative bioavailability (in reference to the PIC), and antitumor activity after administration of the ECT formulation. Using the ECT formulation, the dose-escalation, phase 1 portion of Study C14007 has evaluated multiple dose levels from 10 to 60 mg BID for 7 days in repeat, 21-day cycles, and 50 mg BID has been identified as the MTD. Although Study C14007 continues enrollment, the available results from this and other studies support the performance characteristics of the ECT formulation.

Safety data from clinical studies were compiled (as of 29 March 2010), and descriptions of AEs can be reviewed in the IB. The predominant toxicities reflect the mechanism of action in proliferating tissues (bone marrow, gastrointestinal epithelium, and hair follicles). The suggested management of these toxicities is based on standard clinical paradigms for an antiproliferative chemotherapeutic agent. Using a treatment-free period for recovery between each cycle of drug administration, the clinical experience from multiple phase 1 through 2 studies indicate that major toxicities can be managed to allow repeat treatment cycles over periods extending beyond 12 to 24 months. Except for alopecia, the predominant toxicities are largely reversible, can be monitored by routine clinical examinations, and are manageable by dose reduction or supportive care.

MLN8237 is structurally related to the benzodiazepines (BZDs; eg, diazepam, lorazepam) and also has activity against the GABAAα1 receptor. BZD-like effects (eg, somnolence, confusion, memory loss) have been observed to be associated with the onset of maximal plasma concentration (eg, T_{max} [time to maximum plasma concentration]). CNS effects associated with peak plasma levels have been generally managed by administration of divided doses (eg, BID administration), although dose reductions have sometimes been required. While CNS effects attributed to MLN8237 were also generally reversible and manageable by dose delay or reduction, the causal relationship, and thus optimal approach to management, were sometimes confounded by diverse causes including, but not limited to, concomitant medications (eg, narcotic analgesics, anti-anxiety medications), comorbidities (eg, infection, anemia, electrolyte abnormalities), or progressive malignancy (eg, brain metastases).

The clinical experience with MLN8237 includes treatments with multiple doses and schedules as described in the IB. While most clinical experience supports a 7-day schedule followed by a 2-week, treatment-free period, clinical experience also supports treatment schedules with up to 14 days of drug administration in a 28-day cycle and up to 21 days of drug administration in a 35-day cycle.

1.3.1 Pharmacokinetics

Based on available preliminary PK data (as of 10 May 2010), upon oral administration as the PIC formulation to patients with advanced nonhematologic and hematologic malignancies, absorption of MLN8237 was fast, with peak plasma concentrations generally

achieved by 2 hours postdose. Steady-state plasma exposures of MLN8237 increased in an approximately dose-proportional manner over the range of 5 to 200 mg/day in patients with advanced solid tumors. PK variability was high following administration as PIC (%CV in steady-state, dose-normalized $AUC_{0\text{-}24hr}$ of 78%). Overall mean steady-state terminal half-life ($t_{1/2}$) following multiple dose administration in patients with nonhematologic malignancies was 23 hours. The overall mean peak/trough ratios were 2.5 and 5.2 for BID and QD dosing, respectively. The overall mean accumulation ratios were 2.9 and 1.8 for BID and QD dosing, respectively. PK steady-state conditions were approximately achieved by Day 7 following daily oral administration. The PK properties of MLN8237 in patients with hematologic malignancies were generally consistent with those observed in patients with nonhematologic malignancies. [redacted]

Based on limited preliminary data from patients treated with ECT up to 40 mg BID, the range of dose-normalized steady-state systemic exposure was within the overall range observed with PIC. Based on currently available data in 9 patients from the ongoing relative bioavailability study of the 25-mg oral solution versus 50-mg PIC, the dose-normalized geometric mean of MLN8237 C_{max} and $AUC_{0\text{-}inf}$ (area under the plasma concentration versus time curve zero to infinity) achieved following the administration of oral solution were approximately 40% and 20% higher than those observed from PIC, respectively.

Further details regarding the clinical PK of MLN8237 are provided in the IB.

1.4 Study Rationale

1.4.1 Rationale for Assessment of Treatment With MLN8237 and Weekly Paclitaxel

In experimental systems, modulation of Aurora kinase expression can block the metastatic phenotype and/or enhance the antitumor activity of standard chemotherapies. In OC models and clinical biopsies, Aurora kinase expression correlates with tumor cell proliferation, migration, and metastases.[12] Modulation of Aurora kinase activity in ovarian or other cancer models has been studied, including work with taxane combination treatments with tumor cells in vitro and in vivo xenografts.[13] These experiments generally demonstrated tumor cell apoptosis and delay or regression in xenograft models. Blockade of Aurora

kinase signaling enhanced the antitumor activity of chemotherapy, including taxanes in OC models.[15, 16, 27] At the molecular level, taxanes such as paclitaxel disrupt microtubule function resulting in tumor growth arrest. Inactivating mutations of Aurora A have resulted in disruption of the assembly of the mitotic spindle and inhibition of tumor cell proliferation, leading to accumulation of cells in the G2/M phase of the cell cycle and apoptosis. Studies have demonstrated that Aurora A activity can help to protect cells from taxane-induced apoptosis through activation of Akt.[15, 16] Targeting the mitotic apparatus by 2 complementary mechanisms of action by combining MLN8237 with a known active agent such as paclitaxel is a rational approach to treating patients in the setting of recurrent ovarian cancer.

Disease control and survival of patients with recurrent OC remains a major therapeutic challenge. New agents are required in order to improve the clinical outcome of patients with recurrent OC. Because large-scale clinical studies are often necessary to demonstrate the safety and effectiveness of these new agents, it is desirable to first evaluate some measure of relative effectiveness in a phase 2 study. Because of the poor prognosis of patients with recurrent OC and indications that an Aurora A-targeted agent may have clinically meaningful activity, a phase 2 study of the PO kinase inhibitor MLN8237 is warranted. In the subgroup with recurrent disease, weekly paclitaxel is an emerging standard of care, although the clinical outcomes have been generally modest. Safety findings from multiple studies indicate that neutropenia and other hematological toxicities are generally manageable by the use of a weekly schedule for paclitaxel administration. Because hematological toxicities also have been generally manageable, noncumulative, and reversible after single-agent treatment with MLN8237, the available data support the potential tolerability and clinical utility of a regimen that combines MLN8237 with weekly paclitaxel. Unlike the common toxicity of platinum combined with taxanes, MLN8237 has not led to major cumulative neurotoxicities, so the combination of weekly paclitaxel and MLN8237 is not predicted to amplify neurotoxicity, which can limit taxane delivery and its potential for long-term disease control.

The epidemiological, in vitro, and in vivo data support the investigation of Aurora A kinase inhibition for the treatment of patients with relapsed OC. MLN8237 may provide clinical utility in the population of patients who are candidates for treatment with chemotherapy in the setting of recurrent disease to enhance the activity of weekly paclitaxel. Thus, in this study, the relative antitumor activity of the combination of weekly paclitaxel and MLN8237 (investigational treatment arm, in which tolerable doses are to be first characterized in a

phase 1 portion) and the activity of single-agent weekly paclitaxel (active treatment comparator arm) will be evaluated.

Taken together, the data support clinical evaluation of MLN8237 targeting Aurora A kinase administered in combination with weekly paclitaxel for the management of patients with recurrent ovarian, fallopian tube, or primary peritoneal carcinoma.

1.4.2 Rationale for Pharmacokinetic Assessments

The Phase 1 portion of this study includes PK assessments for both paclitaxel and MLN8237, the intent of which is to evaluate the possibility of a PK interaction between these 2 agents. In vitro studies have shown that MLN8237 inhibited the Pgp-mediated efflux of paclitaxel in Caco-2 cells with an IC_{50} of 4.0 μM. Although, Pgp-mediated efflux is not the sole determinant of paclitaxel clearance (which is mediated primarily by hepatic metabolism by CYP 2C8 and 3A4 [paclitaxel product label]), it is possible that concomitant administration with MLN8237 may potentially result in an increase in paclitaxel exposure via inhibition of its efflux transport by Pgp.

Based on the considerations outlined above, the effect of concomitant administration of MLN8237 on the PK of paclitaxel will be characterized during the Phase 1 portion of the study. To enable this assessment, the PK of paclitaxel will be characterized during Cycle 1 on Day 1 (paclitaxel administered concomitantly with MLN8237) and Cycle 2 on Day 1 (paclitaxel administered alone). To obtain PK measurements for paclitaxel alone at Cycle 2, Day 1, without the effect of concomitantly administered MLN8237, dosing with MLN8237 will be initiated in Cycle 2 on Day 8 and administered on Days 9, 10, 15, 16, and 17 of Cycle 2. Additionally, MLN8237 PK will be assessed during Cycle 1, on Days 1 and 3.

During the Phase 2 portion of this study, limited (sparse) PK sampling will be performed for MLN8237 only, to contribute to population PK analyses of MLN8237 and to contribute to potential exploratory analyses of exposure-response relationships for efficacy or safety endpoints, or both.

1.4.3 Rationale for Assessment of Biomarkers in Archived Tumor Tissue

Aurora A amplification or increased Aurora A messenger RNA (mRNA) levels have been associated with kinase overexpression[(28)] and tumorigenesis[(29)] in a variety of tumors. Recent gene expression analyses across various cancers (breast and lung cancer, medulloblastoma, glioma, mesothelioma and lymphoma) also link Aurora A overexpression

to aneuploidy and poor outcome.[(30)] [redacted] This will be evaluated in the banked tumor specimen. The tumor tissue may have been obtained at the time of the patient's initial tumor diagnosis or from a subsequent procedure conducted as part of the patient's standard care. If a better understanding of the role of Aurora A kinase in cancer suggests that there may be additional biomarkers of response to treatment with MLN8237, then the tumor tissue may be evaluated for these biomarkers as well. While it is unlikely that the number of patients treated in this study will provide data sufficient to explore the potential relationship between markers such as Aurora A kinase levels and response to MLN8237 treatment, the combination of the data from this study and future studies of MLN8237 may permit such an exploratory analysis.

1.4.4 Rationale for Genotyping

[redacted]

1.4.5 Rationale for Serum Assessment of Tumor Response

[redacted] Furthermore, there is increasing evidence that these markers of tumor cell death may be correlated to response in some patients with epithelial-derived cancers treated with chemotherapy.[37, 38] Therefore, patient blood samples will be collected and assessed for markers of response to treatment in serum, such as but not limited to [redacted]

1.5 Potential Risks and Benefits

The safety risks of MLN8237 treatment are described in the IB. In summary, they include toxicities to proliferating tissues resulting in myelosuppression, mucositis and other gastrointestinal toxicities, and alopecia. In addition, MLN8237 is associated with benzodiazepine-like effects such as sedation and confusion. Clinical safety data include experience from patients who received multiple cycles followed by treatment-free periods between each cycle and from patients who reduced or discontinued treatment. Based on the available clinical data, drug abuse, dependency, and withdrawal were not observed.

While these toxicities are potentially associated with risk or discomfort to the patient, they are anticipated to be reversible. There is the possibility that other toxicities, not previously observed in patients or predicted from evaluation of MLN8237 in rats and dogs and from ongoing studies in humans, may be observed. MLN8237 has a very low potential to prolong the QT interval in vivo based upon its extremely weak in vitro binding to hERG (IC_{50} and K_i both > 100 μM).

This study will be conducted in compliance with the protocol, Good Clinical Practice (GCP), applicable regulatory requirements, and International Conference on Harmonisation (ICH) guidelines.

2. STUDY OBJECTIVES

2.1 Phase 1 Objectives

2.1.1 Phase 1: Primary Objectives

The primary objectives of the Phase 1 portion of this study are as follows:

- To assess the safety and tolerability of MLN8237 plus weekly paclitaxel
- To determine the recommended dose and schedule of MLN8237 and dose of paclitaxel to be used as the combination treatment arm in Phase 2

2.1.2 Phase 1: Secondary Objectives

The secondary objectives of the Phase 1 portion of this study are as follows:

- To characterize the effect of concomitant administration of MLN8237 on the PK of paclitaxel
- To characterize the PK of MLN8237 administered concomitantly with weekly paclitaxel
- To assess the best overall combined response in patients with recurrent OC or breast cancer

2.1.3 Phase 1: Exploratory Objective

The exploratory objective of the Phase 1 portion of this study is as follows:

- [redacted]
- [redacted]

2.2 Phase 2 Objectives

2.2.1 Phase 2: Primary Objective

The primary objective of the Phase 2 portion of this study is as follows:

- To assess PFS after treatment with the combination of MLN8237 and weekly paclitaxel or weekly paclitaxel alone in patients with recurrent OC, and to compare PFS of the populations enrolled to these 2 treatment arms

2.2.2 Phase 2: Secondary Objectives

The secondary objectives of the Phase 2 portion of this study are as follows:

- To estimate the overall response rate (ORR), DOR, TTP, and OS associated with MLN8237 plus weekly paclitaxel and weekly paclitaxel alone in patients with recurrent OC
- To assess the safety and tolerability of MLN8237 plus weekly paclitaxel

2.2.3 Phase 2: Exploratory Objectives

The exploratory objectives of the Phase 2 portion of this study are as follows:

- [redacted]
- [redacted]
- [redacted]

3. STUDY ENDPOINTS

3.1 Phase 1 Endpoints

3.1.1 Phase 1: Primary Endpoints

The primary endpoints for the Phase 1 portion of this study include:

- AEs, serious adverse events (SAEs), assessments of clinical and laboratory values, and vital sign measurements with a special attention to hypersensitivity reactions and neurotoxicity from the combination
- Safe doses of MLN8237 and paclitaxel when given in combination

3.1.2 Phase 1: Secondary Endpoints

The secondary endpoints for the Phase 1 portion of this study include:

- Best overall combined response
- PK parameters of MLN8237, including but not limited to, maximum plasma concentration (C_{max}), T_{max}, and area under the plasma concentration versus time curve zero to the end of the dosing interval ($AUC_{0-\tau}$)
- PK parameters of paclitaxel, including but not limited to, C_{max}, $AUC_{0-tlast}$, $AUC_{0-\infty}$, and $t_{½}$, when administered alone and during concomitant administration of MLN8237

3.1.3 Phase 1: Exploratory Endpoints

The exploratory endpoint for the Phase 1 portion of this study includes:

- [redacted]
- [redacted]

3.2 Phase 2 Endpoints

3.2.1 Phase 2: Primary Endpoint

The primary endpoint for the Phase 2 portion of this study includes:

- PFS

3.2.2 Phase 2: Secondary Endpoints

The secondary endpoints for the Phase 2 portion of this study include:

- ORR, DOR, TTP, and OS
- AEs, SAEs, assessments of clinical laboratory values, and vital signs measurements

3.2.3 Phase 2: Exploratory Endpoints

The exploratory endpoints for the Phase 2 portion of this study include:

- [redacted]
- [redacted]
- [redacted]

4. STUDY DESIGN

4.1 Overview of Study Design

This is an open-label, multicenter study with a nonrandomized, Phase 1 portion and an open-label, randomized, Phase 2 portion evaluating MLN8237 in combination with weekly paclitaxel in adult female patients with advanced breast cancer (Phase 1 only) and recurrent OC (both Phase 1 and Phase 2). During the lead-in Phase 1 portion of the study, the MTD of MLN8237 combined with paclitaxel will be determined and the PK characterized in eligible patients. The Phase 1 portion will include female patients with adenocarcinoma of the breast or patients with recurrent OC who have recurrent disease documented within 12 months after discontinuation of platinum therapy or patients who experience persistent or recurrent disease during platinum therapy. When a RP2D is determined in the Phase 1 portion of the study, patients with recurrent OC who have a treatment-free interval ≤ 12 months after platinum therapy or patients who experience persistent or recurrent disease during platinum therapy and who are candidates for treatment with weekly paclitaxel will be enrolled in the Phase 2 portion. These latter patients will be randomized to study treatment with either the combination of MLN8237 plus weekly paclitaxel or single-agent weekly paclitaxel in the Phase 2 portion.

The RP2D of MLN8237 combined with weekly paclitaxel will be determined in Phase 1 for further evaluation in the Phase 2 portion of the study based on composite clinical data, including PK, safety, and tolerability over multiple treatment cycles. The RP2D will be determined upon review of the available clinical data by the sponsor, a Safety Monitoring Committee (SMC), and consultation with the Phase 1 investigators. This review to determine the RP2D will be summarized, along with available supporting data from the Phase 1 part of the study, and provided to the Phase 2 investigators prior to initiation of enrollment to that part of the study. The Phase 1 dose escalation cohorts include the option to reduce the dose of paclitaxel (eg, from a starting dose of 80 mg/m^2 reduced to 70 or 60 mg/m^2) if elevated paclitaxel exposures or excess toxicities are observed when administered in combination with MLN8237. If a combination regimen is investigated in the Phase 1 portion with a starting dose of paclitaxel below 80 mg/m^2 and the individual patient tolerates the starting doses without severe (CTC Grade 3 or higher) toxicity, the paclitaxel dose can be escalated after completion of Cycle 2 up to a maximum of 80 mg/m^2 and safety monitoring will continue as required by the Schedule of Events. Upon determination of the RP2D regimen to be used in Phase 2, the sponsor and Phase 1 investigators will review safety and tolerability of patients who received paclitaxel escalated

from a reduced starting dose up to 80 mg/m^2; if the Phase 1 data indicate that such paclitaxel escalation was generally tolerable, this option will be continued in Phase 2 for individual patients enrolled to the combination arm. After paclitaxel dose escalation, management of toxicities by dose reduction, delays, or supportive care continue to follow protocol guidelines.

Determination of the RP2D will include analysis of tolerance by a minimum of 6 patients with no more than 1 out of 6 experiencing DLT in the first cycle. Determination of RP2D will include patients treated with the combination, for 2 or more cycles without evidence for major cumulative toxicities such as progressive Grade 4 thrombocytopenia or complicated/prolonged Grade 4 neutropenia. In addition, determination of the RP2D will include analysis of patients who received the planned full doses, to be recommended for Phase 2, over 2 or more cycles, and without a requirement for myeloid growth factors during the first 2 cycles. At the RP2D, if a minimum of 3 patients, treated without myeloid growth factors, have not completed 2 or more cycles due to reasons other than safety (eg, PD), then they will be replaced so that at least 3 patients have received and tolerated the RP2D for 2 or more cycles (without a requirement for myeloid growth factors during the first 2 cycles) in support of this dose and schedule to be used in Phase 2. In the Phase 1 cohort that determines the RP2D, a minimum of 4 patients will have the diagnosis of recurrent OC.

An interim analysis will be performed after the first 72 evaluable patients in the Phase 2 portion have either completed a minimum of 2 cycles of therapy or have discontinued study treatment. The interim analysis will be based on the clinical benefit rate (CBR), which includes the best overall combined response and SD as shown in Table 8-1, and a patient must have at least 2 continuous assessments of SD to be counted. The study will be terminated if the CBR of the MLN8237 plus weekly paclitaxel arm is at least 10% lower than that of the weekly paclitaxel-alone arm.

The final analysis will be performed after a total of 110 PFS events (PD or death) have occurred in Phase 2, and PFS between the 2 treatment arms will be compared.

Patients cannot have received antineoplastic therapy or radiotherapy within the 3 weeks before enrollment (2 weeks for regimens with recovery expected within 7 to 14 days). Patients with recurrent OC must have PD per RECIST, version 1.1,[39] (Section 15.3) or modified Gynecologic Cancer Intergroup (GCIG) CA-125 criteria[40] (Section 15.4) and have experienced recurrence ≤12 months after discontinuation of a platinum-based chemotherapy regimen. Patients with CA-125 marker-only evidence for PD are included

only if there is also radiological evidence of disease. In the Phase 1 portion, patients with breast cancer must have measurable disease.

During the Phase 1 portion of the study, the starting dosage of MLN8237 will be 10 mg PO BID for 3 days followed by a 4-day rest period (3 days on/4 days off) repeated weekly for 3 weeks in a 28-day cycle. In new cohorts, dose escalation for the Phase 1 study may proceed until the MTD is determined (see Table 6-1). The dose of MLN8237 will be doubled in the next 2 successive cohorts if a first-cycle DLT is not observed; after reaching 40 mg BID or if 1 patient experiences DLT in earlier cohorts, subsequent increases in MLN8237 dose will be approximately 25%. Doses at each level will be adjusted to accommodate the available tablet strengths. Doses can be reduced in subsequent treatment cycles as described in Section 6.5. One of the objectives of the Phase 1 portion of this study is to evaluate the effect of concomitant administration of MLN8237 on paclitaxel PK. Therefore, MLN8237 will be administered beginning on Day 8 in Cycle 2 in order that paclitaxel PK following the Day 1 dose can be evaluated without concomitantly administered MLN8237 to serve as reference for comparison to paclitaxel PK, evaluated at Cycle 1, Day 1 in the presence of MLN8237. For Cycle 2 only, during the Phase 1 portion, patients will receive 12 doses of MLN8237, administered BID on Days 8, 9, 10, 15, 16, and 17.

During the Phase 2 portion, the MLN8237 plus weekly paclitaxel regimen will be determined by the Phase 1 portion; patients will be assigned randomly in a 1:1 ratio to treatment with MLN8237 plus weekly paclitaxel or weekly paclitaxel alone. Paclitaxel will be administered as an intravenous (IV) infusion on Days 1, 8, and 15 of each 28-day cycle. Patients randomized to treatment with single-agent paclitaxel will receive a planned starting dose of 80 mg/m^2, which may be reduced or delayed if required to manage toxicities according to rules described for the combination of MLN8237 plus paclitaxel (see Section 6.5). The planned combination therapy schedule includes 9 days (18 doses) of MLN8237 drug administration overlapping weekly paclitaxel administration (Days 1, 8, and 15) in each 28-day cycle.

Individual dose reductions will be made on the basis of the AEs observed as defined by protocol. Patients may continue to receive repeated cycles of treatment as part of this protocol for up to 24 months, until there is evidence of PD or unacceptable treatment-related toxicity, or until another antineoplastic therapy is started. Treatment with MLN8237 and paclitaxel may be continued beyond 24 months if after discussion between the investigator and the sponsor it is determined that a patient would clearly derive benefit from continued

therapy. MLN8237 treatment may be continued in this or in another extension or rollover study, if available, upon request by the investigator and agreement by the medical monitor. Patients who discontinue their participation in the study for any reason may continue paclitaxel or other taxane therapy at the discretion of the treating physician. In the Phase 1 portion, the strategy for de-escalation or evaluation of a reduced MLN8237 dosing schedule, if required, will be determined by the sponsor and investigators upon review of PK and clinical data from the initial cohorts, including the option to reduce the dose of paclitaxel if elevated exposures or excess toxicity are observed when administered in combination with MLN8237. If the lower dose of paclitaxel is tolerated, re-escalation of the MLN8237 and/orpaclitaxel dose will be determined by the sponsor and investigators upon review of PK and safety experience from initial cohorts.

Extent of disease evaluations, based on radiographic procedures, such as CT with IV contrast or magnetic resonance imaging (MRI) scans for sites of disease not adequately imaged by CT, nuclear medicine scans as appropriate (eg, known or suspected bone metastases), and findings on physical examination, will be obtained at the end of every second treatment cycle (ie, approximately every 8 weeks, within the week prior to the start of a new cycle). Scans are required at the End of Treatment (EOT)/End of Study (EOS) visit only if PD has not been documented previously or it has been 8 weeks or longer since the previous evaluation. Patients (enrolled in both Phase 1 and Phase 2) followed off treatment will be evaluated every 8 weeks until the occurrence of 110 PFS events (PD or death) in Phase 2. CA-125 levels will be obtained according to standard of care within 9 days of screening and sufficient time after prior therapy, at the end of every treatment cycle, within the week prior to the start of a new cycle, at the EOT visit, and during follow-up visits for patients who are followed off treatment. Confirmation of response by CA-125 levels must be obtained 28 days after the previous sample.

Response will be based on either RECIST, version 1.1, for measurable neoplastic disease (Section 15.3) or on changes in CA-125 based on the CA-125 response definition for patients with CA-125 levels in excess of 70 units/mL according to modified GCIG criteria (Section 15.4). PD will be based on RECIST, version 1.1, for measurable neoplastic disease or on CA-125 criteria with elevated (> 70 units/mL) levels on 2 occasions and other criteria as described in the modified GCIG criteria (see Section 7.4.12).[39, 40, 41] CT or MRI scans necessary to document response based on RECIST will be transferred to a compact disk (CD) or digital video disk and provided to the sponsor for subsequent review.

Toxicity will be evaluated according to National Cancer Institute Common Terminology Criteria for Adverse Events (NCI CTCAE), version 4.02, effective as of 01 October 2009.[42] DLTs are defined in Section 6.3.

AEs will be assessed, and laboratory values, vital signs, and electrocardiograms (ECGs) will be obtained to evaluate the safety and tolerability of the combination of MLN8237 plus weekly paclitaxel.

4.2 Number of Patients

Approximately 172 total patients (approximately 36 patients in Phase 1 and approximately 136 patients in Phase 2) will be enrolled in this study. The Phase 1 dose escalation will be conducted at approximately 3 study centers. Approximately 136 patients in Phase 2 will be randomized in a 1:1 ratio to either MLN8237 combined with paclitaxel or single-agent paclitaxel at approximately 40 participating study centers.

In the Phase 1 portion of the study, the initial cohort will include a minimum of 3 patients; planned cohort sizes in Phase 1 (3 to 6 patients) may be expanded with written agreement by the sponsor if required due to logistic considerations. The RP2D cohort will include a minimum of 12 patients (inclusive of the expansion phase) who complete protocol-specified dosing and PK measurements in the first 2 cycles of treatment at the RP2D. The minimum of 12 patients planned for enrollment at the RP2D include 6 for determination of the dose and schedule used in the Phase 2 portion and an additional minimum of 6 in an expansion cohort to refine understanding about the relationships between dose and PK and safety. Planned cohort sizes in Phase 1 (3 to 6 patients) may be expanded with written agreement by the sponsor if required due to logistic considerations.

For determination of the MTD in Phase 1, patients will be replaced in a cohort if withdrawn from treatment during Cycle 1 for reasons other than DLT, including the following situations: failure to complete 1) at least 80% of the planned doses of MLN8237; or 2) at least 2 of the planned 3 doses of paclitaxel; or 3) the required safety evaluations scheduled in Cycle 1 to determine DLT.

4.3 Duration of Study

Patients may remain in the study until the development of progressive neoplastic disease, the development of unacceptable toxicity, or fulfillment of any of the criteria for withdrawal from study as described in Section 7.7 of the protocol. The maximum duration of therapy

will be 24 months, unless after discussion between the investigator and sponsor it is determined that a patient would derive benefit from continued therapy beyond 24 months. Patients (enrolled in both Phase 1 and Phase 2) who do not experience PD will be followed off treatment once every 8 weeks until the occurrence of 110 PFS events (PD or death) in Phase 2.

The duration of the Phase 1 portion of the study is approximately 34 months, including an enrollment period of approximately 10 months and a treatment period of approximately 24 months. The duration of the Phase 2 portion of the study is approximately 36 months, including an enrollment period of approximately 12 months and a treatment period of approximately 24 months.

The final analyses for the clinical study report will be conducted after the occurrence of 110 PFS events in the patients enrolled in the Phase 2 portion of the study.

5. STUDY POPULATION

5.1 Inclusion Criteria

Each patient must meet all of the following inclusion criteria to be enrolled in the study:

1. Female patients 18 years or older.
2. Previously treated, metastatic or locally recurrent malignancy with 1 of the 2 following diagnoses, which has been confirmed histologically or cytologically: a) adenocarcinoma of the breast (Phase 1 only); or b) OC, defined to include recurrent epithelial ovarian, fallopian tube, or primary peritoneal cancer (Phases 1 and 2).
3. Patients with breast cancer must have received prior treatment with at least 1 but no more than 4 prior chemotherapy regimens for locally recurrent or metastatic disease, not including regimens received in the neoadjuvant and/or adjuvant setting.
4. In the Phase 1 portion of the study, patients with breast cancer must have measurable disease (per RECIST, version 1.1; see Section 15.3).
5. No antineoplastic therapy (eg, drugs, biologicals, monoclonal antibodies, etc) or radiotherapy within the 3 weeks before enrollment (2 weeks for regimens with recovery expected within 7 to 14 days). The patient must have recovered

(ie, ≤ Grade 1 toxicity or patient's baseline status, except alopecia) from all treatment-related toxicities and must have evidence of PD or persistent disease. The patient must have experienced no more than Grade 2 non-hematologic toxicity attributed to a prior taxane therapy. No hormonal therapy except hormone replacement therapy or birth control medications.

6. Eastern Cooperative Oncology Group (ECOG) performance status (PS) of 0 or 1 (refer to Section 15.1).
7. Adequate bone marrow function as defined by:
   - Absolute neutrophil count (ANC) ≥ 1,500 cells/mm^3 (without need for growth factor support)
   - Platelet count ≥ 100,000 cells/mm^3 (without need for transfusion or growth factor support)
   - Hemoglobin level ≥ 9 g/dL
8. Adequate liver function as defined by:
   - Bilirubin < 1.5 times the upper limit of normal (ULN)
   - Alanine aminotransferase (ALT) and aspartate aminotransferase (AST) ≤ 2.5 x ULN (≤ 5 x ULN if due to liver metastases)
   - serum albumin greater than the lower limit of normal (LLN)
9. Adequate renal function as defined by:
   - Creatinine clearance ≥ 30 mL/minute (can be calculated using serum creatinine value; see Section 15.2)
10. Patients who:
    - Are postmenopausal for at least 1 year before the screening visit, OR
    - Are surgically sterile, OR
    - If they are of childbearing potential, agree to practice 2 effective methods of contraception, at the same time, from the time of signing the informed consent form (ICF) through 30 days after the last dose of study drug, or agree to completely abstain from heterosexual intercourse
11. The patient or the patient's legal representative is able to provide written informed consent. Voluntary written consent must be given before performance of any

study-related procedure not part of standard medical care, with the understanding that consent may be withdrawn by the patient at any time without prejudice to future medical care.

12. Willing and able to comply with scheduled visits, treatment plan, laboratory tests, and other study procedures.
13. Suitable venous access for the study-required blood sampling (ie, including PK sampling).

Specific Inclusion Criteria for Patients with Recurrent Ovarian, Fallopian Tube, or Peritoneal Cancer

14. Prior treatments must have included a platinum and a taxane. The most recent treatment regimen (administered prior to enrollment) need not be a platinum-containing or a taxane-containing regimen.
    - Recurrent disease must be documented within 12 months after discontinuation of platinum therapy, based on RECIST or GCIG CA-125 criteria for disease progression described in Section 5.1.16 below.
    - Patients who previously received weekly taxane are potentially eligible, provided that they did not progress during therapy or within 3 months of completing therapy.
    - Patients with platinum-refractory disease, as defined by progression during primary or subsequent platinum-based therapy or persistent radiographic disease after primary or subsequent platinum-based therapy, will be included.
15. Patients must have measurable disease in target lesions or assessable disease, and disease progression per RECIST version 1.1 (Section 15.3) or modified GCIG CA-125 criteria (Section 15.4).

Assessable disease is defined by CA-125 criteria as follows: minimum CA-125 level at the time of entry must have been more than 70 units/mL. This CA-125 level must have at least doubled from baseline to demonstrate evidence of PD from a previous treatment (with the value having been confirmed by at least 2 separate blood samples obtained ≥ 4 weeks apart or other clinical evidence of PD). Patients with CA-125 marker evidence for PD are included only if there is also clinical evidence of PD, eg, CT evidence for PD that is not measurable by RECIST. Radiological evidence of disease does not need to be measurable. Radiological abnormality, persistent after prior therapy, should be confirmed by biopsy to be a viable malignancy in the absence of other clinical or radiological evidence for PD.

5.2 Exclusion Criteria

Patients meeting any of the following exclusion criteria are not to be enrolled in the study:

1. Prior treatment with an Aurora A-targeted agent (including MLN8237).
2. Treatment with clinically significant enzyme inducers within 14 days prior to the first dose of MLN8237 and during the study. Major prohibited enzyme inducers include the following:
    - phenytoin
    - carbamazepine
    - phenobarbital
    - rifampin
    - rifabutin
    - rifapentine
    - St. John's wort
3. Treatment with more than 4 cytotoxic chemotherapy treatment regimens in the metastatic setting. A cytotoxic agent is defined as any agent that targets the genetic, mitotic, and/or metabolic pathways of dividing cells, resulting in toxicity to the bone marrow and/or gastrointestinal mucosa. A chemotherapy regimen is defined as 1 or more agents used continuously or discontinuously (ie, allowing a break or chemotherapy holiday) without the addition of a new agent. A regimen administered pre-operatively and continued after surgery is considered a single regimen. Hormonal therapy is not considered to be a chemotherapy regimen.

- The most recent treatment regimen need not be a platinum-containing or a taxane-containing regimen.
- Prior therapy cannot include more than 2 prior taxane-containing regimens.

4. Current use of tamoxifen, thalidomide, or any agent used as maintenance or consolidation therapy for OC.
5. Known hypersensitivity to Cremophor® EL, paclitaxel, or its components.
6. Prior history of ≥ Grade 2 neurotoxicity or any toxicity requiring discontinuation from taxane chemotherapy that is not resolved to ≤ Grade 1.
7. Any comorbid condition or unresolved toxicity that would preclude administration of weekly paclitaxel.
8. Diagnosis of primary CNS malignancy or carcinomatous meningitis.
9. Patient has symptomatic brain metastasis. Patients with brain metastases must:
   - Have stable neurologic status following local therapy (surgery or radiation) for at least 2 weeks after completion of the definitive therapy; and
   - Be without neurologic dysfunction that would confound the evaluation of neurologic and other AEs.
10. Inability to swallow PO administered medications or to maintain a fast as required before and after MLN8237 administration.
11. History of hemorrhagic or thrombotic cerebrovascular event in the past 12 months.
12. Surgery within 3 weeks before study enrollment and not fully recovered to baseline or to a stable clinical status.
13. Diagnosis of or treated for another malignancy within 2 years before the first dose or previously diagnosed with another malignancy and have any evidence of residual disease. Patients with nonmelanoma skin cancer or carcinoma in situ of any type, including patients with Stage IA or IB endometrioid carcinomas, are not excluded if they have undergone complete resection.
14. Patients who are lactating or have a positive serum pregnancy test during the screening period.
15. Other severe acute or chronic medical or psychiatric condition, including uncontrolled diabetes, malabsorption, resection of the pancreas or upper small bowel,

requirement for pancreatic enzymes, any condition that would modify small bowel absorption of oral medications, or laboratory abnormality that may increase the risk associated with study participation or investigational product administration or may interfere with the interpretation of study results and, in the judgment of the investigator, would make the patient inappropriate for enrollment in this study.

16. Requirement for constant administration of proton pump inhibitor, H2 antagonist, or pancreatic enzymes. Intermittent uses of antacids or H2 antagonists are allowed as described in Section 6.6.
17. Treatment with any investigational products within 21 days before the first dose of study drug.
18. Prior allogeneic bone marrow or organ transplantation.
19. Systemic infection requiring IV antibiotic therapy within 14 days preceding the first dose of study drug, or other severe infection.
20. Known history of human immunodeficiency virus (HIV) infection, hepatitis B, or hepatitis C. Testing is not required in the absence of clinical findings or suspicion.
21. Radiotherapy to ≥ 25% of bone marrow (Section 15.5), or whole pelvic radiotherapy.

6. STUDY DRUG

6.1 Study Drug Administration

During Phase 1, MLN8237 will be administered PO at a starting dosage of 10 mg BID for 3 days followed by a 4-day rest period (3 days on/4 days off) repeated weekly for 3 weeks (see Table 6-1) combined with a starting dose of paclitaxel of 80 mg/m^2 on Days 1, 8, and 15 (Section 6.2.1) in each 28-day treatment cycle. In new cohorts, dose escalation may proceed until the MTD is determined (see Table 6-1). The dose of MLN8237 will be doubled in the next 2 successive cohorts if a first-cycle DLT is not observed; after reaching 40 mg BID or if 1 patient experiences DLT in earlier cohorts, subsequent increases in MLN8237 dose will be approximately 25%. Doses at each level will be adjusted to accommodate the available ECT formulation strengths.

MLN8237 dosing during Cycle 1 will begin at Cycle 1, Day 1, the same day as the first dose of paclitaxel; MLN8237 will be administered 1 hour before the start of the paclitaxel infusion. During Phase 1, Cycle 2 only, MLN8237 dosing will begin on Day 8 and continue for a total of 12 doses (on Days 8, 9, 10, 15, 16, and 17) to permit PK assessment of paclitaxel alone out to 48 hours (see Section 7.4.19). However, the MLN8237 schedule may be modified (eg, shortened by eliminating the MLN8237 dose administered on or after Day 8) based on safety experience from early cohorts during the dose escalation part of the study. MLN8237 dosing in all subsequent cycles (Cycle 3 and beyond) will begin on Day 1. The dose of MLN8237 in the Phase 2 portion of the study will be determined by the Phase 1 portion, and dosing will begin on Day 1 in all treatment cycles.

MLN8237 will be administered on an empty stomach with the patient remaining NPO (nothing by mouth), except for water and prescribed medications, for 2 hours before and 1 hour after each dose. Patients will be instructed to take each PO dose of MLN8237 with 8 ounces (1 cup, 240 mL) of water. The 2 daily doses must be taken at least 6 hours apart.

MLN8237 will be supplied as 10 or 50 mg ECT, with the dose strength expressed as milligrams of active drug (free acid); other strengths may be supplied depending on the observed MTD. All tablets are to be ingested whole; patients who have difficulty swallowing tablets will be excluded from the study. Antiemetogenic agents may be administered at the discretion of the investigator, but prophylactic antiemetic agents should not be administered in the first cycle of treatment if nausea or vomiting is not observed. Although not prohibited, the use of benzodiazepines for the prophylaxis or treatment of

nausea or vomiting is discouraged because of the potential benzodiazepine-like effects of MLN8237.

Study drug will be administered only to eligible patients under the supervision of the investigator or identified subinvestigator(s).

6.2 Reference/Control Therapy

6.2.1 Paclitaxel

During the Phase 1 portion of the study, paclitaxel will be administered as an IV infusion with a starting dose of 80 mg/m^2 on Days 1, 8, and 15. However, the paclitaxel starting dose may be decreased (eg, to 70 or 60 mg/m^2) based on safety experience and determination of the MTD and RP2D during the dose escalation part of the study. If paclitaxel at a reduced dose is well tolerated when administered in combination with MLN8237, re-esclation to 80 mg/m^2 paclitaxel may be considered. The paclitaxel infusion time is 1 hour; the infusion time may be modified if required after review and agreement by the medical monitor. During the Phase 2 portion, the MLN8237 plus weekly paclitaxel regimen will be determined by the Phase 1 portion. Refer to the paclitaxel product label for further details regarding paclitaxel administration.

6.2.2 Premedication for Paclitaxel-Associated Hypersensitivity or Other Acute Reactions

Premedication to prevent paclitaxel-associated (hypersensitivity or other) reactions is recommended according to institutional guidelines or local practices. Premedications that may be used in this setting can include: corticosteroid (eg, dexamethasone, as a 20-mg single dose, which can be reduced with subsequent paclitaxel cycles); diphenhydramine (maximum dose of 25 mg); a 5-hydroxytryptamine 3 [5-HT$_3$] serotonin receptor antagonist antiemetic administered at its labeled dose (but prophylactic antiemetic agents should not be administered in the first cycle of treatment if nausea or vomiting is not observed). Benzodiazepines are to be avoided. Brief administration of histamine-2 antagonists (such as a single dose of cimetidine or ranitidine) is allowed if required on the day of paclitaxel administration, but prolonged administration of a histamine-2 antagonist (or any other agent that can alter stomach pH or drug absorption) is to be avoided (see Section 6.6). On days when both MLN8237 and paclitaxel are administered on the same morning, the MLN8237 should be administered 1 hour before the start of the paclitaxel infusion. Modifications to

paclitaxel administration or to the premedications are allowed upon agreement by the medical monitor and will be documented in the electronic case report form (eCRF).

6.3 Definitions of Dose-Limiting Toxicity

Toxicity will be evaluated according to the NCI CTCAE, version 4.02, effective as of 01 October 2009. These criteria are provided in the Study Manual and are available online at http://ctep.cancer.gov/reporting/ctc.html.

DLT will be defined as any of the following events that are considered by the investigator to be related to therapy with MLN8237 plus weekly paclitaxel (Phase 1 and 2 portions) or to weekly paclitaxel (Phase 2 portion). See also Section 6.5.3 for toxicities considered to be DLTs if they occur during the dosing period in the first cycle.

1. Grade 4 neutropenia (ANC < 500 cells/mm^3) lasting more than 7 consecutive days.
2. Grade 4 neutropenia associated with coincident fever (where fever is defined as an oral temperature ≥ 38.5°C).
3. Grade 4 thrombocytopenia lasting more than 7 consecutive days.
4. A platelet count < 10,000/mm^3 at any time.
5. Grade 3 thrombocytopenia with clinically significant bleeding.
6. ≥ Grade 3 nonhematological toxicity, with the following exceptions:
   - Grade 3 or greater nausea or emesis, or both, that occurs in the absence of optimal antiemetic therapy (5-HT$_3$ serotonin receptor antagonist).
   - Grade 3 or greater diarrhea that occurs in the absence of optimal supportive therapy with loperamide.
   - Grade 3 fatigue that lasts less than 1 week.
   - Grade 3 nonhematological toxicity that can be controlled to Grade 2 or less with appropriate treatment (eg, Grade 3 hypertension will be considered a DLT only if the hypertension is unmanageable by standard approved pharmacological agents or if symptomatic sequelae are identified despite appropriate medical intervention).
7. ≥ Grade 2 nonhematological toxicities that are considered by the investigator to be related to study drug and in the opinion of the investigator require dose reduction
8. Delay in the initiation of the subsequent cycle of therapy by more than 7 days due to

treatment-related toxicity.

Although DLTs may occur at any point during treatment, only DLTs occurring during Cycle 1 of treatment during the Phase 1 portion of this study will necessarily influence decisions regarding dose escalation, expansion of a dose level, or evaluation of intermediate dose levels or alternative schedules.

Patients experiencing DLTs in Cycle 1 may continue in the study provided they are deriving clinical benefit but will have doses of MLN8237 and/or paclitaxel reduced as appropriate.

6.4 Dose Escalation Rules

Accrual and treatment will proceed according to the following rules:

- In the Phase 1 portion of the study, the initial cohort will include a minimum of 3 patients. Planned cohort sizes (3 to 6 patients) may be expanded with written agreement by the sponsor if required due to logistical considerations. For purposes of the MTD estimation based on the incidence of DLT, only data for 6 patients from Cycle 1 will be used. The RP2D of MLN8237 combined with weekly paclitaxel will be determined for further evaluation in the Phase 2 portion of the study based on composite clinical data, including PK, safety, and tolerability over multiple treatment cycles. Moreover, determination of the RP2D will include analysis of tolerance by patients, treated with the combination, for 2 or more cycles without evidence of major cumulative toxicities such as progressive Grade 4 thrombocytopenia or complicated/prolonged Grade 4 neutropenia. In addition, determination of the RP2D will include analysis of patients who received the planned full doses, to be recommended for Phase 2, over 2 or more cycles, and without a requirement for myeloid growth factors during the first 2 cycles. At the RP2D, if a minimum of 3 patients have not completed 2 or more cycles due to reasons (eg, PD) other than safety, then they will be replaced so that a minimum of 3 patients have received and tolerated the RP2D for 2 or more cycles (without a requirement for myeloid growth factors during the first 2 cycles) in support of this dose and schedule to be used in the Phase 2 portion. The RP2D cohort will include a minimum of 12 patients (inclusive of the expansion phase) who complete protocol-specified dosing and PK measurements in the first 2 cycles of treatment at the RP2D. The 12 patients planned for enrollment at the RP2D include 6 for determination of the dose and schedule used in the Phase 2 portion and an additional 6 in an expansion cohort to refine

understanding about the relationship between dose and PK and safety. A minimum of 4 patients with recurrent OC are to be included in the RP2D cohort.

- The initial cohort will receive 80 mg/m^2 paclitaxel by 60-minute IV infusion on Days 1, 8, and 15 followed by 2 weeks of rest from paclitaxel treatment.
- The dosage of MLN8237 in the initial cohort will be 10 mg PO BID, each dose taken with 240 mL water after a 2-hour fast. During the Phase 1 portion, the first dose of MLN8237 in Cycle 1 will be administered on Day 1 (ie, Cycle 1, Day 1) and dosing (3 days on/4 days off) will be repeated weekly for 3 weeks in each 28-day cycle, for a total of 18 doses. During Cycle 2, the first dose of MLN8237 will be administered on Day 8 (ie, Cycle 2, Day 8) and continue for a total of 12 doses (Days 9, 10, 15, 16, and 17) to Cycle 2, Day 17. In subsequent cycles, MLN8237 therapy will be initiated on Day 1 of the cycle and administered 1 hour before the start of the paclitaxel infusion. If DLTs are observed in 0 of 3 patients, the next cohort will open. If 1 drug-related DLT is observed in the first cycle, the cohort will be expanded. If DLTs are observed in 1 of 6 patients, the next cohort will open with MLN8237 administered at the next higher dose level, according to the Dose Escalation Schema (see Study Flow Diagram).
- If DLTs are observed in 2 of 6 patients, the MLN8237 dose will be reduced to the next lower dose level (see Table 6-1) and expanded to assure not more than 1 in 6 (or 0 in 5) DLTs. This will continue until the recommended MTD of MLN8237 plus weekly paclitaxel (maximum 80 mg/m^2 on Days 1, 8, and 15) is established. The MTD will require first cycle DLTs in not more than 1 in 6 (or 0 in 5) patients evaluable for DLT. Based on the nature and timing of toxicities, the schedule of MLN8237 may also be reduced for evaluation in new cohorts; for example, complicated neutropenia or mucositis attributed to MLN8237 and occurring around cycle Day 15 would lead to MLN8237 dose reduced to Days 1, 2, and 3 and 8, 9, and 10.
- For determination of the MTD in Phase 1, patients will be replaced in a cohort if withdrawn from treatment during Cycle 1 for reasons other than DLT, including the following situations: failure to complete 1) at least 80% of the planned doses of MLN8237; or 2) at least 2 of the planned 3 doses of paclitaxel; or 3) the required safety evaluations scheduled in Cycle 1 to determine DLT.

Table 6-1 MLN8237 Combined With Paclitaxel for Phase 1 Dose Escalation

Cohort	Target MLN8237 (PO) Dose and Schedule	Target Paclitaxel (IV) Dose and Schedule
**Starting Dose Level** 1	10 mg BID for 3 days on/4 days off repeated weekly ×3 weeks in 28-day cycle	80 mg/m^2 on Days 1, 8, 15
2	20 mg BID for 3 days on/4 days off repeated weekly ×3 weeks in 28-day cycle	80 mg/m^2 on Days 1, 8, 15
3	40 mg BID for 3 days on/4 days off repeated weekly ×3 weeks in 28-day cycle	80 mg/m^2 on Days 1, 8, 15
4	50 mg BID for 3 days on/4 days off repeated weekly ×3 weeks in 28-day cycle	80 mg/m^2 on Days 1, 8, 15
5+	Escalate by 25% to MTD	80 mg/m^2 on Days 1, 8, 15

Abbreviations: IV = intravenous; PO = orally, by mouth

The Phase 1 portion is designed with the intent of maintaining a standard starting dose and schedule for weekly paclitaxel in the combination regimen. If the combination of MLN8237 plus weekly paclitaxel 80 mg/m^2 is not tolerated, paclitaxel dose reductions (eg, to 70 or 60 mg/m^2) can be evaluated in new cohorts upon agreement by the sponsor and investigators, and review of data from initial cohorts and PK results. If paclitaxel at a reduced dose is found to be well tolerated by an individual patient when administered with MLN8237, the paclitaxel dose may be re-escalated to 80 mg/m^2 upon agreement by the sponsor and investigators and review of data from initial cohorts and PK results as described in Section 4.1. The doses and schedules employed in the de-escalation or re-escalation cohorts, and the design of other cohorts if required to determine the RP2D schedule for the MLN8237 plus paclitaxel combination, may be modified based on the PK and safety experience from initial cohorts. Based on the nature and timing of toxicities, the schedule of MLN8237 may also be reduced; for example, complicated neutropenia or mucositis attributed to MLN8237 and occurring on or around cycle Day 15 would lead to MLN8237 dosing reduced to Days 1, 2, and 3 and 8, 9, and 10. If the lower dose of pacitaxel is tolerated, the dose of MLN8237 may be re-escalated with that same lower dose of paclitaxel. Although the dose of paclitaxel used in combination with MLN8237 may be reduced in the Phase 1 portion of the study, the starting weekly, single-agent paclitaxel dose for the reference arm of the randomized Phase 2 portion will not be reduced, and it is planned to be a starting dose of 80 mg/m^2, allowing adjustments of dose or schedule based on individual patient tolerance.

Upon determination of the RP2D regimen to be used in the Phase 2 portion, the sponsor and Phase 1 investigators will review safety and tolerability of patients who received paclitaxel escalated from a reduced starting dose up to 80 mg/m^2; if the Phase 1 data indicate that such paclitaxel escalation was generally tolerable, this option will be continued in the Phase 2 portion for individual patients enrolled to the combination arm. After a paclitaxel dose escalation, management of toxicities by dose reduction, delays, or supportive care continue to follow protocol guidelines (Section 6.5).

The Phase 2 randomized study will begin upon agreement regarding the planned RP2D dose and schedule among the Phase 1 investigators, sponsor, and SMC. Determination of the RP2D will include analysis of tolerance by patients, treated with the combination, for 2 or more cycles without evidence of major cumulative toxicities such as progressive Grade 4 thrombocytopenia or complicated/prolonged Grade 4 neutropenia. In addition, determination of the RP2D will include analysis of patients who received the planned full doses, to be recommended for Phase 2, over 2 or more cycles, and without a requirement for myeloid growth factors during the first 2 cycles. At the RP2D, if a minimum of 3 patients have not completed 2 or more cycles due to reasons (eg, PD) other than safety, then they will be replaced so that a minimum of 3 patients have received and tolerated the RP2D for 2 or more cycles (without a requirement for myeloid growth factors during the first 2 cycles) in support of this dose and schedule to be used in Phase 2.

Patients who withdraw from the study for any reason may continue with alternative doses or schedules of taxane therapy at the discretion of the treating physician.

6.5 Dose-Modification Guidelines

Toxicity will be evaluated according to the NCI CTCAE, version 4.02, effective as of 01 October 2009. These criteria are provided in the Study Manual and are available online at http://ctep.cancer.gov/reporting/ctc.html.

6.5.1 Criteria for Beginning a Subsequent Treatment Cycle

In order for a new cycle of therapy to begin the following criteria must be met:

- ANC must be ≥ 1,500/mm^3
- Platelet count must be ≥ 75,000/mm^3
- All other toxicity considered by the investigator to be related to therapy with

MLN8237 (except alopecia) or paclitaxel must have resolved to ≤ Grade 1, or to a level considered acceptable by the physician, or to the patient's baseline values before a new cycle of therapy may begin.

- There has been a minimum rest period of 10 days or longer since the last dose of study drug.

6.5.2 Criteria for Dose Interruption During a Cycle

If a patient experiences any of the following toxicities during the dosing period, dosing will be discontinued for the remainder of that cycle, or until recovery sufficient for completion of the cycle or the start of a new treatment cycle.

- Grade 4 neutropenia (ANC ≤ 500 cells/mm^3)
- Grade 4 thrombocytopenia (platelet count < 25,000/mm^3)

Once the criteria for retreatment (Section 6.5) have been met, the patient may resume therapy. If the Day 8 or Day 15 doses of paclitaxel and MLN8237 (or paclitaxel alone if applicable) have been delayed through 2 additional days or less (eg, Monday delayed to Wednesday, but restart possible on Thursday), dosing should resume after the delay without omitting doses, and without dose reduction. The subsequent dose of the cycle should preserve the same minimal days between doses within the cycle originally planned; thus, if the planned Day 8 dose is delayed but restarted on Thursday, then the next planned "Day 15" dose would be administered on the following Thursday to preserve a 7-day period between the Day 8 and Day 15 doses.

If the Day 8 or Day 15 doses of paclitaxel and MLN8237 (or paclitaxel alone, if applicable) have been delayed through additional days due to lack of adequate recovery (eg: dosing originally planned for Monday delayed through Thursday, and restart only possible on Friday or later), both the paclitaxel dose and the 3 associated MLN8237 doses (if applicable) should be omitted within that cycle. If the doses started on Day 15 are omitted, the cycle should preserve a minimum 2-week treatment-free period between the last dose of protocol treatment and the start of the new cycle. When paclitaxel and MLN8237 (if applicable) are resumed, either on Day 15, or with the subsequent cycle, the doses of paclitaxel and/or MLN8237 should be modified, with incremental reductions if appropriate (see Section 6.5.3).

6.5.3 Dose Modifications

The dose modification rules are intended for use in a similar manner for patients who are assigned to single-agent paclitaxel in the Phase 2 portion, and for patients assigned to the combination in the Phase 1 or Phase 2 portions. If extenuating circumstances prevent modification of the dose or schedule as described, the patient may continue in the study upon review and agreement by the medical monitor.

To manage hematologic or non-hematologic toxicities that require dose reductions, the dose modifications planned for this protocol will include the following:

Paclitaxel: 10 mg/m^2 increment reductions from planned starting dose, administered on Cycle Days 1, 8, and 15. For example, the single-agent paclitaxel arm will evaluate dose levels of 80, 70, or 60 mg/m^2 with option for further dose reduction by 10 mg/m^2 increments in some situations described below.

MLN8237: 10-mg increment reduction from the planned starting dose (total reduction 20 mg/day when administered on BID schedule).

For patients assigned to the combination paclitaxel and MLN8237, the decision regarding which study drug requires dose reduction will be dependent upon the toxicity, its onset, and time course. For example, neuropathy has been related to paclitaxel but it has not been a frequent or dominant toxicity associated with MLN8237. Although somnolence can be observed from multiple causes in patients with advanced malignancy, it has been observed in some patients as a toxicity associated with high individual doses of MLN8237, typically within the first few days of drug administration. Thus, the dose of paclitaxel alone will be adjusted for dose-limiting nonhematologic toxicities such as neuropathy, and the dose of MLN8237 alone will be reduced for dose-limiting non-hematologic toxicities such as somnolence that is not due to other comorbidities.

Paclitaxel or MLN8237 (if applicable in patients receiving the combination) will be individually reduced for dose-limiting hematologic toxicities that are attributable to both agents, such as sustained Grade 4 neutropenia, thrombocytopenia or febrile neutropenia according to Table 6-2. As a general approach to manage these hematologic toxicities which are also attributable to the taxane, the paclitaxel should be first delayed and/or reduced prior to initiation of myeloid growth factors or prior to modification of MLN8237, if applicable. To manage dose-limiting neutropenia attributable to paclitaxel alone or to the combination with MLN8237, the general goals include a) preserving balance as possible

between paclitaxel dose delivery in the 2 arms of the Phase 2 portion of study, and b) to avoid reducing MLN8237 dosing below a range considered to be clinically relevant. Given these considerations, the first intervention will be dose reduction of paclitaxel by 10 mg/m^2 (eg, from a planned dose of 80 to 70 mg/m^2 in both arms). The next intervention would be to add myeloid growth factor without further paclitaxel dose reduction. Only after interventions that include myeloid growth factor for hematological toxicity would the paclitaxel dose be reduced a second time in the single-agent cohort, or to a level below 60 mg/m^2 in patients in the combination arm who may start at that dose level. Similarly, only after intervention with myeloid growth factor would the MLN8237 be reduced by 10 mg/dose in the combination arm.

Prophylactic use of myeloid growth factors are not allowed for patients newly enrolled who receive the first cycle, but may be administered for supportive care to manage neutropenia events if clinically indicated, according to ASCO Guidelines and/or institutional practices. Thus, myeloid growth factors are not mandated, but if used per investigator discretion, they should be administered according to local guidelines, the product label, and Table 6-2. During the first cycle of patients enrolled to the Phase 1 study, the use of myeloid growth factors should be avoided unless DLT criteria have already been met. Short acting myeloid growth factors are preferred, and they should be discontinued for an appropriate number of days prior to restart of protocol treatment.

Table 6-2 Dose Modification Rules – Hematological Toxicity Attributable to Taxane With or Without MLN8237

	Paclitaxel + MLN8237	Single-Agent Paclitaxel
1st occurrence	Reduce paclitaxel 1 level	Reduce paclitaxel 1 level
Repeat occurrence	Add myeloid growth factor without further dose modification, if appropriate	Add myeloid growth factor without further dose modification, if appropriate
Repeat occurrence within dosing period	Reduce MLN8237 1 level[a]	Reduce paclitaxel 1 additional level[a]
Repeat occurrence after dosing complete, in treatment-free period	Omit MLN8237 in last 3 days of schedule	Reduce paclitaxel 1 additional level
Repeat occurrence on paclitaxel 60 mg/m^2	Discontinue[b]	Discontinue[b]

If myeloid growth factors are administered to manage neutropenia prior to any previous dose reduction, the next cycles should be dose reduced prior to re-initiation of growth factors if clinically appropriate.

a Myeloid growth factors may be administered, maintaining the same dose level, prior to reduction of paclitaxel below 60 mg/m^2 in either arm or prior to reduction of MLN8237 in the combination arm.

b A patient may continue protocol with further dose reductions if deriving clinical benefit, upon review by the medical monitor.

If a combination regimen is investigated in the Phase 1 portion with a starting dose of paclitaxel below 80 mg/m^2 and the individual patient tolerates the starting doses without severe (CTC Grade 3 or higher) toxicity, the paclitaxel dose can be escalated after completion of Cycle 2 up to a maximum of 80 mg/m^2 and safety monitoring will continue as required by the Schedules of Events. Upon determination of the RP2D regimen to be used in the Phase 2 portion, the sponsor and Phase 1 investigators will review safety and tolerability of patients who received paclitaxel escalated from a reduced starting dose up to 80 mg/m^2; if the Phase 1 data indicate that such paclitaxel escalation was generally tolerable, this option will be continued in the Phase 2 portion for individual patients enrolled to the combination arm.

<u>Regimen Modifications for Hematologic Toxicities according to Cycle Day Onset Time:</u>

1. **During dosing period (eg, Days 1 to 15 after single-agent paclitaxel, or Days 1 to 17 with the combination of paclitaxel plus MLN8237):**

   - FIRST OCCURRENCE: Patients on single-agent paclitaxel or combination reduce paclitaxel by 10 mg (eg,: 80 to 70 mg/m^2) in future cycles
   - REPEAT OCCURRENCE: Myeloid growth factors may be administered, maintaining the same doses of study drug(s), per investigator discretion prior to reduction of paclitaxel below 60 mg/m^2 in either arm or prior to reduction of MLN8237 in the combination arm
   - REPEAT OCCURRENCE: Patients on single-agent paclitaxel or combination reduce paclitaxel by another 10 mg (eg, 70 to 60 mg/m^2) in future cycles

2. **After dosing period (eg, in the treatment-free period) upon recovery):**

   - FIRST OCCURRENCE: Patients on single-agent paclitaxel or combination reduce paclitaxel by 10 mg (eg, 80 to 70 mg/m^2) in future cycles
   - REPEAT OCCURRENCE: Myeloid growth factors may be administered, maintaining the same doses of study drug(s), per investigator discretion prior to reduction of paclitaxel below 60 mg/m^2 in either arm or prior to reduction of MLN8237 in the combination arm
   - REPEAT OCCURRENCE: Patients on single-agent paclitaxel reduce paclitaxel by another 10 mg (eg, 70 to 60 mg/m^2) in future cycles
   - REPEAT OCCURRENCE: Patients on combination omit the last 3 days (eg, Days 15, 16, 17) of MLN8237 in future cycles but maintain the same MLN8237 doses

administered on earlier cycle days

Dose Modifications for Non-hematologic Toxicities:

- Dose modifications will be as described above for hematologic toxicities; however, only the drug thought to be responsible for the toxicity should be reduced. If both MLN8237 and paclitaxel may have contributed, or the investigator is uncertain which drug is responsible, then both MLN8237 and paclitaxel should be modified using incremental reductions described above.
- In the Phase 2 portion, if a patient requires dose reduction due to a study drug-related toxicity, the drug dose may not be re-escalated. Similarly, if a patient requires elimination of MLN8237 on the last 3 days (eg, Days 15, 16 and 17) due to DLT, as described above, MLN8237 will only be dosed on the earlier cycle days as planned for all subsequent cycles.
- REPEAT OCCURRENCES: Patients who continue to experience dose limiting toxicity (hematologic or non-hematologic) on single-agent paclitaxel or the combination regimen with paclitaxel dose of 60 mg/m^2, given administration of appropriate supportive care, should discontinue protocol treatment. However, if the patient has clinical benefit and would benefit from continued protocol treatment, the patient may continue protocol treatment with further dose reductions, upon review by the medical monitor.

6.6 Excluded Concomitant Medications and Procedures

The following medications and procedures are prohibited during the study:

- Any antineoplastic therapy other than study drug (MLN8237 or paclitaxel)
- Any investigational therapy other than MLN8237
- Alternative therapy, including palliative radiotherapy, for treatment of the patient's malignancy (platinum-resistant epithelial ovarian, fallopian tube, or primary peritoneal carcinoma)
- Tamoxifen, thalidomide, or any agent used as maintenance or consolidation therapy for OC

- If MLN8237 is administered, requirement for constant administration of any proton pump inhibitor. Patients may be administered alternative agents to manage gastric acidity or reflux (eg, H2 receptor antagonists, antacids) with exceptions described below.
- Histamine-2 receptor antagonists are not permitted from the day prior (Day -1) through the last day of MLN8237 dosing (Day 17) in the treatment cycle, except as required for premedication for paclitaxel (see Section 6.2.2)
- Antacid preparations are not permitted for 2 hours before or 2 hours after administration of the MLN8237 dose.
- Hormonal therapy except hormone replacement therapy or birth control pills
- Clinically significant enzyme inducers including the enzyme-inducing antiepileptic drugs phenytoin, carbamazepine, or phenobarbital, or rifampin, rifabutin, rifapentine, or St. John's wort are not permitted within 14 days prior to the first dose of MLN8237 and during the study.

6.7 Permitted Concomitant Medications and Procedures

Myeloid growth factors may be used in accordance with ASCO guidelines.[(43)] As described in the ASCO guidelines, myeloid growth factors should not be administered as a general prophylactic agent to manage uncomplicated, transient neutropenia. Antiemetic agents may be administered at the discretion of the investigator, but prophylactic antiemetic agents should not be administered in the first cycle of treatment if nausea or vomiting is not observed. All other manifestations of the patient's malignancy should be treated at the discretion of the investigator.

Antacids are permitted; however, they should be administered more than 2 hours before or 2 hours after administration of MLN8237 (see also Section 6.6).

Low molecular weight heparin, administered as preventive treatment, is allowed if the patient has tolerated treatment with a stable dose and schedule without bleeding complications for more than 1 month.

Medications with potential CNS effects are not prohibited in this study, but it is recommended that their use be minimized to avoid confusion in the interpretation of CNS effects should they occur during the course of treatment with MLN8237. Because of MLN8237's structural and pharmacological similarity to the benzodiazepines, concomitant

therapy with benzodiazepines is discouraged but not prohibited. Medications such as typical or atypical antipsychotic and antidepressant agents are permitted.

Premedication with corticosteroids, diphenhydramine, or histamine-2 receptor antagonists is permitted before each treatment with paclitaxel.

All other medical conditions should be treated at the discretion of the investigator in accordance with local community standards of medical care.

6.8 Precautions and Restrictions

Food and drinks other than water and prescribed medications are not permitted for 2 hours preceding and 1 hour following each dose of study drug. Administration options and guidelines may be modified by the sponsor and provided in the Study Manual.

Patients should not drive, operate dangerous tools or machinery, or engage in any other potentially hazardous activity that requires full alertness and coordination if they experience sedation while enrolled in this study.

Patients are to be instructed to limit the use of alcohol while enrolled in this study. Patients should consume no more than 1 standard unit of alcohol per day during the study and for 30 days from the last dose of study treatment. A standard unit of alcohol is defined as a 12 oz beer (350 mL), 1.5 oz (45 mL) of 80-proof alcohol, or one 6-oz (175 mL) glass of wine.

6.9 Management of Clinical Events

6.9.1 Nausea and Vomiting

Prophylactic antiemetic therapy will not be used in this study unless it becomes clear that MLN8237 plus weekly paclitaxel or weekly paclitaxel alone causes acute nausea and vomiting. If prophylactic antiemetic therapy is needed, 5-HT_3 receptor antagonists (without corticosteroids) should be tried first; however, prophylactic antiemetic agents should not be administered in the first cycle of treatment if nausea or vomiting is not observed. Because of the potential for benzodiazepines to cause sedation, the use of benzodiazepines for antiemetic prophylaxis should be reserved for patients who cannot be satisfactorily managed otherwise.

Although this study will not initially employ prophylactic antiemetics, there is no prohibition against antiemetic use in the management of a patient who develops nausea or vomiting, or both.

6.9.2 Diarrhea

Antidiarrheal medications will not be used prophylactically; however, patients will be instructed to take loperamide, 4 mg, at the occurrence of the first loose stool and then 2 mg every 2 hours until they are diarrhea-free for at least 12 hours. During the night, patients may take 4 mg of loperamide every 4 hours. Fluid intake should be maintained to avoid dehydration.

6.9.3 Central Nervous System Effects

If a patient experiences excessive sedation believed to be related to MLN8237, treatment with MLN8237 should be interrupted. Patients whose sedation is not considered immediately life-threatening should be carefully monitored and given appropriate supportive care.

[redacted]

[redacted]

6.9.4 Hypersensitivity Reactions Associated with Paclitaxel

Minor symptoms such as flushing, skin reactions, dyspnea, hypotension, or tachycardia do not require interruption of paclitaxel therapy. Severe reactions, however, such as

hypotension requiring treatment, dyspnea requiring bronchodilators, angioedema, or generalized urticaria require immediate discontinuation of paclitaxel and aggressive symptomatic therapy. Patients who have developed severe hypersensitivity reactions should not be rechallenged with paclitaxel. Please refer to the paclitaxel product label for further information.

6.10 Blinding and Unblinding

This is an open-label study.

6.11 Description of Investigational Agents

MLN8237 drug product is supplied as the ECT dosage form in 10 mg or 50 mg strengths, with dose strength expressed as the milligrams of active drug (free acid); other strengths may be supplied based on the observed MTD. The key formulation excipients of the [redacted]

Paclitaxel will be supplied as a solution for injection in a 100 mg/16.7 mL vial labeled as investigational material.

6.12 Preparation, Reconstitution, and Dispensation

MLN8237 ECT are packaged in a 60-cc high-density polyethylene (HDPE) bottle with a rayon coil, induction seal, desiccant packs, and a polypropylene child-resistant cap.

Please refer to the paclitaxel product label for instructions and precautions regarding preparation and handling.

MLN8237 and paclitaxel are anticancer drugs, and as with other potentially toxic compounds, caution should be exercised when handling MLN8237 and paclitaxel. It is recommended that gloves and protective clothing be worn during preparation.

6.13 Packaging and Labeling

The packaged and labeled study drug, MLN8237 ECT, will be provided by Millennium and will be handled at the investigative site as open-label material. The labels on the study drug will fulfill all requirements specified by governing regulations. MLN8237 will be supplied as ECT in 10 mg or 50 mg strengths. The 60-cc HDPE bottles will have a child-resistant

cap and be labeled for take-home use. Patients will receive instructions for home use of MLN8237, including the requirement that MLN8237 be administered as intact tablets.

Paclitaxel will be labeled as investigational material and provided to the investigative site by Millennium; packaging labels will fulfill all requirements specified by governing regulations. Paclitaxel will be supplied as a solution for injection in a 100 mg/16.7 mL vial (6 mg/mL).

As required by local regulations, any modifications to the plan for drug supply or storage will be communicated to the investigator and detailed in the Study Manual.

6.14 Storage, Handling, and Accountability

Tablets should remain in the bottle provided until use. The container should be stored at the investigative site at controlled room temperature (20°C to 25°C; 68°F to 77°F; excursions permitted from 15°C to 30°C; 59°F to 86°F) and used before the retest expiry date provided by Millennium. The stability of the drug product will be monitored for the duration of the clinical studies. Tablets are not intended to be broken or manipulated in any way. Containers should be kept closed during storage.

Because MLN8237 is an investigational agent, it should be handled with due care. In case of contact with broken tablets, raising dust should be avoided during the cleanup operation.

The product may be harmful by inhalation, ingestion, or skin absorption. Gloves and protective clothing should be worn during the cleanup operation. The area should be ventilated and the spill site washed after material pick-up is complete. The spilled material should be disposed of as hazardous medical waste in compliance with federal, state, and local regulations.

In case of contact with the powder (eg, from a broken tablet), skin should be washed immediately with soap and copious amounts of water for at least 15 minutes.

In case of contact with the eyes, copious amounts of water should be used to flush the eyes for at least 15 minutes. Medical personnel should be notified.

Patients are to be instructed on proper storage, accountability, and administration of MLN8237, including that MLN8237 is to be taken as intact tablets.

Vials of paclitaxel should be stored at 20°C to 25°C (68°F to 77°F), protected from light. Please refer to the paclitaxel product label for further information regarding the proper storage and handling of paclitaxel.

Procedures for drug accountability at the investigational site are described in Section 11.11.

6.15 Other Protocol-Specified Materials

This section is not applicable.

7. STUDY CONDUCT

7.1 Study Personnel and Organizations

All contact information for this study is presented in the Study Manual and/or supporting documentation. Please refer to the Study Manual for contact information for the medical monitor, the central and any additional clinical laboratories, and the [redacted] physician. A full list of investigators is available in the sponsor's investigator database.

7.2 Arrangements for Recruitment of Patients

Recruitment and enrollment strategies for this study may include recruitment from the investigators' local practices or referrals from other physicians. If advertisements become part of the recruitment strategy, they will be reviewed by the sponsor before approval by the institutional review board (IRB)/independent ethics committee (IEC). Any other arrangements will be described in the Study Manual.

7.3 Treatment Group Assignments

Patient accrual and treatment during the Phase 1 portion of the study is discussed in Section 6.4. During the Phase 2 portion of the study, a centralized randomization procedure will be used to assign patients in a 1:1 ratio to treatment with either MLN8237 plus weekly paclitaxel or weekly paclitaxel alone.

7.4 Study Procedures

The timing of the study procedures outlined in the following subsections is provided in the Schedules of Events. When applicable, specific visit windows for assessments are provided in the footnotes to the study schedules. The timing of assessments to be performed, but not the number (unless fewer), will be updated in the Study Manual.

Each treatment cycle is 28 days in length. Tests and procedures should be done on schedule, but visit windows of ± 2 days are allowed (except as otherwise specified) occasionally for holidays, vacations, and other administrative reasons. **If extenuating circumstances prevent a patient from beginning treatment or completing a scheduled assessment within this time frame, the patient may continue in the study only with written permission of the medical monitor.**

Cycle 1, Day 1 (baseline) procedures/tests are to be performed within the 4 days prior to the Cycle 1, Day 1 dose of study drug. If any screening values or results also required at Cycle 1, Day 1 were obtained and acceptable within the 4 days prior to the Cycle 1, Day 1 dose of study drug, those procedures/tests need not be repeated at Cycle 1, Day 1.

Refer to the Schedules of Events for the timing of all assessments. Additional details are provided as necessary in the sections that follow.

7.4.1 Informed Consent

Each patient or each patient's legal representative must provide written informed consent before any study-related procedures are conducted, unless those procedures are performed as part of the patient's standard care.

7.4.2 Inclusion and Exclusion Criteria

The inclusion and exclusion criteria will be assessed during screening (within 28 days before the first dose of any study drug on Cycle 1, Day 1). A patient is considered enrolled when administered the first dose of either paclitaxel or MLN8237 in the Phase 1 portion or randomized to treatment in the Phase 2 portion.

The RP2D will be determined by a minimum of 6 patients evaluable for safety, at least 4 with recurrent OC and at least 4 who tolerate Cycle 1 without a requirement for myeloid growth factor support. Six additional patients will be enrolled in the expansion cohort to refine understanding of safety and PK.

Procedures for completion of enrollment information, and requirements to communicate with the sponsor about enrollment, are described in the Study Manual.

7.4.3 Patient Demographics

Patient demographics, including patient birth date, race, and ethnicity, will be documented during screening (within 28 days before the first dose of any study drug).

7.4.4 Medical History and Physical Examination

During the Screening period (within 28 days before the first dose of any study drug), a complete medical history will be obtained and a complete physical examination, including neurological examination, performed. The medical history will emphasize the history of the patient's malignancy and FIGO (International Federation of Gynecology and Obstetrics) stage with a description of all relevant prior therapies administered for OC (Phases 1 and 2) or breast cancer (Phase 1 only) and dates of recurrence of OC (Phase 2 only) up to the time of the first dose of study drug (paclitaxel or MLN8237). If available, relevant markers, including BRCA (breast cancer gene), ER and PR (estrogen and progesterone receptors), and HER2 (human epidermal growth factor receptor 2) status, will be recorded. Additionally, concomitant medications will be listed and will include all medications being taken at the time of screening and within 28 days of the first dose. The screening physical examination will include the patient's height (Section 7.4.6) and weight (Section 7.4.7) and will also include an assessment of the patient's ECOG PS (see Section 7.4.8).

The symptom-directed physical examination will be performed at Cycle 1, Day 1 (baseline), and within 3 days before the beginning (Day 1) and on Days 8, 15, and 21 (± 2 days) of each treatment cycle. In patients who tolerate study treatment through multiple cycles, the Day 21 symptom-directed physical examination can become optional, at the discretion of the investigator, if in the prior 2 cycles the patient tolerated study treatment (ie, without the requirement for dose reduction or without ≥ Grade 3 treatment-related toxicity evaluated according to NCI CTCAE, version 4.02).

The Cycle 1, Day 1 (baseline) symptom-directed physical examination is not required if the screening medical history was obtained and physical examination was conducted within the 4 days prior to administration of the Cycle 1, Day 1 dose of study drug.

During the symptom-directed physical examination, neurotoxicity will be monitored through AE reporting and concomitant medication usage.

The symptom-directed physical examination will be repeated at the EOT visit and at the EOS visit (see Schedules of Events). The symptom-directed physical examination will be directed toward the identification of new symptoms and signs, and changes in body weight (Section 7.4.7), ECOG PS (Section 7.4.8), or concomitant medications (Section 7.4.17).

7.4.5 Vital Signs

Vital signs (systolic and diastolic blood pressures, heart rate, and oral temperature) measurements will be obtained during screening; at Cycle 1, Day 1 (baseline); and within 3 days before the beginning (Day 1) and on Days 8, 15, and 21 (± 2 days) of each treatment cycle. If the screening assessment was done within 4 days prior to Cycle 1, Day 1, an assessment at Cycle 1, Day 1 is not necessary.

Blood pressure should be determined with the patient in a seated position after the patient has been sitting quietly for 5 minutes.

Vital signs measurements also will be obtained at the EOT and EOS visits.

7.4.6 Patient Height

Height will be measured only during screening (within 28 days before the first dose of any study drug).

7.4.7 Patient Weight

During Phase 1, weight will be measured during screening; at Cycle 1, Day 1 (baseline); and within 3 days before the beginning (Day 1) of each treatment cycle.

During Phase 2, weight will be measured during screening; at Cycle 1, Day 1 (baseline); and within 3 days before the beginning (Day 1) of each treatment cycle. Paclitaxel dosing may be adjusted for a significant change (eg, > 10%) in body weight with agreement by the medical monitor.

If the screening assessment was done within 4 days prior to Cycle 1, Day 1, an assessment at Cycle 1, Day 1 is not necessary.

Weight also will be measured at the EOT visit and the EOS visit.

7.4.8 Eastern Cooperative Oncology Group Performance Status

ECOG PS (Section 15.1) will be assessed during screening; at Cycle 1, Day 1; within 3 days before the beginning (Day 1) of each treatment cycle; at the EOT visit; and at the EOS visit. If the screening assessment was done within 4 days prior to Cycle 1, Day 1, an assessment at Cycle 1, Day 1 is not necessary.

7.4.9 Electrocardiogram

A 12-lead ECG will be obtained at screening and at Cycle 1, Day 1 (baseline). If the screening ECG was obtained within the 4 days prior to the Cycle 1, Day 1 dose of study drug, a repeat ECG at Cycle 1, Day 1 is not necessary. Additional ECGs may be obtained if clinically indicated. ECG assessments are to be performed with patient supine and rested for 5 minutes and before any closely timed blood collection.

7.4.10 Clinical Laboratory Evaluations

Blood samples for analysis of the following hematology and clinical chemistry parameters, and urine for urinalysis will be obtained as specified in the Schedules of Events. Clinical laboratory evaluations will be performed by local laboratories for the Phase 1 portion and by a central laboratory for the Phase 2 portion of the study. Management of some samples may be sent to the central laboratory for both Phases 1 and 2. Handling and shipment of clinical laboratory samples will be outlined in the Study Manual. Clinical laboratory evaluations will be performed as outlined below.

If screening values were obtained and acceptable within the 4 days prior to the Cycle 1, Day 1 dose of study drug, repeat clinical laboratory testing (hematology, clinical chemistry, or urinalysis) at Cycle 1, Day 1 is not necessary.

A blood sample for hematology (complete blood count [CBC] with differential WBC count) will be obtained during screening; within 3 days before the beginning (Day 1) of each treatment cycle; and on Days 8, 15, and 21 (± 2 days). In patients who tolerate study treatment through multiple cycles, the Day 21 clinical laboratory assessments can become optional, at the discretion of the investigator, if in the prior 2 cycles the patient tolerated study treatment (ie, without the requirement for dose reduction or without ≥ Grade 3 treatment-related toxicity evaluated according to NCI CTCAE, version 4.02). As noted above, central laboratory evaluations are required for Phase 2; however, if needed for immediate patient care or treatment decisions, blood samples may be obtained and sent to

local laboratories (in addition to the required central laboratory evaluations), with results entered into the eCRF. If a patient has an ANC less than 500/mm^3 or a platelet count less than 25,000/mm^3, or both, the CBC with differential should be repeated at least every 2 to 3 days until the ANC or platelet count (or both, if both were decreased) have exceeded these values on at least 2 occasions.

A blood sample for CBC with differential also will be obtained at the EOT visit.

Hematology

- Hemoglobin
- Hematocrit
- Platelet count
- White blood cell count with differential (neutrophils, lymphocytes, monocytes, basophils, and eosinophils) and absolute neutrophil count (ANC)

A blood sample for clinical chemistry panel will be obtained during screening; within 3 days before the beginning (Day 1) of each treatment cycle; and at the EOT visit.

Serum Chemistry

- Sodium
- Potassium
- Blood urea nitrogen (BUN)
- Creatinine
- Alkaline phosphatase
- AST (SGOT)
- ALT (SGPT)
- Total bilirubin
- Carbon dioxide (CO_2)
- Albumin
- Calcium
- Chloride
- Phosphate
- Magnesium
- Glucose

Thyroid stimulating hormone (TSH) level will be measured at Cycle 1, Day 1 (baseline) and at the EOT visit; this test should be repeated during the treatment period as clinically indicated.

A urine sample for urinalysis will be obtained during screening, at Cycle 1, Day 1 (baseline), and at the EOT visit.

Urinalysis

- Protein
- Blood

If urine protein by dipstick changes to 3+ (or 2+ that is reconfirmed at least 1 day later), then a 24-hour urine collection should be done for protein and creatinine clearance. The

screening or Cycle 1, Day 1 urinalysis should include microscopic examination of the sediment.

7.4.11 Pregnancy Test

A serum beta-human chorionic gonadotropin (β-hCG) pregnancy test will be performed only for patients of childbearing potential during screening and again at Cycle 1, Day 1 (baseline) if the screening test was performed more than 4 days before the first dose of MLN8237 or paclitaxel. The results must be negative within 4 days before the first dose of MLN8237 or paclitaxel is administered (ie, within the 4 days prior to Cycle 1, Day 1), or as otherwise required by local regulations.

Additional pregnancy testing may be performed during the study at the discretion of the investigator, as per request of IEC/IRB, or if required by local regulations.

If a patient becomes pregnant or suspects pregnancy while participating in this study, the investigator must be informed immediately (see Section 10.4).

7.4.12 Disease Assessment by CA-125

For patients with ovarian, fallopian tube, or peritoneal cancer, CA-125 levels will be obtained according to standard of care within 9 days of screening and sufficient time after prior therapy; within 4 days prior to the dose of study drug at Cycle 1, Day 1; at the end of every treatment cycle (during the 1 week prior to the start of a new cycle); at the EOT visit; and at the EOS visit (see Section 15.4). The investigator's determination of response by CA-125 is required at the end of every 2 cycles, designed to coincide with imaging studies for evaluation of response by RECIST criteria, if applicable. Two CA-125 levels must be obtained prior to dosing on Cycle 1, Day 1. CA-125 should be evaluated to determine if PD is substantiated prior to the start of each new cycle. Assessment of response or progression by CA-125 criteria requires an elevated baseline (Cycle 1, Day 1) value over 70 units/mL. Patients who discontinue study drug treatment before the occurrence of PD will be assessed at the EOT visit and at follow-up visits once every 8 weeks until the occurrence of 110 PFS events (PD or death, whichever occurs first) in Phase 2. It is noted that confirmation of response by CA-125 levels must be obtained at least 28 days after the previous sample (see the following).

A CA-125 response is defined as either of the following:[44]

- A 50% decrease from 2 initially elevated samples; the sample demonstrating the 50% decrease must be confirmed by a fourth sample (ie, a total of 4 samples is required)
- A serial decrease of > 75% over 3 samples (ie, a total of 3 samples is required)

In both the 50% and 75% response definitions, the final sample must be analyzed at least 28 days after the previous sample.

CA-125 progression based on CA-125 is defined as follows:[40, 41]

- For patients who enter the study with measurable target lesions and CA-125 levels ≤ 70 units/mL, or whose CA-125 levels are elevated (> 70 units/mL) pretreatment and normalize during the study, a CA-125 level of ≥ 2 times the ULN on 2 occasions
- For patients whose CA-125 levels are elevated (> 70 units/mL) pretreatment and do not normalize, a CA-125 level of ≥ 2 times the nadir value of CA-125 on 2 occasions

It is not known if MLN8237 and weekly paclitaxel treatment can be associated with rising CA-125 levels, even in some patients who may subsequently experience more definitive evidence of response or disease control. If an initial rising CA-125 is the only evidence of PD observed in a patient enrolled in either treatment arm, the patient may continue additional cycles of the assigned treatment in this study, if tolerated, upon request by the investigator and review of safety data by the medical monitor, coupled with serial monitoring for disease control.

7.4.13 Extent of Disease Evaluation by RECIST, Version 1.1

Extent of disease by diagnostic category will be evaluated according to RECIST, version 1.1 (see Section 15.3). Appropriate radiographic scans, including the chest, are to be performed during screening. If the patient has had appropriate scans performed within 28 days before the Cycle 1, Day 1 dose of study drug, those scans may be used for tumor lesion measurement during screening.

Repeat CT (with IV contrast) or MRI scans as appropriate, or both, are to be performed at the completion of Cycle 2 and every 2 cycles (approximately every 8 weeks, between

Days 21 and 28 of every even cycle) thereafter for up to 24 months or until PD is documented. The same imaging modality should be used throughout the study for each site of disease. For responders, radiographic images will be read locally but will be collected and provided to the sponsor for subsequent review. Scans are required at the EOT visit only if PD has not been documented previously and it has been 8 weeks or more since the previous evaluation.

Other procedures, such as physical examinations and other scintigraphic examinations (eg, bone scans for patients with known or suspected bone metastases), also should be taken into consideration when evaluating the extent of malignant disease.

Patients who discontinue study drug treatment before the occurrence of PD or death will be assessed at the EOT visit and followed off treatment during the Follow-Up period. During the Follow-Up period, patients will be followed off treatment once every 8 weeks until the occurrence of 110 progression-free survival (PFS) events (PD or death, whichever occurs first) are documented for this study. The EOS visit will be completed at the time the patient completes or withdraws from the Follow-Up period.

7.4.14 Response Markers in Banked Tumor Specimens

[redacted] Banked tumor tissue (submitted paraffin-embedded block is preferable, otherwise slides), if available, will be obtained as part of the patient's standard care before Cycle 1, Day 1 and, if available, earlier periods dating from the patient's original diagnosis. [redacted] Response markers will include analyses relevant to tumor type. The banked tumor tissue is to be collected only from enrolled patients, but may be collected and sent to the sponsor after initiation of protocol treatment. Details regarding the preparation, handling, and shipping of samples will be provided in the Study Manual.

7.4.15 Blood Sample for Genotyping

One blood sample will be obtained at Cycle 1, Day 1 before administration of the first dose of study drug (may be obtained within the 4 days prior to Cycle 1, Day 1), [redacted]

[redacted]

7.4.16 Blood Sample for Serum Markers of Response

During Phase 2, blood samples will be obtained at Cycle 1, Day 1 before the first dose of MLN8237 and at the scheduled visit on Day 15 of Cycle 1. In subsequent cycles, a blood sample will be obtained on Day 8 of **Cycle 2 only**. [redacted] Details regarding the preparation, handling, and shipping of samples are provided in the Study Manual.

7.4.17 Concomitant Medications and Procedures

Concomitant medications and supportive therapies will be recorded from 28 days prior to the first dose of study drug through the 30 days after the last dose of study drug or until the start of subsequent antineoplastic therapy, whichever occurs first. Medications with potential CNS effects are not prohibited in this study, but it is recommended that their use be minimized to avoid confusion in the interpretation of CNS effects should they occur during the course of treatment with MLN8237. Because of MLN8237's structural and pharmacological similarity to the benzodiazepines, concomitant therapy with benzodiazepines is discouraged but not prohibited. See Section 6.6 and Section 6.7 for a list of excluded and permitted concomitant medications and procedures, respectively.

7.4.18 Adverse Events

Monitoring of AEs, both nonserious and serious, will be conducted throughout the study as specified in the Schedules of Events. Refer to Section 10 for details regarding definitions, documentation, and reporting of pretreatment events, AEs, and SAEs.

7.4.19 Pharmacokinetic Measurements

Phase 1 Portion of the Study

Paclitaxel Pharmacokinetic Measurements

Paclitaxel PK measurements will be performed on Day 1 of Cycle 1 (weekly paclitaxel dosed during concomitant administration of MLN8237) and on Day 1 of Cycle 2 (weekly paclitaxel administered alone). Blood samples for the measurement of plasma concentrations of paclitaxel will be collected at the following time points on Day 1 of both Cycle 1 and Cycle 2 (see also Section 15.6):

- Within 1 hour before the start of the paclitaxel infusion
- At the end of the paclitaxel infusion (immediately before switching off the infusion pump)
- 5 (± 1) minutes, 15 (± 3) minutes, 0.5 hour (± 5 minutes), 1 hour (± 10 minutes), 2 hours (± 20 minutes), 3 hours (± 20 minutes), 7 hours (± 30 minutes), 10 hours (± 30 minutes), 23 (± 2) hours, and 47 (± 4) hours after stopping the paclitaxel infusion (ie, from the time the infusion pump is switched off). The 23- and 47-hour blood samples will be collected prior to MLN8237 dosing on those days.

MLN8237 Pharmacokinetic Measurements

Blood samples to measure plasma concentrations of MLN8237 will be collected at the following time points on Day 1 and Day 3 of Cycle 1 (see also Section 15.6):

- immediately before the morning dose of MLN8237
- 1 hour (± 10 minutes), 2 hours (± 20 minutes), 3 hours (± 20 minutes), 4 hours (± 20 minutes), 5 hours (± 20 minutes), 9 hours (± 30 minutes), and 12 (± 1) hours after the MLN8237 morning dose on Days 1 and 3.

Phase 2 Portion of the Study

For patients who are assigned to treatment with the combination of MLN8237 plus weekly paclitaxel, MLN8237 PK measurements will be performed during Cycle 1 at the following time points: on Day 1 before MLN8237 dosing, before initiation of the paclitaxel infusion, and following completion of the paclitaxel infusion; on Day 8 before MLN8237 dosing and at the end of the paclitaxel infusion; and on Day 15 at the time of the clinic visit. The date

and time of PK blood sampling and the date and time of administration of the dose of MLN8237 immediately preceding each PK sample should be recorded on the eCRF based on the entries in the patient's dosing diary or site source documentation.

In both Phase 1 and Phase 2, a blood sample to measure MLN8237 or paclitaxel concentrations, or both, should be obtained if clinically feasible at a time coincident with a serious and/or unusual AE that is judged by the investigator to be treatment-related irrespective of the cycle/day of the AE.

The primary intent of PK sample collections in this study is for the measurement of MLN8237 plasma concentrations; however, depending on technical feasibility, these samples may be utilized additionally for exploratory analysis of metabolite profiles.

The option to modify the schedule of PK sampling will be based on the PK and safety experience from initial cohorts in this study or other MLN8237 clinical studies. On days where PK sampling is required, PK samples should be drawn prior to dosing with MLN8237 if applicable.

7.4.20 Pharmacodynamic Measurements

Not applicable

7.4.21 Administration of Paclitaxel

Paclitaxel (starting dose 80 mg/m^2) will be administered as an IV infusion on Days 1, 8, and 15 of each 28-day treatment cycle. During the Phase 1 portion of the study on the days of PK assessment (ie, Day 1 of Cycles 1 and 2), paclitaxel should be infused over a period of 60 (± 10) minutes. The starting dose of paclitaxel for each patient will be calculated based on the patient's actual BSA without an arbitrary maximum BSA value and the dose not readjusted unless there is a significant (> 10%) change in weight (see below). Maximum BSA values (eg, 2 m^2) should not be used. Premedication to prevent paclitaxel-associated (hypersensitivity or other) reactions is required and should be administered as per standard institutional practice and according to the following guidelines. Premedications used in this setting can include 1 or more of the following agents: corticosteroid (dexamethasone with maximum dose 20 mg); histamine-2 antagonists (such as cimetidine or ranitidine); 5-HT_3 serotonin receptor antagonist antiemetic at its labeled dose (but prophylactic antiemetic agents should not be administered in the first cycle of treatment if nausea or vomiting is not observed); or diphenhydramine (maximum dose 25 mg). An example is a single dose of

dexamethasone (maximum 20 mg) administered PO or IV within 60 minutes before each paclitaxel infusion. Benzodiazepines are to be avoided. On days when both MLN8237 and paclitaxel are administered on the same morning, MLN8237 should be administered 1 hour before the start of the paclitaxel infusion. Modifications to paclitaxel administration or to the premedications are allowed upon agreement by the medical monitor and will be documented in the eCRF. The paclitaxel dose can be recalculated based on significant weight change (eg, > 10%) during the conduct of the study.

7.4.22 Administration of MLN8237

MLN8237 will be given PO BID, each dose with 1 cup (approximately 240 mL) of water, for 3 consecutive days followed by a 4-day recovery period, repeated weekly for 3 weeks in a 28-day treatment cycle (with the exception of Cycle 2 during Phase 1 only; see below). Patients will be instructed to take nothing by mouth except water and prescribed medications for 2 hours before and 1 hour after each dose. Patients should be instructed to take their study medication at approximately the same time each day and to not take more than the prescribed dose at any time. The 2 daily doses must be taken at least 6 hours apart. Patients should swallow the study medication whole and not chew it before swallowing.

In the event that a patient fails to take the MLN8237 dose within the time frame specified (± 3 hours and at least 6 hours between doses), that dose should be omitted. Patients should record any missed doses in their dosing diary (see Study Manual) and resume dosing at the next scheduled time with the prescribed dosage. In the event of emesis occurring after study medication ingestion, patients should simply adhere to the dosing schedule and resume dosing at the next scheduled time with the prescribed dosage. Patients should record the time of the emesis in their dosing diary (see Study Manual). Under no circumstance should a patient repeat a dose or double-up doses.

During Cycle 1 of the Phase 1 portion, the first dose of MLN8237 will be administered on Day 1 (ie, Cycle 1, Day 1), 1 hour before of the start of the paclitaxel infusion. During Cycle 2 of the Phase 1 portion, the first dose of MLN8237 will be administered on Day 8 (ie, Cycle 2, Day 8) in the study clinic to enable PK testing of the first dose of paclitaxel in the absence of concomitantly administered MLN8237; dosing of MLN8237 will continue up to Cycle 2, Day 17 (12 total doses of MLN8237 in Cycle 2 only). Based on the nature and timing of toxicities, the schedule of MLN8237 may be reduced; for example, complicated neutropenia or mucositis attributed to MLN8237 and occurring on or around cycle Day 15 would lead to MLN8237 dosing reduced to Days 1, 2, and 3 and 8, 9, and 10. During

subsequent cycles in Phase 1 and all cycles in Phase 2, the first dose of MLN8237 will be administered beginning on Day 1 of each treatment cycle and continue as tolerated. MLN8237 will be dispensed to the patient on Day 1.

During Phase 1 and for the combination regimen in Phase 2, on days when both paclitaxel and MLN8237 are administered, MLN8237 should be administered 1 hour before the start of the paclitaxel infusion.

Patients may continue to receive repeated cycles of MLN8237 treatment for up to 24 months, until there is evidence of PD or unacceptable treatment-related toxicity, or until another antineoplastic therapy is started. Upon request by the investigator, treatment with MLN8237 may be continued beyond 24 months if after discussion between the investigator and the sponsor it is determined that a patient would derive benefit from continued therapy. Patients treated with the combination regimen MLN8237 plus weekly paclitaxel who achieve disease control and who develop intolerance that is reasonably attributable to paclitaxel (eg, acute hypersensitivity immediately after paclitaxel infusion despite premedication) may continue single-agent MLN8237 on study upon request by the investigator and agreement by the sponsor. Similarly, patients treated with the combination regimen MLN8237 plus weekly paclitaxel who achieve disease control and who develop intolerance that is reasonably attributable to MLN8237 may continue single-agent paclitaxel on study upon request by the investigator and agreement by the sponsor.

7.4.23 Patient Diary

The study center staff will check the patient diary versus the patient's supply of MLN8237 ECT on Day 8 (for Days 1 to 3), Day 15 (for Days 8 to 10), and Day 21 (for Days 15 to 17), as applicable, of each treatment cycle and at the EOT visit to ensure compliance. For patients who do not require a clinic visit on Day 21, the medication count may be confirmed verbally with the patient by telephone at this visit; however, a check of the patient diary versus the patient's supply of MLN8237 tablets should be performed at the next visit (eg, on Day 1 of the subsequent cycle or at the EOT visit, if applicable).

7.5 Completion of Treatment

Treatment with MLN8237 is to be considered completed for patients meeting any of the following criteria:

- 24 months of treatment
- PD (see below for exception)
- Symptomatic deterioration

A patient can be treated for up to 24 months. If it is determined that a patient tolerates protocol treatment and would derive benefit from continued MLN8237 treatment beyond 24 months, MLN8237 treatment may be continued in this or in another extension or rollover study, if available, upon request by the investigator and agreement by the medical monitor.

A patient who experiences PD, as defined by CA-125 criteria or imaging results described in the protocol (Section 15.4) may continue protocol treatment if tolerated and if the investigator determines it is in the patient's best interest upon review and agreement by the medical monitor. Criteria for removing patients from the study include the following:

1. Patients with unequivocal evidence of any new metastasis including development of peritoneal studding or malignant ascites must be removed from study.
2. Patients whose disease progression is such that they are at risk for catastrophic complications from vital organ compression (eg, spinal cord compression, small bowel obstruction, encroachment of major blood vessels, etc.) must be removed from study immediately and treated appropriately.
3. Patients with evidence of progression based on RECIST must be removed from study if the subsequent assessment (8 weeks later) demonstrates evidence of progressive disease.
4. Patients must be informed of the evidence of progression and the availability of alternative treatments.

In addition to the above criteria, patients who have PD that is symptomatic or leads to altered organ function, such as increases in bilirubin by 1 CTC severity grade that could represent altered drug clearance, should be removed from study.

If a patient is maintained on study treatment after evidence for PD, serial objective monitoring will continue according to the Schedules of Events. Statistical analysis of

clinical outcomes (eg, time to progression) will continue to employ the predefined definitions for PD (RECIST or GCIG CA-125, Section 15.4) even if the patient continues protocol treatment after PD. In this setting, the date and criteria for the original determination of PD per protocol will be recorded in the CRF. The date and criteria for subsequent treatment discontinuation will be recorded in the CRF in addition to dosing history and serial evaluations of disease, including CA-125 and imaging results.

7.6 Discontinuation of Treatment With Study Drug, and Patient Replacement

For patients who do not complete treatment by meeting 1 or more of the criteria outlined in Section 7.5, treatment with study drug may also be discontinued for any of the following reasons:

- AE
- Withdrawal by patient
- Lost to follow-up
- Study terminated by sponsor
- Protocol violation
- Use of antineoplastic therapy other than study drug (MLN8237 or paclitaxel)

For determination of the MTD in Phase 1, patients will be replaced in a cohort if withdrawn from treatment during Cycle 1 for reasons other than DLT, including the following situations: failure to complete 1) at least 80% of the planned doses of MLN8237; or 2) at least 2 of the planned 3 doses of paclitaxel; or 3) the required safety evaluations scheduled in Cycle 1 to determine DLT.

At the time of discontinuation of study drug treatment, all study procedures outlined for the EOT visit should be completed. The primary reason for study drug discontinuation is to be recorded on the eCRF.

A patient who discontinues study treatment before the occurrence of PD will be followed off treatment for PD or death until the occurrence of 110 PFS events in the Phase 2 portion.

7.7 Withdrawal of Patients From Study Follow-Up

Patients who do not experience PD will be followed off treatment once every 8 weeks until the occurrence of 110 PFS events (PD or death, whichever occurs first) in the Phase 2

portion are documented for this study. The EOS visit will be completed at the time the patient completes or withdraws from the Follow-Up period.

For patients who enter the Follow-Up period of the study, the reasons for withdrawal from the study include:

- PD
- Withdrawal by patient
- Death
- Lost to follow-up
- Symptomatic deterioration
- Study terminated by sponsor
- Initiation of subsequent antineoplastic therapy

The consequence of withdrawal of all consent by a patient will be that no new information will be collected from that patient and added to the existing data or any database; however, every effort will be made to follow all patients for safety. The EOS visit will be completed at the time the patient withdraws from the Follow-Up period.

7.8 Study Compliance

Study drug will be administered or dispensed only to eligible patients under the supervision of the investigator or identified subinvestigator(s). The appropriate study personnel will maintain records of study drug receipt and dispensing, including the following: applicable lot numbers and total drug administered in milligrams (mg). Any discrepancy regarding the dose administered and the reason for the discrepancy will be noted in the eCRF.

Paclitaxel will be administered by study personnel.

Patients will receive a sufficient quantity of MLN8237 for each treatment cycle. The study center staff will check the patient's diary versus the patient's supply of remaining MLN8237 tablets at the Day 8, Day 15, and Day 21 visit of each treatment cycle and at the EOT visit to ensure proper compliance with dosing. For patients who do not require a clinic visit on Day 21, the medication count may be confirmed verbally with the patient by telephone; however, a check of the patient diary versus the patient's supply of MLN8237 tablets should be performed at the next visit (eg, on Day 1 of the subsequent cycle or at the EOT visit, if

applicable). Patients who are not compliant with the dosing schedule may be withdrawn from the study.

8. STATISTICAL AND QUANTITATIVE ANALYSES

8.1 Statistical Methods

Summary tabulations will be presented displaying the number of observations, mean, standard deviation, median, minimum, and maximum for continuous variables, and the number and percentage per category for categorical data. A formal statistical analysis plan will be developed and finalized before database lock.

For the Phase 1 portion, statistical analyses will be primarily descriptive and graphical in nature. No formal statistical hypothesis testing will be performed.

For the Phase 2 portion of the study only, time-to-event data will be analyzed by the Kaplan-Meier method and results will be summarized by 25th, 50th (median), and 75th percentiles with associated 2-sided 80% confidence intervals (CI), as well as the percentage of censored observations. Formal statistical hypothesis tests will be performed to test the superiority of MLN8237 plus weekly paclitaxel to weekly paclitaxel alone, with all tests conducted at the 1-sided 0.1 level of significance.

8.1.1 Determination of Sample Size

During the Phase 1 portion of the study, different doses of MLN8237 are planned for evaluation when combined with a fixed dose of paclitaxel to identify the RP2D. The dose of paclitaxel may be reduced depending on tolerability. Dose escalation will be conducted according to a traditional dose escalation rule, with 3 to 6 patients evaluated at each dose level. There will be an expansion cohort at the RP2D in order to have at least 12 patients evaluated at the RP2D. This Phase 1 portion of the study will include female patients with recurrent adenocarcinoma of the breast or epithelial adenocarcinoma of ovarian, tubal, or peritoneal origin, who are considered to be candidates for treatment with weekly paclitaxel. Including 10% of patients who are not evaluable for DLT, it is anticipated that enrollment of approximately 36 patients is needed.

The Phase 2 portion of the study is a randomized study to compare the efficacy and safety of the combination of MLN8237 plus weekly paclitaxel to weekly paclitaxel alone in patients with recurrent epithelial ovarian, fallopian tube, or peritoneal cancer. The primary efficacy endpoint of the Phase 2 portion is PFS. The primary efficacy analysis will compare PFS between the 2 treatment arms. Assuming the median PFS is 4 months for single-agent weekly paclitaxel, and MLN8237 plus weekly paclitaxel can improve the median PFS to 6 months (an approximately 33.3% reduction of hazard rates), a total of 110 events (PD or death, whichever occurs first) is needed for the 2 treatment arms in order to detect such an increase in median PFS from weekly paclitaxel alone to MLN8237 plus weekly paclitaxel (1-sided alpha is 0.1, and power is 80%). With 12 months of accrual, a dropout rate of about 10%, and a 1:1 randomization to treatment with either MLN8237 plus weekly paclitaxel or weekly paclitaxel alone, approximately 136 patients are needed for the Phase 2 portion of the study (approximately 68 patients per treatment arm). An interim analysis will be performed after the first 72 response-evaluable patients in the Phase 2 portion have either completed a minimum of 2 cycles of therapy or have discontinued study treatment. The interim analysis will be based on the CBR, which includes the best overall combined response and SD as shown in Table 8-1, and a patient must have at least 2 continuous assessments of SD to be counted. The study will be terminated if the CBR of the MLN8237 plus weekly paclitaxel arm is at least 10% lower than that of the weekly paclitaxel alone arm.

Overall, enrollment of approximately 172 patients is needed for this study.

8.1.2 Randomization and Stratification

In the Phase 1 portion of the study, there is no randomization or stratification; all patients will receive MLN8237 plus weekly paclitaxel.

In the Phase 2 portion of the study, patients will be randomized in a 1:1 ratio to open-label treatment with either MLN8237 plus weekly paclitaxel or weekly paclitaxel alone. This study will use a central (non-center-specific) randomization scheme with the following stratification factors: months of relapse since the prior platinum therapy (refractory; 0 to 6 months; or 6 to 12 months); and prior weekly taxane treatment (yes or no).

8.1.3 Populations for Analysis

The populations used for analysis will include the following:

- Safety population: the safety population, defined as all patients who receive at least 1 dose of any study drug, will be used for all safety analyses
- MLN8237 PK evaluable population: The population of patients evaluable for the determination of the PK of MLN8237 is defined as all patients in the Phase 1 portion of the study for whom there are sufficient dosing and MLN8237 concentration-time data to permit noncompartmental PK analysis.
- Paclitaxel PK evaluable population: The population of patients evaluable for the determination of the PKof paclitaxel's is defined as all patients in the Phase 1 portion of the study for whom there are sufficient dosing and paclitaxel concentration-time data to permit noncompartmental PK analysis.
- DLT-evaluable population: the DLT-evaluable population, defined as all patients in the Phase 1 portion of the study who either experience DLT during Cycle 1 or complete treatment with at least 80% of the planned doses of MLN8237 and 2 of the planned 3 doses of paclitaxel in Cycle 1 and have sufficient follow-up data to allow the investigators and sponsor to determine whether DLT occurred, will be used for analysis of DLT
- Modified Intent-to-Treat (mITT) population: the mITT population, defined as all patients who are randomized and receive at least 1 dose of any study drug, will be used for the analyses of PFS, TTP, and OS
- Response-evaluable population: the response-evaluable population, defined as all patients who are randomized and have measurable disease according to RECIST, version 1.1 or assessable disease by CA-125 criteria, who have received at least 1 dose of any study drug, and who have at least 1 available postbaseline response assessment as per either RECIST, version 1.1 or CA-125 criteria, will be used for analyses of ORR and DOR

8.1.4 Procedures for Handling Missing, Unused, and Spurious Data

All available data will be included in data listings. No imputation of values for missing data will be performed. Data that are potentially spurious or erroneous will be examined according to standard data management operating procedures.

8.1.5 Demographic and Baseline Characteristics

The demographic and baseline characteristics will be summarized in a descriptive fashion. Data to be evaluated will include age, race, ethnicity, and other parameters, as appropriate.

8.1.6 Efficacy Analysis

8.1.6.1 Phase 1 Portion of the Study

Primary Efficacy

There is no primary efficacy endpoint in the Phase 1 portion of the study.

Secondary Efficacy

The secondary efficacy endpoint in the Phase 1 portion of the study is the best overall combined response as defined in Table 8-1, which will be presented in a listing.

8.1.6.2 Phase 2 Portion of the Study

Primary Efficacy

The primary endpoint for the Phase 2 portion is PFS, defined as the time from the date of randomization to the date of first documentation of progression or death due to any cause, whichever occurs first.

The primary efficacy analysis will be based on the mITT population. The Kaplan-Meier method will be used to analyze the distribution of PFS for each of the 2 treatment arms. Estimates from the Kaplan-Meier analysis, hazard ratio, and 80% CI will be presented. An unstratified log-rank test will be performed between the PFS of the 2 arms, and if the p-value is ≤ 0.1, then the combination will be considered worthy of additional clinical evaluation.

Secondary Efficacy

The secondary efficacy endpoints in the Phase 2 portion of the study include ORR, DOR, TTP, and OS.

ORR is the best overall combined response rate, where best overall combined response is defined as in Table 8-1. The estimate of the ORR will be presented with 2-sided 80% exact binomial CI for each treatment arm. The number and percentage of patients in each response category will be tabulated for each treatment arm based on RECIST, version 1.1, only and also based on CA-125 criteria only. Fisher's exact tests will be performed for the comparison of ORR between the treatment arms. The analyses will be based on the response-evaluable population.

Table 8-1 Best Overall Combined Response

RECIST	CA-125	Overall
CR	Response/PD/SD/(N/A)	CR
PR	Response/PD/SD/(N/A)	PR
PD	Response/PD/SD/(N/A)	PD
SD	Response	Response
SD	PD	PD
SD	SD/(N/A)	SD
N/A	Response	Response
N/A	SD	SD
N/A	PD	PD

Abbreviations: CR = complete response; N/A = not assessed; PD = progressive disease, disease progression; PR = partial response; SD = stable disease.

DOR is defined as the time from the date of first documentation of a response to the date of first documentation of objective progression.

TTP is defined as the time from the date of randomization to the date of first documentation of objective progression.

OS is defined as the time from the date of randomization to the date of death.

DOR, TTP, and OS will be analyzed using the Kaplan-Meier method. Unstratified log-rank tests will be performed for the comparisons between the 2 treatment arms. DOR will be analyzed based on the responders in the response-evaluable population. TTP and OS will be analyzed based on the mITT population.

8.1.7 Pharmacokinetics/Pharmacodynamics/Biomarkers

Pharmacokinetic Analysis

Paclitaxel

For the Phase 1 portion of the study, individual and mean plasma concentration data will be plotted over time for paclitaxel alone (Cycle 2, Day 1) and for paclitaxel administered concomitantly with MLN8237 (Cycle 1, Day 1). Noncompartmental PK analysis will be performed on individual concentration-time data to calculate plasma PK parameters, including, but not limited to, C_{max}, $AUC_{0\text{-tlast}}$, $AUC_{0\text{-}\infty}$, and $t_{½}$, for paclitaxel administered alone (Cycle 2, Day 1) and during concomitant administration of MLN8237 (Cycle 1,

Day 1). Descriptive statistics will be presented for plasma PK parameters. Additionally, the ratio of geometric means of paclitaxel C_{max}, $AUC_{0\text{-tlast}}$, and $AUC_{0\text{-}\infty}$ (when administered with MLN8237 in reference to when administered alone) and the associated 90% CI will be calculated.

MLN8237

For the Phase 1 portion of this study, individual and mean plasma concentration-time data will be plotted for MLN8237 on Days 1 and 3. Noncompartmental PK analysis will be performed on individual concentration-time data to calculate plasma PK parameters of MLN8237, including but not limited to, Day 1 and Day 3 C_{max}, T_{max}, and $AUC_{0\text{-}\tau}$. MLN8237 plasma concentration-time data and PK parameters will be summarized descriptively using the PK-evaluable population.

Individual concentration-time data collected in the Phase 2 portion of this study will be tabulated and may contribute to population PK analyses as discussed in Section 8.2.

Pharmacodynamic Analysis

This is not applicable to this study.

Biomarkers

For the exploratory endpoints, descriptive statistics, graphical methods, and statistical modeling, whichever is appropriate, may be used to explore the relationship between response to treatments and (1) levels of various biomarkers, [redacted] and PK parameters may be analyzed in this study, as well as part of the population PK analysis across multiple studies.

8.1.8 Safety Analysis

Safety evaluations will be based on the incidence, intensity, and type of AEs; and clinically significant changes in the patient's vital signs, weight, and clinical laboratory results. Safety variables will be tabulated and presented for the safety population. The incidence of DLT

will be presented. Exposure to study drug and reasons for discontinuation of study treatment will be tabulated.

AEs will be coded using the Medical Dictionary for Regulatory Activities (MedDRA) coding dictionary for the purpose of summarization. All AEs occurring on study will be listed in data listings. Treatment-emergent AEs will be tabulated, where treatment-emergent is defined as any AE that occurs after administration of the first dose of any study drug through 30 days after the last dose of any study drug or until the start of subsequent antineoplastic therapy, whichever occurs first, any event that is considered by the investigator to be drug-related regardless of the start date of the event, or any event that is present at baseline but worsens in intensity or is subsequently considered by the investigator to be drug-related. AEs are to be tabulated using MedDRA system organ class, high-level term, and preferred term, including the following treatment-emergent categories:

- Treatment-emergent AEs
- Drug-related treatment-emergent AEs
- Grade 3 or higher treatment-emergent AEs
- Grade 3 or higher drug-related treatment-emergent AEs
- Treatment-emergent AEs resulting in study drug discontinuation
- SAEs

The most commonly reported treatment-emergent AEs (ie, those events reported by ≥ 10% of all patients in the safety population) will be tabulated by the MedDRA preferred term. Tabulation also will be provided that enumerates AEs by maximum intensity. Deaths, SAEs, and AEs resulting in study drug discontinuation will be tabulated.

Clinical laboratory parameters will be summarized at each scheduled time point. Shift tables will be produced for selected laboratory parameters. These tables will summarize the number of patients with each baseline NCI CTCAE grade and changes to the worst NCI CTCAE grade during study.

Descriptive statistics for the actual values of vital signs and weight over time will be tabulated by scheduled time point.

All concomitant medications collected from screening through the study period will be classified by preferred term according to the WHO drug dictionary.

Additional safety analyses may be determined at any time without prejudice, in order to enumerate rates of toxicities and to further define the safety profile of study drugs.

8.1.9 Interim Analysis

The interim futility analysis for the Phase 2 portion of the study will be performed after the first 72 response-evaluable patients (approximately 36 per treatment arm) in the Phase 2 portion have either completed a minimum of 2 cycles of therapy or have discontinued study treatment. This interim analysis will be based on the CBR, which includes the best overall combined response and SD as shown in Table 8-1, and a patient must have 2 continuous assessments of SD to be counted. The study will be terminated if the absolute CBR of the MLN8237 plus weekly paclitaxel arm is at least 10% lower than the absolute CBR of the weekly paclitaxel alone arm; ie, the study will be terminated if (x% CBR of paclitaxel) - (y% CBR of the MLN8237 plus weekly paclitaxel) < 10%; otherwise, the study will continue.

At the interim analysis, the planned sample size may be assessed with regard to the data accrued at that time. If the interim analysis indicates that the current sample size may not yield sufficient power, and the review of the safety data indicates adequate tolerance to the treatment, the planned sample size may be modified after the interim analysis for continuation of the study and the final analysis. If sample size re-estimation occurs, the type I error rate will be adjusted accordingly to ensure statistical rigor.

8.2 Pharmacokinetic Modeling

In addition to the noncompartmental PK analyses, the PK data collected during this study (including data collected from patients undergoing sparse PK sampling in the Phase 2 portion) together with previously collected PK data in MLN8237 clinical studies, as well as PK data that may be collected in future studies, may be utilized for population PK and exposure-response analyses of selected efficacy or safety endpoints, or both. The results of these analyses may serve to further characterize MLN8237 PK, understand potential sources of variation, including patient-specific factors (eg, age, sex, renal and hepatic function, etc), and enable exploratory analyses of relationships between PK and drug effects (clinical response to treatment or AEs, or both). The specifics of the modeling approaches for these analyses will be described in a population PK analysis plan and the results reported separately.

9. STUDY COMMITTEES

9.1 Safety Monitoring Committee

An internal SMC will review available safety data on a periodic basis throughout the conduct of the study. The SMC will include physicians from the study team and Pharmacovigilance, the medical monitor or designee, as well as staff from Pharmacovigilance, Biostatistics, and Clinical Operations groups. The SMC also will review the safety and other clinical data from the Phase 1 portion, coupled with consultation with the Phase 1 investigators, to determine the RP2D of the MLN8237 plus weekly paclitaxel regimen to be used in the Phase 2 portion. As the study does not include blinding or early stopping for efficacy, the major focus of the SMC will be on patient safety as described for an Internal Safety Review Committee.[(45)]

10. ADVERSE EVENTS

10.1 Definitions

10.1.1 Pretreatment Event Definition

A pretreatment event is any untoward medical occurrence in a patient or subject who has signed informed consent to participate in a study but before administration of any study medication; it does not necessarily have to have a causal relationship with study participation.

10.1.2 Adverse Event Definition

Adverse event (AE) means any untoward medical occurrence in a patient or subject administered a pharmaceutical product; the untoward medical occurrence does not necessarily have to have a causal relationship with this treatment. An AE can therefore be any unfavorable and unintended sign (including an abnormal laboratory finding, for example), symptom, or disease temporally associated with the use of a medicinal product whether or not it is considered to be related to the medicinal product. This includes any newly occurring event, or a previous condition that has increased in severity or frequency since the administration of study drug.

An abnormal laboratory value will not be assessed as an AE unless that value leads to discontinuation or delay in treatment, dose modification, therapeutic intervention, or is considered by the investigator to be a clinically significant change from baseline.

10.1.3 Serious Adverse Event Definition

Serious AE (SAE) means any untoward medical occurrence that at any dose:

- Results in **death.**
- Is **life-threatening**. (The term "life-threatening" in the definition of "serious" refers to an event in which the patient was at risk of death at the time of the event; it does not refer to an event which hypothetically might have caused death if it were more severe).
- Requires inpatient **hospitalization or prolongation of present hospitalization** (see clarification in the paragraph below on planned hospitalizations).
- Results in **persistent or significant disability/incapacity**.
- Is a **congenital anomaly/birth defect**.
- Is a **medically important event** that may not be immediately life threatening or result in death or hospitalization but may jeopardize the patient or may require intervention to prevent 1 of the other outcomes listed in the definition above, or involves suspected transmission via a medicinal product of an infectious agent. Examples of such medical events include allergic bronchospasm requiring intensive treatment in an emergency room or at home, blood dyscrasias or convulsions that do not result in inpatient hospitalization, or the development of drug dependency or drug abuse; any organism, virus, or infectious particle (eg, prion protein transmitting Transmissible Spongiform Encephalopathy), pathogenic or nonpathogenic, is considered an infectious agent.

In this study, intensity for each AE, including any lab abnormality, will be determined using the NCI CTCAE, Version 4.02, effective as of 01 October 2009. Clarification should be made between a serious AE (SAE) and an AE that is considered severe in intensity (Grade 3 or 4), because the terms serious and severe are NOT synonymous. The general term *severe* is often used to describe the intensity (severity) of a specific event; the event itself, however, may be of relatively minor medical significance (such as a Grade 3 headache). This is NOT

the same as *serious*, which is based on patient/event outcome or action criteria described above and is usually associated with events that pose a threat to a patient's life or ability to function. A severe AE (Grade 3 or 4) does not necessarily need to be considered serious. For example, a Leukocyte value of 1000-2000/mm^3 is considered Grade 3 (severe) but may not be considered serious. Seriousness (not intensity) serves as a guide for defining regulatory reporting obligations.

10.2 Procedures for Recording and Reporting Adverse Events and Serious Adverse Events

All AEs spontaneously reported by the patient and/or in response to an open question from study personnel or revealed by observation, physical examination, or other diagnostic procedures will be recorded on the appropriate page of the eCRF (see Section 10.3 for the period of observation). Any clinically relevant deterioration in laboratory assessments or other clinical finding is considered an AE and must be recorded on the appropriate pages of the eCRF. When possible, signs and symptoms indicating a common underlying pathology should be noted as 1 comprehensive event.

All SAEs and serious pretreatment events (as defined in Section 10.1.1) must be reported (see Section 10.3 for the period of observation) by the investigator to the Millennium Department of Pharmacovigilance or designee (contact information provided below) by faxing the SAE Form within 1 working day after becoming aware of the event. All SAEs and serious pretreatment events (which include all deaths) must be reported whether or not considered causally related to the study drug or study procedures. The SAE Form, created specifically by Millennium, will be provided to each clinical study site. A sample of the SAE Form may be found in the Study Manual. Follow-up information on the SAE or serious pretreatment event may be requested by Millennium. SAE report information must be consistent with the data provided on the eCRF.

**SAE Reporting Contact Information: North America**<br>[redacted]<br>Safety and Medical Management, US<br>Fax: [redacted]<br>Hotline number (available 24/7): [redacted]<br><br>**SAE Reporting Contact Information: Europe**<br>[redacted]<br>Safety and Medical Management,<br>Fax: [redacted]<br>Hotline number (available 24/7): [redacted]

Planned hospital admissions or surgical procedures for an illness or disease that existed before the patient was enrolled in the study are not to be considered AEs unless the condition deteriorated in an unexpected manner during the study (eg, surgery was performed earlier or later than planned).

For both serious and nonserious AEs, the investigator must determine both the intensity of the event and the relationship of the event to administration of study drug(s). For serious pretreatment events, the investigator must determine both the intensity of the event and the relationship of the event to study procedures.

Intensity for each AE, including any laboratory abnormality, will be determined using the NCI CTCAE, Version 4.02, effective as of 01 October 2009. The criteria are provided in the Study Manual and also are available online at http://ctep.cancer.gov/reporting/ctc.html.

Relationship to administration of study drug(s) will be determined by the investigator responding yes or no to this question: Is there a reasonable possibility that the AE is associated with the study drug(s)? The investigator can segregate the causal relationship in the AE report form.

10.3 Monitoring of Adverse Events and Period of Observation

AEs, both nonserious and serious (which include all deaths), will be monitored throughout the study as follows:

- AEs will be reported from the time of the first dose of study drug through 30 days after the last dose of study drug or until the start of subsequent antineoplastic

therapy, whichever occurs first. That is, if a patient begins a new antineoplastic therapy, the AE reporting period for nonserious AEs ends at the time the new treatment is started.

- Serious pretreatment events will be reported to Millennium Pharmacovigilance or designee from the time of the signing of the ICF up to first dose of study drug, but will not be recorded in the eCRF.
- SAEs will be reported to Millennium Pharmacovigilance or designee from the first dose of study drug through 30 days after administration of the last dose of study drug and recorded in the eCRF. All SAEs (which include all deaths) must also be reported to the Millennium Department of Pharmacovigilance or designee. All SAEs should be monitored until they are resolved or are clearly determined to be due to a patient's stable or chronic condition or intercurrent illness(es). Any SAE that occurs at any time after completion of the study and the designated follow-up period that the investigator considers to be related to study drug must be reported to the Millennium Department of Pharmacovigilance.

10.4 Procedures for Reporting Drug Exposure During Pregnancy and Birth Events

If a woman becomes pregnant or suspects that she is pregnant while participating in this study, she must inform the investigator immediately and permanently discontinue study drug. The sponsor must also be contacted immediately by faxing a completed Pregnancy Form to the Millennium Department of Pharmacovigilance (see Section 10.2). The pregnancy must be followed for the final pregnancy outcome.

11. ADMINISTRATIVE REQUIREMENTS

11.1 Good Clinical Practice

The study will be conducted in accordance with the ICH-GCP and the appropriate regulatory requirement(s). The investigator will be thoroughly familiar with the appropriate use of the study drug as described in the protocol and the IB.

11.2 Reporting of Suspected Unexpected Serious Adverse Reactions

The sponsor will expedite the reporting of suspected unexpected serious adverse reactions (SUSARs) to concerned regulatory authorities, ethics committees, and investigators in

accordance with all relevant laws and regulations governing the reporting of adverse drug reactions from clinical studies. For purposes of regulatory reporting, expectedness will be based on the sponsor's IB.

AEs described as disease progression by the investigator will be excluded from the sponsor's expedited reporting to health authorities.

11.3 Data Quality Assurance

The investigator is required to prepare and maintain adequate and accurate case histories designed to record all observations and other data pertinent to the study for each study patient. Study data will be entered into an eCRF by site personnel using a secure, validated, web-based electronic data capture (EDC) application. Millennium will have access to all data upon entry in the EDC application.

Study monitors will discuss instances of missing or uninterpretable data with the investigator for resolution. Any changes to study data will be made to the eCRF and documented via an electronic audit trail associated with the affected eCRF.

11.4 Electronic Case Report Form Completion

Millennium or designee will provide the study sites with secure access to and training on the EDC application, sufficient to permit site personnel to enter or correct information in the eCRFs for the patients for whom they are responsible.

eCRFs will be completed for each study patient. It is the investigator's responsibility to ensure the accuracy, completeness, clarity, and timeliness of the data reported in the patient's eCRF.

The investigator, or designated representative, should complete the eCRF as soon as possible after information is collected.

The investigator must provide through the EDC application formal approval of all the information in the eCRFs and changes to the eCRFs to endorse the final submitted data for the patients for which he or she is responsible. The audit trail entry will show the user's identification information and the date and time of the correction.

Millennium, or a designee, will retain the eCRF data and corresponding audit trails. A copy of the final archival eCRF in the form of a CD or other electronic media will be placed in the investigator's study file.

11.5 Study Monitoring

Monitoring and auditing procedures developed or approved by Millennium will be followed to comply with GCP guidelines.

All information recorded on the eCRFs for this study must be consistent with the patient's source documentation. During the course of the study, the study monitor will make study site visits to review protocol compliance, verify eCRFs against source documentation, assess drug accountability, and ensure that the study is being conducted according to pertinent regulatory requirements. The review of medical records will be performed in a manner that ensures that patient confidentiality is maintained.

11.6 Ethical Considerations

The study will be conducted in accordance with ethical principles founded in the Declaration of Helsinki. The IRB/IEC will review all appropriate study documentation to safeguard the rights, safety, and well-being of the patients. The study will be conducted only at sites where IRB/IEC approval has been obtained. The protocol, IB, ICF, advertisements (if applicable), written information given to the patients (including diary cards), safety updates, annual progress reports, and any revisions to these documents will be provided to the IRB/IEC by the investigator or the sponsor, as allowed by local regulations.

11.7 Patient Information and Informed Consent

After the study has been fully explained, written informed consent will be obtained from either the patient or his/her guardian or legal representative before study participation. The method of obtaining and documenting the informed consent and the contents of the consent must comply with the ICH-GCP and all applicable regulatory requirements.

11.8 Patient Confidentiality

To maintain patient privacy, all eCRFs, study drug accountability records, study reports, and communications will identify the patient by initials where permitted and/or by the assigned patient number. The patient's confidentiality will be maintained and will not be made publicly available to the extent permitted by the applicable laws and regulations.

11.9 Investigator Compliance

The investigator will conduct the study in compliance with the protocol provided by Millennium and given approval/favorable opinion by the IRB/IEC and the appropriate regulatory authority(ies). Modifications to the protocol are not to be made without agreement of both the investigator and Millennium. Changes to the protocol will require written IRB/IEC approval/favorable opinion before implementation, except when the modification is needed to eliminate an immediate hazard or hazards to patients. Millennium, or a designee, will submit all protocol modifications to the appropriate regulatory authority(ies) in accordance with the governing regulations.

When immediate deviation from the protocol is required to eliminate an immediate hazard or hazards to patients, the investigator will contact Millennium, or a designee, if circumstances permit, to discuss the planned course of action. Any departures from the protocol must be documented.

11.10 On-site Audits

Regulatory authorities, the IEC/IRB, and/or Millennium may request access to all source documents, eCRFs, and other study documentation for on-site audit or inspection. Direct access to these documents must be guaranteed by the investigator, who must provide support at all times for these activities.

11.11 Investigator and Site Responsibility for Drug Accountability

Accountability for the study drug at the study site is the responsibility of the investigator. Drug accountability records indicating the drug's delivery date to the site, inventory at the site, use by each patient, and amount returned to Millennium, or a designee (or disposal of the drug, if approved by Millennium) will be maintained by the clinical site. Millennium or its designee will review drug accountability at the site on an ongoing basis.

All material containing study drug will be treated and disposed of in accordance with governing regulations.

11.12 Product Complaints and Medication Errors

A product complaint is a verbal, written, or electronic expression that implies dissatisfaction regarding the identity, strength, purity, quality, or stability of a drug product. Individuals who identify a potential product complaint situation should immediately contact [redacted]

[redacted] (see below) and report the event. Whenever possible, the associated product should be maintained in accordance with the label instructions pending further guidance from a Millennium quality representative.

A medication error is a preventable event that involves an identifiable patient and that leads to inappropriate medication use, which may result in patient harm. While overdoses and underdoses constitute medication errors, doses missed inadvertently by a patient do not. Individuals who identify a potential medication error situation should immediately contact [redacted] (see below) and report the event.

For Product Complaints or Medication Errors,<br>call [redacted] at<br>[redacted]<br>(US and International)

Product complaints and medication errors in and of themselves are not AEs. If a product complaint or medication error results in an SAE, an SAE form should be completed and sent to [redacted] (refer to Section 10.2).

11.13 Closure of the Study

The sponsor will notify the competent authorities and the IECs in all member states where the study is being carried out that the study has ended as required by regulation.

Within 1 year of the end of the study, a summary of the clinical study results will be submitted to the competent authorities and IECs in all member states involved in the study.

Study participation by individual sites or the entire study may be prematurely terminated if, in the opinion of the investigator or Millennium, there is sufficient reasonable cause. Written notification documenting the reason for study termination will be provided to the investigator or Millennium by the terminating party.

Circumstances that may warrant termination include, but are not limited to:

- Determination of unexpected, significant, or unacceptable risk to patients
- Failure to enter patients at an acceptable rate
- Insufficient adherence to protocol requirements
- Insufficient, incomplete, and/or unevaluable data

- Determination of efficacy based on interim analysis
- Plans to modify, suspend or discontinue the development of the study drug

Should the study be closed prematurely, the site will no longer be able to access the EDC application, will not have a right to use the EDC application, and will cease using the password or access materials once their participation in the study has concluded. In the event that any access devices for the EDC application have been provided, these will be returned to Millennium once the site's participation in the study has concluded.

Within 15 days of premature closure, Millennium must notify the competent authorities and IECs of any member state where the study is being conducted, providing the reasons for study closure.

11.14 Record Retention

The investigator will maintain all study records according to the ICH-GCP and applicable regulatory requirement(s). Records will be retained for at least 2 years after the last marketing application approval or 2 years after formal discontinuation of the clinical development of the investigational product or according to applicable regulatory requirement(s). If the investigator withdraws from the responsibility of keeping the study records, custody must be transferred to a person willing to accept the responsibility and Millennium notified.

12. USE OF INFORMATION

All information regarding MLN8237 supplied by Millennium to the investigator is privileged and confidential information. The investigator agrees to use this information to accomplish the study and will not use it for other purposes without consent from Millennium. It is understood that there is an obligation to provide Millennium with complete data obtained during the study. The information obtained from the clinical study will be used toward the development of MLN8237 and may be disclosed to regulatory authority(ies), other investigators, corporate partners, or consultants as required.

Upon completion of the clinical study and evaluation of results by Millennium, the hospital or institution and/or investigator may publish or disclose the clinical study results pursuant to the terms contained in the applicable Clinical Study Agreement.

13. INVESTIGATOR AGREEMENT

I have read Protocol C14008 Amendment 3: Randomized Phase 2 Study of MLN8237, an Aurora A Kinase Inhibitor, Plus Weekly Paclitaxel or Weekly Paclitaxel Alone in Patients With Recurrent Epithelial Ovarian, Fallopian Tube, or Primary Peritoneal Cancer, Preceded by a Phase 1 Portion in Patients With Ovarian or Breast Cancer.

I agree to conduct the study as detailed herein and in compliance with International Conference on Harmonisation Guidelines for Good Clinical Practice and applicable regulatory requirements and to inform all who assist me in the conduct of this study of their responsibilities and obligations.

____
Principal investigator printed name

____ ____________________
Principal investigator signature Date

____

____

____
Investigational site or name of institution and location (printed)

14. REFERENCES

1. Bast RC, Jr., Mills GB. Personalizing therapy for ovarian cancer: BRCAness and beyond. J Clin Oncol 2010;28(22):3545-8.
2. Sourisseau T, Maniotis D, McCarthy A, Tang C, Lord CJ, Ashworth A, et al. Aurora-A expressing tumour cells are deficient for homology-directed DNA double strand-break repair and sensitive to PARP inhibition. EMBO molecular medicine 2010;2(4):130-42.
3. Matulonis U, Sharma S, Ghamande S, Gordon M, Del Prete S, Ray-Coquard I, et al. Single-agent activity and safety of the investigational Aurora A kinase inhibitor MLN8237 in patients with platinum-treated epithelial ovarian, fallopian tube, or primary peritoneal carcinoma. In: European Society for Medical Oncology (ESMO); 2010 8-12 October; Milan, Italy.
4. Huck JJ, Zhang M, McDonald A, Bowman D, Hoar KM, Stringer B, et al. MLN8054, an inhibitor of Aurora A kinase, induces senescence in human tumor cells both in vitro and in vivo. Mol Cancer Res 2010;8(3):373-84.
5. Jemal A, Siegel R, Ward E, Murray T, Xu J, Thun MJ. Cancer Statistics, 2007. CA Cancer J Clin 2007;57(1):43-66.
6. Markman M, Rothman R, Hakes T, Reichman B, Hoskins W, Rubin S, et al. Second-line platinum therapy in patients with ovarian cancer previously treated with cisplatin. J Clin Oncol 1991;9(3):389-93.
7. Nguyen TT, Wright JD, Powell MA, Gibb RK, Rader JS, Allsworth JE, et al. Prognostic factors associated with response in platinum retreatment of platinum-resistant ovarian cancer. Int J Gynecol Cancer 2008;18(6):1194-9.
8. Harrison ML, Gore ME, Spriggs D, Kaye S, Iasonos A, Hensley M, et al. Duration of second or greater complete clinical remission in ovarian cancer: exploring potential endpoints for clinical trials. Gynecol Oncol 2007;106(3):469-75.
9. Markman M, Markman J, Webster K, Zanotti K, Kulp B, Peterson G, et al. Duration of response to second-line, platinum-based chemotherapy for ovarian cancer: implications for patient management and clinical trial design. J Clin Oncol 2004;22(15):3120-5.
10. Eniu A, Palmieri FM, Perez EA. Weekly administration of docetaxel and paclitaxel in metastatic or advanced breast cancer. Oncologist 2005;10(9):665-85.
11. Carvajal RD, Tse A, Schwartz GK. Aurora kinases: new targets for cancer therapy. Clin Cancer Res 2006;12(23):6869-75.

12. Lu LY, Wood JL, Ye L, Minter-Dykhouse K, Saunders TL, Yu X, et al. Aurora A is essential for early embryonic development and tumor suppression. J Biol Chem 2008;283(46):31785-90.

13. Lin YG, Immaneni A, Merritt WM, Mangala LS, Kim SW, Shahzad MM, et al. Targeting aurora kinase with MK-0457 inhibits ovarian cancer growth. Clin Cancer Res 2008;14(17):5437-46.

14. Landen CN, Jr., Lin YG, Immaneni A, Deavers MT, Merritt WM, Spannuth WA, et al. Overexpression of the centrosomal protein Aurora-A kinase is associated with poor prognosis in epithelial ovarian cancer patients. Clin Cancer Res 2007;13(14):4098-104.

15. Anand S, Penrhyn-Lowe S, Venkitaraman AR. AURORA-A amplification overrides the mitotic spindle assembly checkpoint, inducing resistance to Taxol. Cancer Cell 2003;3(1):51-62.

16. Yang H, He L, Kruk P, Nicosia SV, Cheng JQ. Aurora-A induces cell survival and chemoresistance by activation of Akt through a p53-dependent manner in ovarian cancer cells. Int J Cancer 2006;119(10):2304-12.

17. Infante J. Phase I study of the safety, pharmacokinetics (PK), and pharmacodynamics (PD) of MLN8237, a selective Aurora A kinase inhibitor, in the United States. Eur J Cancer 2008:EORTC Abstract 280.

18. Gynecologic Oncology G, Markman M, Blessing J, Rubin SC, Connor J, Hanjani P, et al. Phase II trial of weekly paclitaxel (80 mg/m2) in platinum and paclitaxel-resistant ovarian and primary peritoneal cancers: a Gynecologic Oncology Group study. Gynecol Oncol 2006;101(3):436-40.

19. Rosenberg P, Andersson H, Boman K, Ridderheim M, Sorbe B, Puistola U, et al. Randomized trial of single agent paclitaxel given weekly versus every three weeks and with peroral versus intravenous steroid premedication to patients with ovarian cancer previously treated with platinum. Acta Oncol 2002;41(5):418-24.

20. Katsumata N, Yasuda M, Takahashi F, Isonishi S, Jobo T, Aoki D, et al. Dose-dense paclitaxel once a week in combination with carboplatin every 3 weeks for advanced ovarian cancer: a phase 3, open-label, randomised controlled trial. Lancet 2009;374(9698):1331-8.

21. Linch M, Stavridi F, Hook J, Barbachano Y, Gore M, Kaye SB. Experience in a UK cancer centre of weekly paclitaxel in the treatment of relapsed ovarian and primary peritoneal cancer. Gynecol Oncol 2008;109(1):27-32.

22. Havrilesky LJ, Alvarez AA, Sayer RA, Lancaster JM, Soper JT, Berchuck A, et al. Weekly low-dose carboplatin and paclitaxel in the treatment of recurrent ovarian and peritoneal cancer. Gynecol Oncol 2003;88(1):51-7.

23. Bulychev A. An evaluation of the pharmacokinetics of MLN8237-001 after oral administration of prototype clinical formulations of MLN8237-004 to non-naive male beagle dogs. Cambridge (MA): Millennium Pharmaceuticals, Inc.; 2008. Report No.: RPT-01132.

24. Dees E, Infante J, Burris H, Astsaturov I, Stinchcombe T, Liu H, et al. Phase I study of the investigational drug MLN8237, an Aurora A kinase (AAK) inhibitor, in patients (pts) with solid tumors. J Clin Oncol (ASCO Meeting Abstracts) 2010;28(7s):abstr 3010.

25. Cervantes A, Burris H, Cohen R, Dees E, Infante J, Fingert H. Pharmacokinetic (PK) and pharmacodynamic (PD) results from 2 phase 1 studies of the investigational selective Aurora A kinase (AAK) inhibitor MLN8237: exposure-dependent AAK inhibition in human tumors. J Clin Oncol (ASCO Meeting Abstracts) 2010;28(7s):abstr 3031.

26. Cervantes-Ruiperez A, Elez M, Rosello S, Macarulla T, Rodriguez-Braun E, Lee Y, et al. Phase I pharmacokinetic (PK) and pharmacodynamic (PD) study of MLN8237, a novel selective aurora A kinase (AAK) inhibitor, in patients (pts) with advanced solid tumors. J Clin Oncol (ASCO Meeting Abstracts) 2009;27(15s):abstr 2565.

27. Scharer CD, Laycock N, Osunkoya AO, Logani S, McDonald JF, Benigno BB, et al. Aurora kinase inhibitors synergize with paclitaxel to induce apoptosis in ovarian cancer cells. J Transl Med 2008;6:79.

28. Sen S, Zhou H, Zhang RD, Yoon DS, Vakar-Lopez F, Ito S, et al. Amplification/overexpression of a mitotic kinase gene in human bladder cancer. J Natl Cancer Inst 2002;94(17):1320-9.

29. Carter SL, Eklund AC, Kohane IS, Harris LN, Szallasi Z. A signature of chromosomal instability inferred from gene expression profiles predicts clinical outcome in multiple human cancers. Nat Genet 2006;38(9):1043-8.

30. Ewart-Toland A, Briassouli P, de Koning JP, Mao JH, Yuan J, Chan F, et al. Identification of Stk6/STK15 as a candidate low-penetrance tumor-susceptibility gene in mouse and human. Nat Genet 2003;34(4):403-12.

31. Sankaran S, Crone DE, Palazzo RE, Parvin JD. Aurora-A kinase regulates breast cancer associated gene 1 inhibition of centrosome-dependent microtubule nucleation. Cancer Res 2007;67(23):11186-94.

32. Ouchi M, Fujiuchi N, Sasai K, Katayama H, Minamishima YA, Ongusaha PP, et al. BRCA1 phosphorylation by Aurora-A in the regulation of G2 to M transition. J Biol Chem 2004;279(19):19643-8.

33. Ferrandina G, Zannoni GF, Martinelli E, Paglia A, Gallotta V, Mozzetti S, et al. Class III beta-tubulin overexpression is a marker of poor clinical outcome in advanced ovarian cancer patients. Clin Cancer Res 2006;12(9):2774-9.

34. Hayashi Y, Kuriyama H, Umezu H, Tanaka J, Yoshimasu T, Furukawa T, et al. Class III beta-tubulin expression in tumor cells is correlated with resistance to docetaxel in patients with completely resected non-small-cell lung cancer. Intern Med 2009;48(4):203-8.

35. Aoki D, Oda Y, Hattori S, Taguchi K, Ohishi Y, Basaki Y, et al. Overexpression of class III beta-tubulin predicts good response to taxane-based chemotherapy in ovarian clear cell adenocarcinoma. Clin Cancer Res 2009;15(4):1473-80.

36. Cummings J, Hodgkinson C, Odedra R, Sini P, Heaton SP, Mundt KE, et al. Preclinical evaluation of M30 and M65 ELISAs as biomarkers of drug induced tumor cell death and antitumor activity. Mol Cancer Ther 2008;7(3):455-63.

37. Olofsson MH, Ueno T, Pan Y, Xu R, Cai F, van der Kuip H, et al. Cytokeratin-18 is a useful serum biomarker for early determination of response of breast carcinomas to chemotherapy. Clin Cancer Res 2007;13(11):3198-206.

38. Kramer G, Erdal H, Mertens HJ, Nap M, Mauermann J, Steiner G, et al. Differentiation between cell death modes using measurements of different soluble forms of extracellular cytokeratin 18. Cancer Res 2004;64(5):1751-6.

39. Eisenhauer EA, Therasse P, Bogaerts J, Schwartz LH, Sargent D, Ford R, et al. New response evaluation criteria in solid tumours: revised RECIST guideline (version 1.1). Eur J Cancer 2009;45(2):228-47.

40. Rustin GJ, Timmers P, Nelstrop A, Shreeves G, Bentzen SM, Baron B, et al. Comparison of CA-125 and standard definitions of progression of ovarian cancer in the intergroup trial of cisplatin and paclitaxel versus cisplatin and cyclophosphamide. J Clin Oncol 2006;24(1):45-51.

41. Vermorken JB, Parmar MK, Brady MF, Eisenhauer EA, Hogberg T, Ozols RF, et al. Clinical trials in ovarian carcinoma: study methodology. Ann Oncol 2005;16 Suppl 8:viii20-viii29.

42. U.S. Department of Health and Human Services, National Institutes of Health National Cancer Institute. Common Terminology Criteria for Adverse Events (CTCAE). Version 4.02. 28 May 2009.

43. Smith TJ, Khatcheressian J, Lyman GH, Ozer H, Armitage JO, Balducci L, et al. 2006 update of recommendations for the use of white blood cell growth factors: an evidence-based clinical practice guideline. J Clin Oncol 2006;24(19):3187-205.

44. Rustin GJ. Use of CA-125 to assess response to new agents in ovarian cancer trials. J Clin Oncol 2003;21(10 Suppl):187s-193s.

45. Herson J. Data and Safety Monitoring Committees in Clinical Trials. New York: CRC Press; 2009.

15. APPENDICES

15.1 Eastern Cooperative Oncology Group (ECOG) Scale for Performance Status

Grade	Description
0	Normal activity. Fully active, able to carry on all predisease performance without restriction.
1	Symptoms but ambulatory. Restricted in physically strenuous activity, but ambulatory and able to carry out work of a light or sedentary nature (eg, light housework, office work).
2	In bed < 50% of the time. Ambulatory and capable of all self-care, but unable to carry out any work activities. Up and about more than 50% of waking hours.
3	In bed > 50% of the time. Capable of only limited self-care, confined to bed or chair more than 50% of waking hours.
4	100% bedridden. Completely disabled. Cannot carry on any self-care. Totally confined to bed or chair.
5	Dead

Source: Oken MM, Creech RH, Tormey DC, Horton J, Davis TE, McFadden ET et al. Toxicity and response criteria of the Eastern Cooperative Oncology Group. Am J Clin Oncol 1982; 5 (6):649-55.

15.2 Cockcroft-Gault Equation

$$\text{Creatinine Clearance} = \frac{0.85\,(140 - \text{age [yr]}) \times \text{Weight (kg)}}{\text{Serum creatinine (mg/dL)} \times 72}$$

OR

$$\frac{0.85\,(140 - \text{age [yr]}) \times \text{Weight (kg)}}{\text{Serum creatinine } (\mu\text{mol/L}) \times 0.81}$$

Source: Cockcroft DW, Gault MH. Prediction of creatinine clearance from serum creatinine. Nephron 1976;16(1):31-41.

15.3 Modified Response Evaluation Criteria in Solid Tumors (RECIST, Version 1.1)

Disease response will be assessed using the Response Evaluation Criteria in Solid Tumor (RECIST) guidelines, version 1.1.[(39)]

15.4 Gynecologic Cancer Intergroup (GCIG) Modified Response Criteria

Table 1. GCIG Definition of Progression of Ovarian Cancer According to Both Clinical and CA-125 Criteria

Criteria	Group A	Group B	Group C
Measurable/ assessable disease	Compared with baseline (or lowest sum while on study if less than baseline), a 20% increase in sum of longest diameters (RECIST definition) or Any new lesions (measurable or non-measurable) Date of PD Date of documentation of increase or new lesions		
CA-125	CA-125 elevated pre-treatment but later normalizes<br>CA-125 ≥ 2 × ULN documented on two occasions*<br>Date PD: first date of the CA-125 elevation to ≥ 2 × ULN	CA-125 elevated pretreatment and does not normalize<br>CA-125 ≥ 2 × nadir value on two occasions*<br>Date PD: first date of the CA-125 elevation to ≥ 2 × nadir value	CA-125 in normal range pretreatment<br>As for group A

NOTE. Modified from Vergote et al.[5] Patient groups A, B, and C are defined according to CA-125 behavior during first-line therapy. A patient may be declared to have PD on the basis of either the objective disease or the CA-125 criteria. The date of PD will be the date of the earlier of the two events if both are documented.
Abbreviations: GCIG, Gynecologic Cancer Intergroup; RECIST, Response Evaluation Criteria in Solid Tumors Group; ULN, upper limit of normal; PD, progressive disease.
*Repeat CA-125 any time, but normally not less than 1 week after the first elevated CA-125 level. CA-125 levels sampled after administration of mouse antibodies or within 4 weeks after surgery or paracentesis should not be taken into account.

Rustin GJS, Timmers P, Nelstrop A, Shreeves G, Bentzen SM, Baron B, et al. Comparison of CA-125 and standard definitions of progression of ovarian cancer in the intergroup trial of cisplatin and paclitaxel versus cisplatin and cyclophosphamide. J Clin Oncol 2006; 24:45-51.

15.5 Distribution of Active Bone Marrow in the Adult

Site	% of Total
Cranium and Mandible	13
Humeri, Scapulae, Clavicles	8
Sternum and Ribs	10
Vertebrae	28
Pelvic Bones	34
Femur	4

Source: Adapted from Ellis RE: The distribution of active bone marrow in the adult. Phys Med Bio 5:255, 1960-61.

15.6 Phase 1 – Schedule of Pharmacokinetic Sampling for Paclitaxel and MLN8237

Day	Dosing		Paclitaxel PK (Cycle 1 and Cycle 2)		MLN8237 PK (Cycle 1)	
	MLN8237	Paclitaxel	Timepoint	Sample	Timepoint	Sample
1	X		Predose	X[a]	Predose	X[b]
		X (start)			1 h (± 10 min)	X[d]
		X (end)[c]	End of infusion	X[e]	2 h (± 20 min)	X[d]
			5 (± 1) min	X[f]		
			15 (± 3) min	X[f]		
			0.5 h (± 5 min)	X[f]		
			1 h (± 10 min)	X[f]	3 h (± 20 min)	X[d]
			2 h (± 20 min)	X[f]	4 h (± 20 min)	X[d]
			3 h (± 20 min)	X[f]	5 h (± 20 min)	X[d]
			7 h (± 30 min)	X[f]	9 h (± 30 min)	X[d]
			10 h (± 30 min)	X[f]	12 (± 1) h	X[d]
2	X		23 (± 2) h	X[f]		
3	X		47 (± 4) h	X[f]	Predose	X[b,g]
					1 h (± 10 min)	X[d]
					2h (± 20 min)	X[d]
					3 h (± 20 min)	X[d]
					4 h (± 20 min)	X[d]
					5 h (± 20 min)	X[d]
					9 h (± 30 min)	X[d]
					12 (± 1) h	X[d]

Abbreviations: h = hour(s); min = minute(s); PK = pharmacokinetics.

a Within 1 h before the start of the paclitaxel infusion.

b Immediately before the morning dose of MLN8237. On the day when both paclitaxel and MLN8237 are administered, MLN8237 should be administered 1 h before the start of the paclitaxel infusion.

c Paclitaxel should be infused over 60 (± 10) minutes.

d After the morning dose of MLN8237.

e Immediately before switching off the infusion pump.

f After the completion of the paclitaxel infusion (ie, from the time the infusion pump is switched off).

g The predose sample for MLN8237 PK on Day 3 can be timed to coincide with the 47-h paclitaxel PK sample depending on the timing of MLN8237 dosing.

15.7 Amendment 1 Rationale and Purposes

Rationale for Amendment 1:

The primary purpose of this amendment is to change the MLN8237 study drug to a new formulation, manufactured as an enteric-coated tablet (ECT). [redacted] new formulation of MLN8237 as an ECT has been developed recently that is intended to replace the PIC formulation for future development and commercialization. [redacted] A common formulation process and materials were used in manufacturing the unit dose strengths for the tablets planned for this study (10 or 50 mg ECT). Both tablet strengths exhibited similar dissolution properties. These features support the expectation of consistent dissolution and absorption, PK properties, and consequently, consistency within and across patients in the maintenance of desired bioactive and tolerable systemic exposures after administration of equivalent total doses with the different strength tablets.

Preliminary PK results from the ongoing phase 1 study C14001 using the ECT formulation as a single agent indicate that the steady-state MLN8237 exposures (AUC and C_{max}), resulting from 10 or 20 mg PO BID of ECT, were comparable to or less than exposures found to be well tolerated in multiple patient cohorts treated with the PIC formulation. Dose-normalized steady-state exposures of MLN8237 following ECT administration at these dose levels of 10 mg or 20 mg PO BID were within the range of observed dose-normalized steady-state exposures following administration of the PIC formulation.

Data from a series of nonclinical studies conducted to evaluate the in vivo administration of MLN8237 combined with taxanes support favorable antitumor activity coupled with improved safety with the use of an intermittent, instead of continuous, dosing schedule for MLN8237; thus, the modified MLN8237 dosing schedule to be evaluated in this amended protocol is an intermittent schedule that reduces dosing to 9 days per treatment cycle, ie, MLN8237 orally (PO) on Days 1 through 3, 8 through 10, and 15 through 17 (3 days on/4 days off), which coincides with weekly paclitaxel administered on Days 1, 8, and 15. The protocol has been amended by modifying the dosage regimen from 14 consecutive days of dosing followed by a 14-day rest period to an administration schedule of 3 days on treatment followed by a 4-day treatment-free period, repeated weekly for up to 3 weeks per 28-day treatment cycle.

The PK sampling schedules enabling the assessment of the effect of concomitant administration of MLN8237 on weekly paclitaxel characterized during the Phase 1 portion of the study have been modified because of the change in the MLN8237 dosing schedule from 14 consecutive days of dosing to intermittent dosing in 28-day treatment cycles. In addition, during the Phase 2 portion, the date and time of administration of MLN8237 doses immediately preceding each PK sample may be based on site source documentation or the patient’s dosing diary.

The changes introduced in this amendment are designed to enable the assessment of the

safety and tolerability and determination of the RP2D of combination treatment with MLN8237 ECT and paclitaxel. The changes are not predicted to adversely influence the overall risk to enrolled patients. Conduct of the study will include continuous review of the available safety and PK findings by the sponsor, participating investigators, and the Safety Monitoring Committee (SMC).

Purposes for Amendment 1:

- To change the study drug from the PIC to the ECT formulation.
- To update the protocol title to clarify that patients with ovarian or breast cancer will be included in the Phase 1 dose-escalation portion of the study, and the Phase 2 portion of the study has a randomized design and will include patients with ovarian cancer, to include epithelial adenocarcinoma of the ovary, fallopian tube, or peritoneum.
- To update the background and study rationale section describing Aurora A kinase and the Aurora A kinase inhibitor MLN8237 based on recent clinical data from ongoing MLN8237 clinical studies.
- To modify the MLN8237 dosing schedule from consecutive daily dosing to intermittent dosing.
- To update the PK assessment of concomitant administration of MLN8237 and weekly paclitaxel based on the change in dosing schedule of MLN8237.
- To increase the sample size in Phase 1 from 20 to 30.
- To decrease the number of study centers in Phase 2 from 50 to 30.
- To increase the duration of the Phase 1 portion from 18 to 22 months, including an increase in the duration of the enrollment period from 6 to 10 months.
- To update and clarify the diagnoses of patients enrolled in the Phase 1 and Phase 2 portions of the study.
- To add an inclusion criterion regarding prior treatment for patients with breast cancer.
- To clarify the inclusion criterion that measurable disease in patients with breast cancer is according to RECIST, version 1.1.
- To clarify the inclusion criteria regarding antineoplastic therapy and hormonal therapy.
- To add a requirement for a baseline hemoglobin level of $\geq$ 9 g/dL.
- To clarify that creatinine clearance can be calculated using serum creatinine.
- To update the period of contraception to which the patient agrees by changing the period after the last dose of study drug from 3 months to 30 days, which is in alignment with other protocols.
- To clarify the specific inclusion criteria for patients with recurrent ovarian, fallopian tube, or peritoneal cancer.
- To clarify what is meant by cytotoxic agent, chemotherapy regimen, and hormonal therapy with regard to exclusion criteria.
- To update exclusion criteria relating to central nervous system malignancy or metastatic disease in alignment with updated protocol template language.
- To update the exclusion criterion relating to other medical conditions.

- To delete the exclusion criterion excluding patients receiving systemic anticoagulation.
- To clarify that the exclusion criterion for infection refers to systemic infection.
- To clarify paclitaxel administration during Phase 1 by updating the language to specify an infusion time of 1 hour and updating language regarding modification of the infusion time.
- To clarify that antiemetic agents are not to be used prophylactically during Cycle 1 by updating the language to specify that prophylactic antiemetic agents should not be used in the first cycle of treatment if nausea or vomiting is not observed.
- To update the version of the National Cancer Institute Common Terminology Criteria for Adverse Events (NCI CTCAE) from version 3.0 to version 4.02, effective as of 01 October 2009 for evaluation of toxicity in this study.
- To update the list of definitions of dose-limiting toxicity (DLT) by updating or adding language to clarify the events in the list of definitions of DLT.
- To include investigators along with the sponsor in the agreement to evaluate new cohorts in the event that MLN8237 and paclitaxel 80 mg/m^2 is not tolerated.
- To update and clarify dose modifications for hematological toxicity.
- In the criteria for retreatment and dose delays, to clarify the statement regarding somnolence, and to clarify the process regarding the decision to continue treatment after repeat toxicities and multiple dose reductions.
- To clarify the use of concomitant medications with central nervous system (CNS) effects. Medications with potential CNS effects are not prohibited in this study, but it is recommended that their use be minimized to avoid confusion in the interpretation of CNS effects should they occur during the course of treatment with MLN8237. The language regarding concomitant medications with CNS is updated to include typical or atypical antipsychotic and antidepressant agents.
- To update the precautions and restrictions associated with MLN8237; based on updated information, the precaution regarding the theoretical risk of dependency and withdrawal symptoms associated with MLN8237 is removed.
- To update the section Study Personnel and Organizations in alignment with updated protocol template regulations, as study personnel and contact information may change. This information is located in the Study Manual or other supporting documents.
- To add language to clarify the visit windows and that any Cycle 1, Day 1 visit procedures also required at screening can be skipped if the screening procedures were performed within 4 days prior to the Cycle 1, Day 1 dose of study drug. The window for the Cycle 1, Day 1 visit has been changed from 3 days to 4 days to allow time to receive test results before the weekend and determine eligibility for enrollment in the study.
- To remove the Baseline visit, as Cycle 1, Day 1 is baseline, and the representation of the Baseline visit as a separate visit in the Schedules of Events is cause for confusion.
- To add FIGO (International Federation of Gynecology and Obstetrics) stage and BRCA (breast cancer gene), ER and PR (estrogen and progesterone receptors), and HER2 (human epidermal growth factor receptor 2) status to the medical history.

- To add measurement of thyroid stimulating hormone (TSH) as a safety laboratory test to be done at baseline, EOT, and during the dosing period if clinically indicated.
- To update and clarify obtaining archival tumor samples for determination of expression of Aurora A kinase.
- To change the timing of 1 of the blood samples for serum markers of response from Cycle 1 to Cycle 2, as the effect on these markers may not be observed until after Cycle 1.
- To clarify what is meant by a patient who “tolerates” study treatment as related to the Day 21 visit becoming optional, language has been added to define “tolerate” as “without the requirement for dose reduction or without ≥ Grade 3 treatment-related toxicity evaluated according to NCI CTCAE, version 4.02”.
- To change evaluation of clinical laboratory tests from central to local in Phase 1; thus, local laboratories are used in Phase 1, and a central laboratory is used in Phase 2.
- To clarify that an elevated CA-125 level is > 70 units/mL.
- To clarify that modified Gynecologic Cancer Intergroup (GCIG) criteria are used.
- To change the reporting period for nonserious adverse events from “through 30 days after the last dose of study drug” to “through 30 days after the last dose of study drug or until the start of subsequent antineoplastic therapy, whichever occurs first”.
- To change the reporting period for concomitant medications from “through the end of treatment” to “through 30 days after the last dose of study drug or until the start of subsequent antineoplastic therapy, whichever occurs first” to align with collection of adverse event (AE) information.
- To update procedures for study compliance by adding language to allow verbal confirmation of the medication count for patients who do not require a clinic visit on Day 21, with confirmation at the subsequent clinic visit.
- To update the section Discontinuation of Study Drug and Patient Replacement by amending the list of reasons and updating accompanying language for this section. The rule for patient replacement has been updated based on the updated dosing schedule.
- To update the section regarding withdrawal of patients from study follow-up.
- To update the SMC membership to include physicians from the study team and Pharmacovigilance and staff from Clinical Operations.
- To add a new protocol section regarding the reporting of suspected unexpected serious adverse reactions (SUSARs).
- To provide information on how to report medication errors.
- To shorten the Use of Information section by deleting the nonmandatory sections in this section.
- Correct typographical errors, punctuation, grammar, and formatting.

15.8 Amendment 2 Rationale and Purposes

Rationale for Amendment 2:

The primary purpose of this amendment is to make required updates realized once the study began enrollment, and to correct discrepancies noticed after finalization.

Recent clinical evidence supports the potential clinical utility of weekly taxane regimens, administered first line in combination with platinum chemotherapy. For some patients who formerly received weekly taxane, retreatment with a weekly taxane regimen also remains a clinical treatment option in the relapsed setting, so the eligibility is modified to allow prior weekly taxane. Given potential difference in outcomes based on prior treatment with weekly taxane, in the Phase 2 part of the study the randomization will be stratified by prior treatment with weekly taxane. The stratification by clear cell histology has been removed.

Emerging data indicates that ovarian, breast, or other cancers may have different pathogenetic mechanisms if they arise in the setting of BRCA mutations.(1) [redacted] nd this testing uses methodology that is not employed for clinical patient management. Testing of tumor tissue may be employed for patients enrolled in either the Phase 1 or Phase 2 portion of the study. In addition, the blood sample already planned for pharmacogenomic analyses may be assessed for [redacted] may not be performed if this information is otherwise documented in the clinical records for entry on the case report form (CRF). The blood test may employ methodologies that are also used clinically. Study sites unable to perform [redacted] for any reason may continue to enroll subjects into the study.

An exclusion criterion is added for concomitant medicines known to be uridine diphosphate glucuronosyltransferase (UGT) or cytochrome P450 (CYP) inducers. The clearance of MLN8237 in humans is expected to be mediated primarily by glucuronidation, with a minor contribution of CYP-mediated oxidative metabolism, based on in vitro drug metabolism studies. The effect of drug-metabolizing enzyme inducers on the clinical pharmacokinetics of MLN8237 has not been characterized. Therefore the possibility of decreased systemic exposures of MLN8237 in patients receiving clinically significant UGT/CYP inducers cannot be excluded. Consequently, this amendment specifies exclusion of patients treated with the enzyme-inducing antiepileptic drugs phenytoin, carbamazepine, or phenobarbital; or rifampin, rifabutin, rifapentine, or St. John's Wort within 14 days prior to the first dose of MLN8237 and specifies that co-administration of these drugs is not permitted during participation in the study.

Recent phase 2 experience with MLN8237 for treatment of relapsed ovarian cancer indicates

that this agent can lead to objective responses observed by RECIST and/or CA-125 criteria within the first few treatment cycles; however, in some patients, MLN8237 treatment leads to sustained disease control or objective response detected only after multiple months of treatment.[3] These latter clinical findings may reflect favorable biologic activity by MLN8237 to produce senescence in tumor cells, as reported in model systems, which may not immediately reduce the size of tumor cells that constitute target lesions.[4] Eligibility for this protocol will include patients who may have few other treatment options for management of progressive malignancy. Thus, this amendment allows patients with sustained stable disease (SD) or response to continue protocol treatment beyond 12 months up to 24 months if the investigator determines that a patient would derive benefit from continued treatment. If it is determined that a patient tolerates MLN8237 treatment and would derive benefit from continued treatment beyond 24 months, MLN8237 may be continued in this or in another extension or rollover study, if available, upon request by the investigator and agreement by the medical monitor.

Similarly, this amendment allows a patient who experiences progressive disease (PD), defined by CA-125 criteria or imaging results described in the protocol (Section 15.4), to continue protocol treatment if tolerated and if the investigator determines it is in the patient's best interest upon review and agreement by the medical monitor. Serial objective monitoring will continue according to the Schedules of Events. Statistical analysis of clinical outcomes (eg, time to progression) will continue to employ the predefined definitions for PD (RECIST or GCIG CA-125, Section 15.4) even if the patient continues protocol treatment after PD. In this setting, the date and criteria for the original determination of PD per protocol will be recorded in the CRF. In addition, the date and criteria for subsequent treatment discontinuation will be recorded in the CRF in addition to dosing history and serial evaluations of disease, including CA-125 and imaging results. Subsequent discontinuation due to PD may be determined based on consideration of clinical findings, including the following: a) disease progression reconfirmed over 2 or more additional response assessments; b) continued progression of target lesions by over 20% increase in sum of product diameters compared to baseline (instead of comparison to best response); c) detection of unequivocal, new metastases detected in visceral organs, such as lung or liver; d) progression that is symptomatic or leads to altered organ function, such as increases in bilirubin by 1 CTC severity grade that could represent altered drug clearance.

Purposes for Amendment 2

- To specify that patients in the Phase 1 portion can be followed until 110 progression-free survival events are achieved in Phase 2.
- To add antineoplastic therapy as a reason to discontinue treatment with MLN8237, paclitaxel, or combination therapy (MLN8237 + paclitaxel).
- To clarify that the 23- and 47-hour pharmacokinetic blood samples are to be drawn prior to MLN8237 dosing on days where PK draws are required.
- To add assessment of [redacted] in the Phase 2 portion of the study.
- To ensure 20-mg dexamethasone should be used throughout the protocol if premedication is required for the paclitaxel.

- To clarify the exclusion criterion of prior treatment with an Aurora A-targeted agent (including MLN8237). The exclusion of prior treatment with weekly taxane has been removed.
- To modify the stratification factors.
- To add exclusion criterion for UGT and CYP inducers.
- To clarify that if a standard of care result for CA-125 is used for inclusion into the study, the value should be obtained within 9 days of screening and sufficient time after prior therapy.
- To clarify that the Phase 1 cohorts may include re-escalation of the MLN8237 starting dose to determine MTD of the combination with a reduced starting dose of paclitaxel.
- To allow continued protocol treatment up to 24 months (or longer if the investigator determines that the patient would derive benefit from continued protocol treatment upon review by the medical monitor).
- To change the sample size in the Phase 2 portion to better control the type I error.
- To change the statistical test for the time-to-event endpoints to the unstratified log rank test as it is more powerful when there are small size strata.
- To include the medical monitor or designee in the safety committee language.
- To update preliminary information on clinical experience.
- To update preliminary information on pharmacokinetic clinical experience.
- To update information on potential risks.
- To clarify the determination of the recommended Phase 2 dose (RP2D).
- To update the duration of study, including increasing the duration of participation in the Phase 2 portion of the study to incorporate the change in the maximum treatment duration (12 months to 24 months).
- To clarify recovery toxicity from prior therapy.
- To clarify prior treatments.
- To clarify definition of disease.
- To clarify exclusion based on prior chemotherapy.
- To clarify the exclusion based on use of proton pump inhibitors, histamine H2 antagonists, and antacids.
- To clarify the potential for reduction in MLN8237 dose and/or schedule.
- To clarify the potential for escalation of paclitaxel dose up to a maximum of 80 mg/m^2.
- To clarify the replacement of patients in Phase 1.
- To clarify the dose modification and retreatment criteria.
- To clarify the use of myeloid growth factors for the treatment of neutropenia.
- To clarify the use of body surface area (BSA) in determining the dose of pacitaxel.
- To revise the definitions on the patient populations for analysis.
- To update the description of the interim analysis.
- To correct typographical errors, punctuation, grammar, and formatting.

15.9 Amendment 3 Detailed Summary of Changes

THE PRIMARY SECTION(S) OF THE PROTOCOL AFFECTED BY THE CHANGES IN AMENDMENT 3 ARE INDICATED. THE CORRESPONDING TEXT HAS BEEN REVISED THROUGHOUT THE PROTOCOL.

Purpose: To clarify that patients may be maintained on study treatment after PD in selected circumstances, and to define the criteria for withdrawing patients from study treatment.

The primary change occurs in Section 7.5, Completion of Treatment:

Formerly read:

Treatment with MLN8237 is to be considered completed for patients meeting any of the following criteria:

- 24 months of treatment
- PD
- Symptomatic deterioration

A patient can be treated for up to 24 months. If it is determined that a patient tolerates protocol treatment and would derive benefit from continued MLN8237 treatment beyond 24 months, MLN8237 treatment may be continued in this or in another extension or rollover study, if available, upon request by the investigator and agreement by the medical monitor.

~~P~~atients~~ ~~who experience PD~~, based on ~~CA-125~~ ~~or imaging ~~according to~~ protocol~~ definitions~~ (Section 15.4)~~,~~ may continue protocol treatment if tolerated and if the investigator determines it is in the patient's best interest upon review and agreement by the medical monitor. Serial objective monitoring will continue according to the Schedules of Events. Statistical analysis of clinical outcomes (eg, time to progression) will continue to employ the predefined definitions for PD (RECIST or GCIG CA-125, Section 15.4) even if the patient continues protocol treatment after PD. In this setting, the date and criteria for the original determination of PD per protocol ~~criteria~~ will be recorded in the CRF. ~~In addition, t~~he date and criteria for subsequent treatment discontinuation will be recorded in the CRF. ~~Subsequent discontinuation due to PD may be determined based on consideration of clinical findings, including consideration of the following: a) disease progression reconfirmed over 2 or more additional response assessments; b) continued progression of target lesions by over 20% increase in sum of product diameters compared to baseline (instead of comparison to best response); c) unequivocal, new metastases detected in visceral organs such as lung or liver; d) progression that is symptomatic or leads to altered organ function, such as increases in bilirubin by 1 CTC severity grade that could represent altered drug clearance.~~

Now

Treatment with MLN8237 is to be considered completed for patients meeting

reads: any of the following criteria:

- 24 months of treatment
- PD **(see below for exception)**
- Symptomatic deterioration

A patient can be treated for up to 24 months. If it is determined that a patient tolerates protocol treatment and would derive benefit from continued MLN8237 treatment beyond 24 months, MLN8237 treatment may be continued in this or in another extension or rollover study, if available, upon request by the investigator and agreement by the medical monitor.

A patient who experience**s** PD, **as defined by** CA-125 **criteria** or imaging **results described in the** protocol (Section 15.4) may continue protocol treatment if tolerated and if the investigator determines it is in the patient's best interest upon review and agreement by the medical monitor. **Criteria for removing patients from the study include the following:**

1. **Patients with unequivocal evidence of any new metastasis including development of peritoneal studding or malignant ascites must be removed from study.**
2. **Patients whose disease progression is such that they are at risk for catastrophic complications from vital organ compression (eg, spinal cord compression, small bowel obstruction, encroachment of major blood vessels, etc.) must be removed from study immediately and treated appropriately.**
3. **Patients with evidence of progression based on RECIST must be removed from study if the subsequent assessment (8 weeks later) demonstrates evidence of progressive disease.**
4. **Patients must be informed of the evidence of progression and the availability of alternative treatments.**

In addition to the above criteria, patients who have PD that is symptomatic or leads to altered organ function, such as increases in bilirubin by 1 CTC severity grade that could represent altered drug clearance, should be removed from study.

If a patient is maintained on study treatment after evidence for PD, serial objective monitoring will continue according to the Schedules of Events. Statistical analysis of clinical outcomes (eg, time to progression) will continue to employ the predefined definitions for PD (RECIST or GCIG CA-125, Section 15.4) even if the patient continues protocol treatment after PD. In this setting, the date and criteria for the original determination of PD per protocol will be recorded in the CRF. **The** date and criteria for subsequent treatment discontinuation will be recorded in the CRF **in addition to dosing history and serial evaluations of disease, including CA-125 and imaging results.**

Purpose: To correct typographical errors, punctuation, grammar, and formatting.

These changes are not listed individually.
